# A Charge-Driven Strategy for Covalent Modification and Modulation of Biomolecular Condensates

**DOI:** 10.1021/jacs.5c06625

**Published:** 2025-08-02

**Authors:** Zhewei Chen, Lu Liu, Jerome Cattin, Tuomas P. J. Knowles, Gonçalo J. L. Bernardes

**Affiliations:** † Yusuf Hamied Department of Chemistry, 2152 University of Cambridge, Cambridge CB2 1EW, U.K.; ‡ Transition Bio, Ltd., Cambridge CB2 8DU, U.K.; § Cavendish Laboratory, Department of Physics, University of Cambridge, Cambridge CB3 0HE, U.K.; ∥ Translational Chemical Biology Group, Spanish National Cancer Research Centre (CNIO), C/Melchor Fernández Almagro, 3, 28029 Madrid, Spain

## Abstract

Small-molecule modulation of biomolecular condensates has emerged as a novel and attractive therapeutic modality. Increasing evidence implicates dysregulated condensate formation in neurodegenerative diseases and cancer. However, the proteins that mediate condensate formation are typically difficult to drug directly with small molecules. Here, we present a charge-driven strategy and demonstrate its implementation on Ras GTPase-activating protein-binding protein 1 (G3BP1) to inhibit G3BP1-mediated stress granule (SG) formation. Small-molecule SG inhibitors were developed from the carbonylacrylic amide covalent functionality and were used to modify the folded domain of G3BP1 with surface charges, leading to an alteration of the conformational dynamics of intrinsically disordered regions. Cellular experiments using HeLa cells expressing cysteine-mutated G3BP1, together with structure–activity relationship studies, support the proposed charge-driven mechanism of action. Molecular dynamics simulations further suggest that the small-molecule G3BP1 modification promotes a shift toward more compact conformations, comparable to that induced by an ∼26% increase in IDR1-IDR3 interaction. Together, our findings establish a new strategy for the rational modulation of biomolecular condensates.

## Graphical abstract

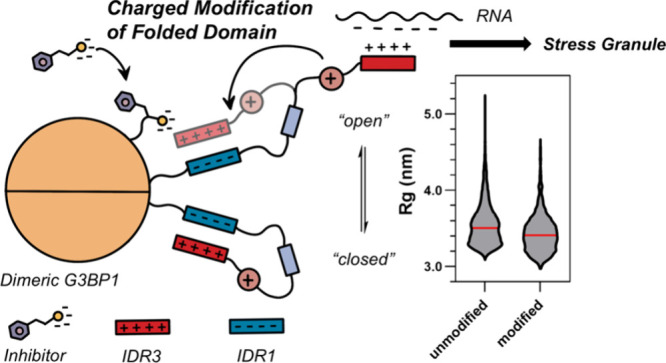

Small-molecule modulation of biomolecular condensates formed via liquid–liquid phase separation (LLPS) is an emerging therapeutic modality.^1–3^ Following seminal studies that showed how biomolecular condensates form through LLPS,^4–6^ substantial evidence has been produced supporting strong connections and frequently causality between dysregulation of LLPS and diseases such as neurodegeneration and cancer.^7–11^ Development of small-molecule modulators of LLPS can be dichotomized into those that directly target the phase-separating biomolecule or indirectly target the cellular context, including upstream regulatory nodes.^1,12^ While developing indirect modulators benefits from established methods of drug discovery,^13–16^ key mediators of LLPS are often RNA-binding proteins (RBP) and difficult-to-drug.^17,18^ However, direct on-target condensate modulators epitomize the novel target-centric drug discovery path that synergizes better with human-biology-guided research,^19^ which has been an increasingly important driver of therapeutics development.^20,21^

Direct modulators have been reported with diverse mechanisms of action (MOA), including but not limited to degradation or translocation of the target protein, modulation of protein–protein interaction, disruption of oligomerization.^22–27^ However, most of the reported direct modulators were discovered through phenotypic screening and rational design remains difficult.^28^ Challenges in drugging RBPs epitomizes the difficulties because of the critical role IDR-RNA interactions play in LLPS.^10^ RBPs generally lack good binding pockets or enzymatic activities and contain structurally dynamic intrinsically disordered regions (IDR) that interact with RNA.^29^ While peptide-based methods are promising for addressing specific issues,^24,30,31^ directly drugging RBPs, and more generally phase-separating proteins remain difficult.^32,33^ Therefore, new rational design strategies for direct on-target small-molecule modulation of LLPS are important.

We propose that these challenges could be tackled through a new strategy where instead of pursuing a binder for IDR, a binder for the folded domain (FD) is developed into a chemical probe that perturbs FD-IDR interactions to affect changes in IDR dynamics critical for LLPS. We further reason that modifying FD surface with charged moieties could be sufficient because recent studies showed FD surface charge plays a significant role in mediating phase separation through intramolecular FD-IDR charge–charge interactions.^34–37^ We aim to demonstrate the viability of this new charge-driven strategy by developing chemical probes to modulate G3BP1-mediated stress granule (SG) formation because a seminal study on SGs by Taylor et al.^38^ established that G3BP1 switches between “open” and “closed” conformations, characterized by charge–charge interactions mediated by the positively charged IDR3, to determine SG formation.

We observed that G3BP1 Cys73 is positioned with suitable geometry on the surface of the FD (NTF2-like) of G3BP1, which upon modification with negatively charged small molecules may induce stabilizing charge–charge interactions with IDR3 for the ‘closed’ conformations to tune the G3BP1 switch toward inhibition (Figure 1A). Therefore, we began our study from the cysteine-selective carbonylacrylic amide (CAA) functionality,^39,40^ which efficiently modifies G3BP1 Cys73 as verified by click-pulldown experiments in HeLa cells using an alkyne-derivative (CAA-alkyne, Figure 1B). A panel of CAA derivatives (Figure 1C) was synthesized, including CAA methyl carboxylates (CAA-A1 to A6, CAA-AA) and more hydrophobic derivatives (CAA2 to CAA-5). This panel of compounds was tested in fluorescence microscopy assays where SGs were induced in HeLa cells expressing G3BP1-mScarlet with NaAsO_2_ (Figure 2A). Intriguingly, all CAA methyl carboxylates except for CAA-A3 showed potent inhibition of G3BP1-mediated SG formation at ∼33 μM. The hydrophobic derivatives had reduced efficacy, while the lactone and quaternary ammonia derivatives were not effective (Figure 2B–C, Figure S1). We were surprised that most of the candidate modulators potently inhibit SG formation, especially at inhibition by the structurally simple CAA-alkyne. This finding echoes recent reports hinting at large number of potential targets or pathways that could induce or inhibit SGs.^15,41–43^ We then tested a panel of electrophilic covalent chemical probes to find that only a CAA-like probe inhibited G3BP1-mediated SG formation (Figure S2). Therefore, we suspect that SG inhibition induced by CAA-alkyne may originate from its off-target (G3BP1) reactivities. To investigate this, we proceeded to investigate the MOA of both CAA-alkyne and a representative CAA methyl carboxylate (CAA-AA).

**Figure 1:**
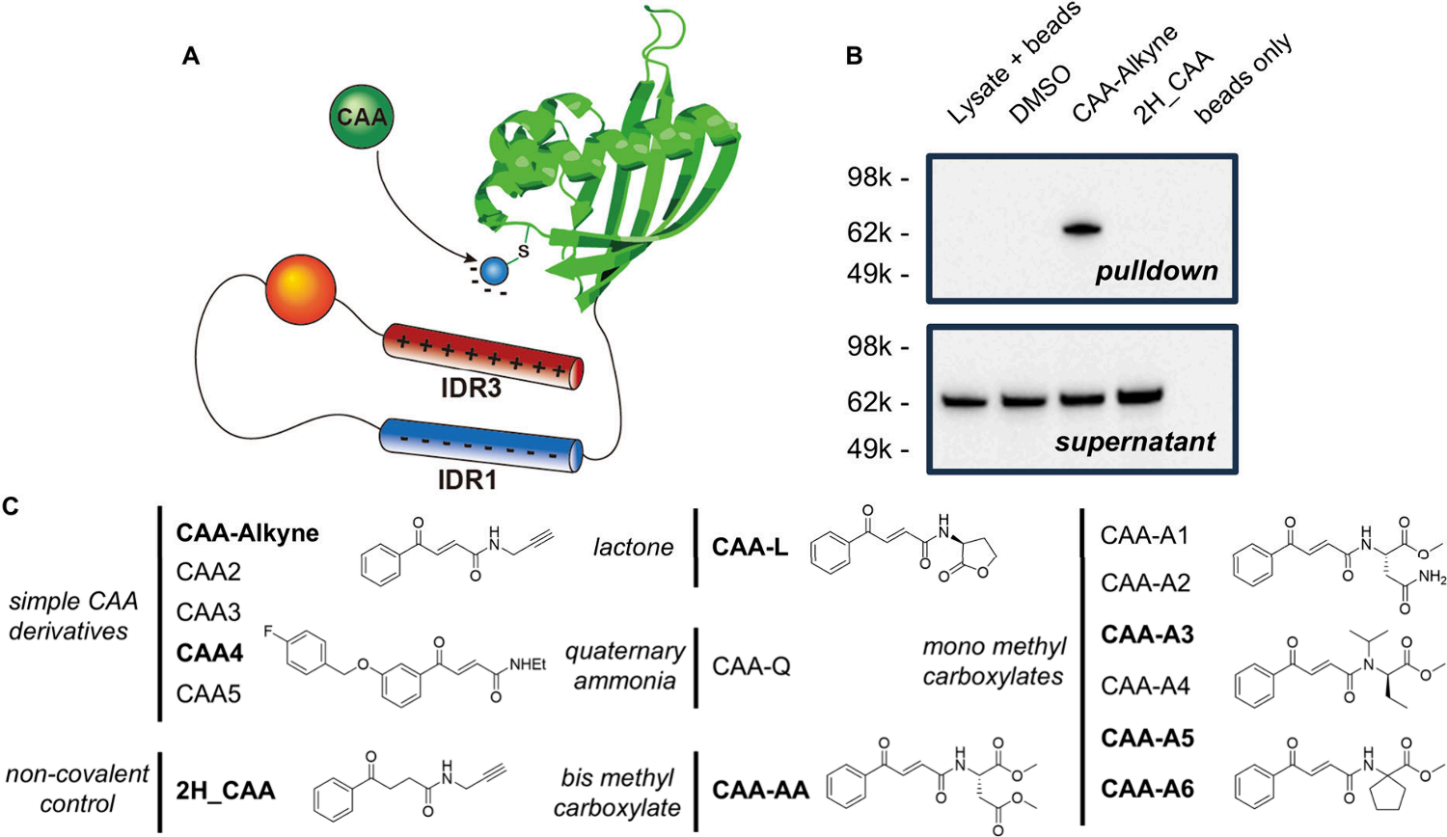
Concept and design of the charge-driven strategy. (A) Modification of G3BP1 Cys73 with negatively charged moieties is proposed to promote the ‘closed’ conformations, thereby inhibiting G3BP1-mediated SG formation. (B) Immunoblot analysis of G3BP1 from click-pulldown experiments in HeLa cells. (C) Designs of CAA-derived candidate modulators. Structures of selected compounds (in bold) are shown.

**Figure 2:**
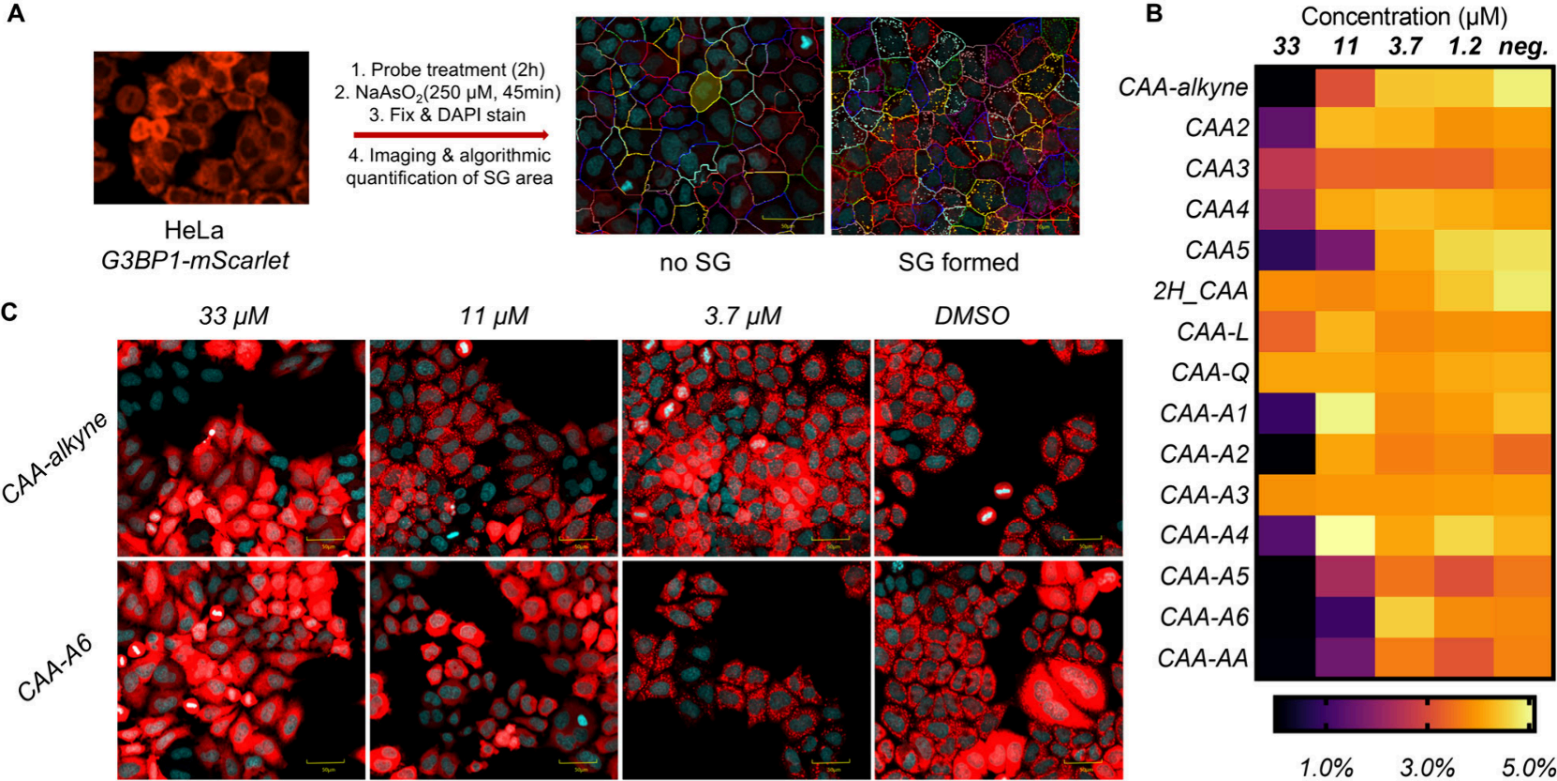
Characterization of SG inhibition by fluorescence microscopy. (A) Workflow. (B) 14 covalent and 1 noncovalent (2H_CAA) derivatives of CAA were screened. SG area is reported as a percentage of total cell area. (C) Example microscopy images showing inhibition of G3BP1-mediated SG formation. Scale-bar, 50 μm. Red, G3BP1-mScarlet; blue, DAPI.

As SG formation in response to acute peroxide stress is cytoprotective and antiapoptotic,^44^ measuring the sensitivity of cells to acute H_2_O_2_ stress by cell viability is a functional assay of SG inhibition that complements the microscopy findings (Figure 3A). Assay results showed that treatment with 30 μM CAA-alkyne or CAA-AA increased sensitivity to H_2_O_2_ by ∼40% and ∼80%, respectively (Figure 3B, Figure S3), suggesting that both compounds induce functionally relevant SG inhibition. Moreover, agreement in threshold concentrations from the functional assays and the microscopy assays (Figure 3C, Figure S3) suggest that both assays are suitable for characterizing G3BP1-mediated SG inhibition. To determine if SG formation in response to acute NaAsO_2_ stress is also cytoprotective and antiapoptotic, we repeated the stress sensitization assays with NaAsO_2_ stress. However, assay results suggest that SG inhibition induced by the CAA-derived modulators desensitizes HeLa toward acute NaAsO_2_ stress (Figure 3B–C, Figure S3), consistent with known differences in SGs formed in response to the two oxidative stresses.^44^ Although the origin of this difference is beyond the scope of this study, we hypothesize that it reflects differential stress-response pathways activated by recoverable versus irrecoverable stress conditions.

**Figure 3:**
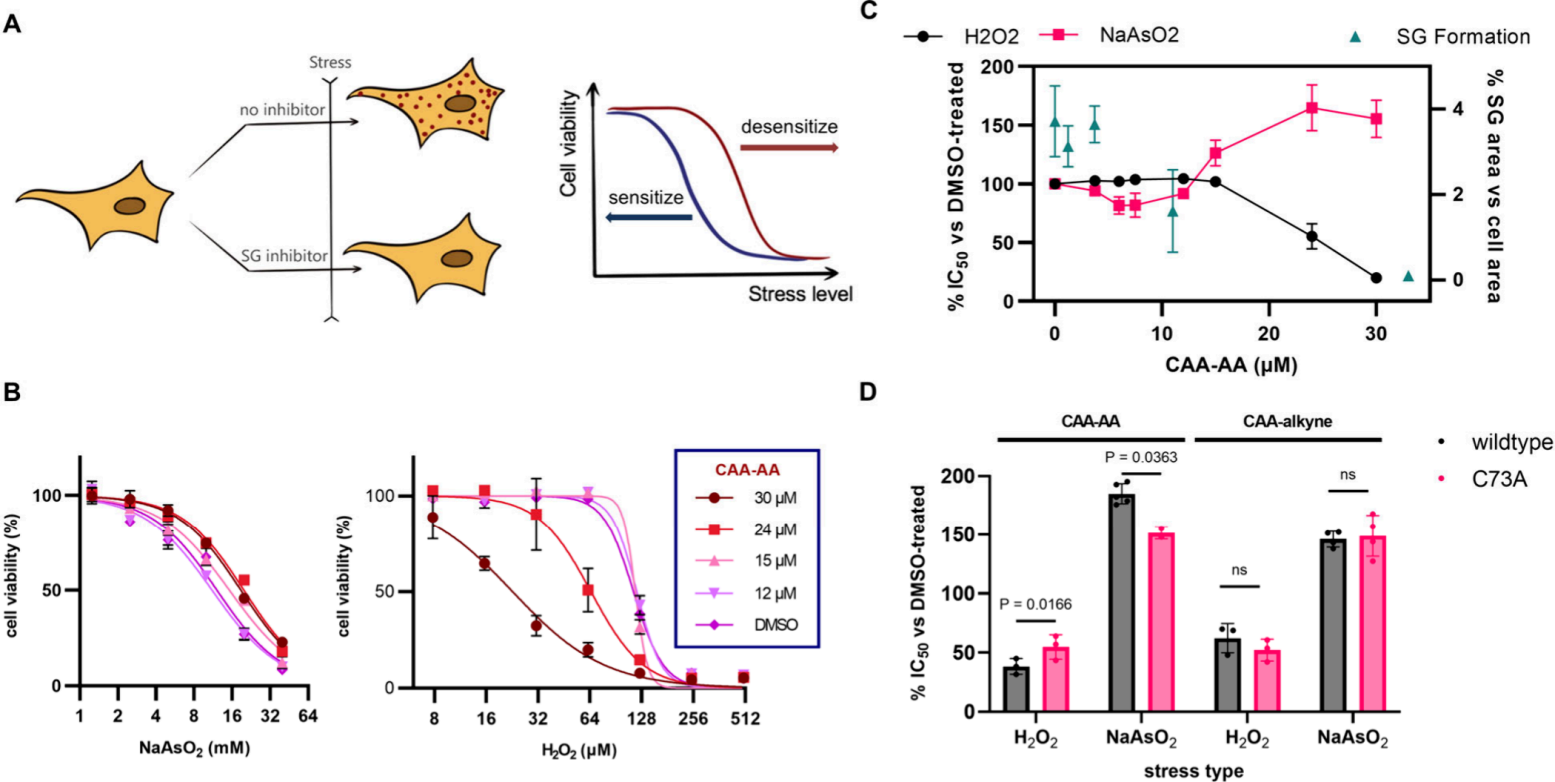
Stress sensitization assays. (A) Workflow. Cells pretreated with SG inhibitors (2 h) were exposed to acute stresses (2 h). Cell viability readouts gave quantification of sensitization/desensitization. (B) Cell viability readouts for HeLa treated with CAA-AA. (C) Stress sensitization/desensitization (left *y*-axis) and SG formation (right *y*-axis) across varying CAA-AA concentrations. (D) Head-to-head comparison of sensitization/desensitization to H_2_O_2_/NaAsO_2_ induced by CAA-AA (30/20 μM) and by CAA-alkyne (30/40 μM) in HeLa cell lines expressing either C73A-G3BP1 or wildtype-G3BP1, constructed in parallel. Ratio paired parametric multiple comparison *t*-test; ns, *p* > 0.05.

To determine if CAA-alkyne and CAA-AA act directly on G3BP1 to inhibit G3BP1-mediated SG formation, HeLa cell lines stably expressing wildtype or C73A-mutated G3BP1 were constructed using CRISPR and PiggyBac transposon methods in parallel (SI-methods). As CAA is cysteine-selective,^39^ the C73A mutation abrogates CAA modification of G3BP1. Using both cell lines, CAA-alkyne and CAA-AA were tested in stress sensitization assays which provided higher data quality than microscopy-based quantification. Head-to-head comparison experiments showed that the C73A mutation caused statistically significant reductions in sensitization to H_2_O_2_ in HeLa treated with CAA-AA (Figure 3D). On the contrary, the mutation had no statistically significant effect in HeLa treated with CAA-alkyne (Figure 3D). To determine if G3BP2 expression caused false-negative results with CAA-alkyne, the experiments with CAA-alkyne were repeated with RNAi knockdown of G3BP2 expression. Again, there was no difference in sensitization (Figure S4). These experiments strongly suggest that CAA-AA acts directly on G3BP1 to modulate its phase separation, while CAA-alkyne acts independently of G3BP1. Results from experiments with NaAsO_2_ stress also support these conclusions (Figure 3D). The contrasting results from CAA-AA and CAA-alkyne suggest that covalent modification of Cys73 by itself is not sufficient to cause SG inhibition and that the methyl ester functional groups are key for inducing SG inhibition through G3BP1 modification.

To investigate whether the MOA of CAA-AA is charge-driven by design, we synthesized more difficult-to-hydrolyze derivatives of CAA esters (CAA-AAR, CAA-Asp2R) and a tris-methyl carboxylate derivative designed to install a higher density of charged functional groups on G3BP1 (Figure 4A). Because the cells were pretreated with the CAA derivative for only 2 h before the introduction of stresses in the stress sensitization experiments, we do not expect significant hydrolysis of CAA-AAR and CAA-Asp2R. H_2_O_2_ sensitization assays with the more difficult-to-hydrolyze derivatives showed that the C73A mutation no longer reduced sensitization to H_2_O_2_ with the introduction of difficult-to-hydrolyze esters (Figure 4B). Interestingly, even when pretreatment time was extended to 16 h for CAA-Asp2R, no difference in sensitization was observed between wildtype and C73A-expressing cells. In contrast, a significant difference in sensitization was observed in cells treated with CAA-Asp2. These findings showed that easy-to-hydrolyze esters are necessary for modulating G3BP1-mediated SG formation through G3BP1, and strongly support a charge-dependent MOA for CAA-AA.

**Figure 4:**
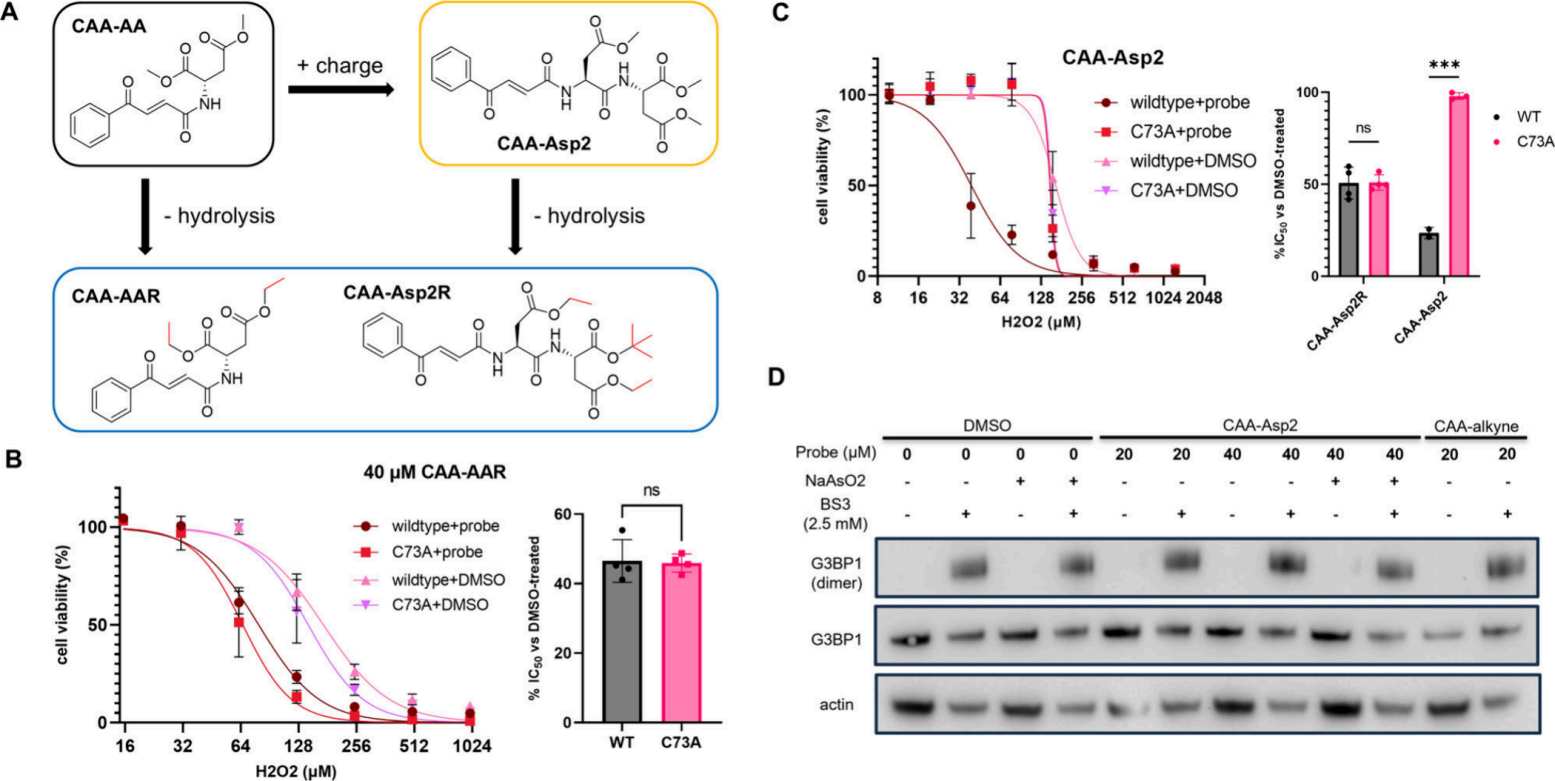
Mechanistic studies on CAA-AA. (A) Structures of CAA-AA derivatives synthesized for mechanistic investigations. (B–C) H_2_O_2_ sensitization assays using HeLa cell lines expressing C73A-G3BP1 or wildtype-G3BP1. (B) Cells pretreated with 40 μM CAA-AAR for 2 h. (C) Cells pretreated with CAA-Asp2 (40 μM, 2 h) or CAA-Asp2R (10 μM, 16 h). *** = *p* < 0.001. (D) BS3 cross-linking experiments with CAA-Asp2 and CAA-alkyne; immunoblots against G3BP1.

While a charge-driven MOA for CAA-AA hints at stabilization of the ‘closed’ conformations, an alternative MOA involving allosteric disruption of G3BP1 dimerization needs to be considered. BS3-cross-linking experiments in HeLa cells showed that G3BP1 dimer formation was not affected by CAA-derived inhibitors (Figure 4D). This result was supported by RFP-pulldown experiments performed on HeLa expressing G3BP1-mScarlet (Figure S5).

Finally, we performed coarse-grained molecular dynamics (CG-MD) simulations to evaluate the impact of Cys73 modification by CAA-AA on IDR conformation dynamics of dimeric G3BP1 (SI-methods for details). While MD simulation that does not consider RNA-G3BP1 interactions will not fully capture G3BP1’s conformation dynamics in the cell, the working model of G3BP1 tuning of SG through its IDR conformations^45^ allows us to distill the problem into evaluating G3BP1 IDR conformation dynamics as a predictor of G3BP1-mediated SG formation. The structure of CAA-AA-modified dimeric G3BP1 was modeled based on AlphaFold-predicted structure of G3BP1 with Rosetta using the generalizedKIC method.^46,47^ The best-scored structure was then coarse-grained using the Martini3 force field with an elastic network for MD simulations in GROMACS.^48,49^ Adapting MD parameters previously reported for modeling FD-IDR interactions,^34^ 10-μs MD simulations on unmodified and CAA-AA-modified dimeric G3BP1 were performed (Figure 5A-B, Figure S6). To evaluate the dynamics between “open” and “closed” conformations, the observables of radius of gyration (Rg) and number of IDR1-IDR3 contacts were extracted and analyzed (Figure 5A-C). As Rg measures the compactness of the protein, a shift in conformation dynamics to favor the “closed” conformation should be characterized by decreased Rg and increased total contacts for unmodified dimeric G3BP1. After addressing autocorrelation by blocking with a block-size of 2-μs (SI-methods), a clear linear inverse proportional relationship emerges between Rg and total contacts for unmodified dimeric G3BP1 (Figure 5D), which was leveraged for estimating the effective impact of CAA-AA acid modification. As the simulation data are highly autocorrelated, the Rg data were analyzed with autoregressive modeling (SI-methods, Figure S7) and the expected average Rg shift was calculated from simulated time series (Figure 5E) to give a Δ⟨Rg⟩ estimate of 0.115 ± 0.0615 nm for a typical 10-μs simulation. Interpreting Rg-total-contacts relationship linearly, this translates into a ∼ 26% increase in IDR1-IDR3 interaction for dimeric G3BP1 modified with CAA-AA acid, supporting the viability of the charge-driven modulation strategy.

**Figure 5:**
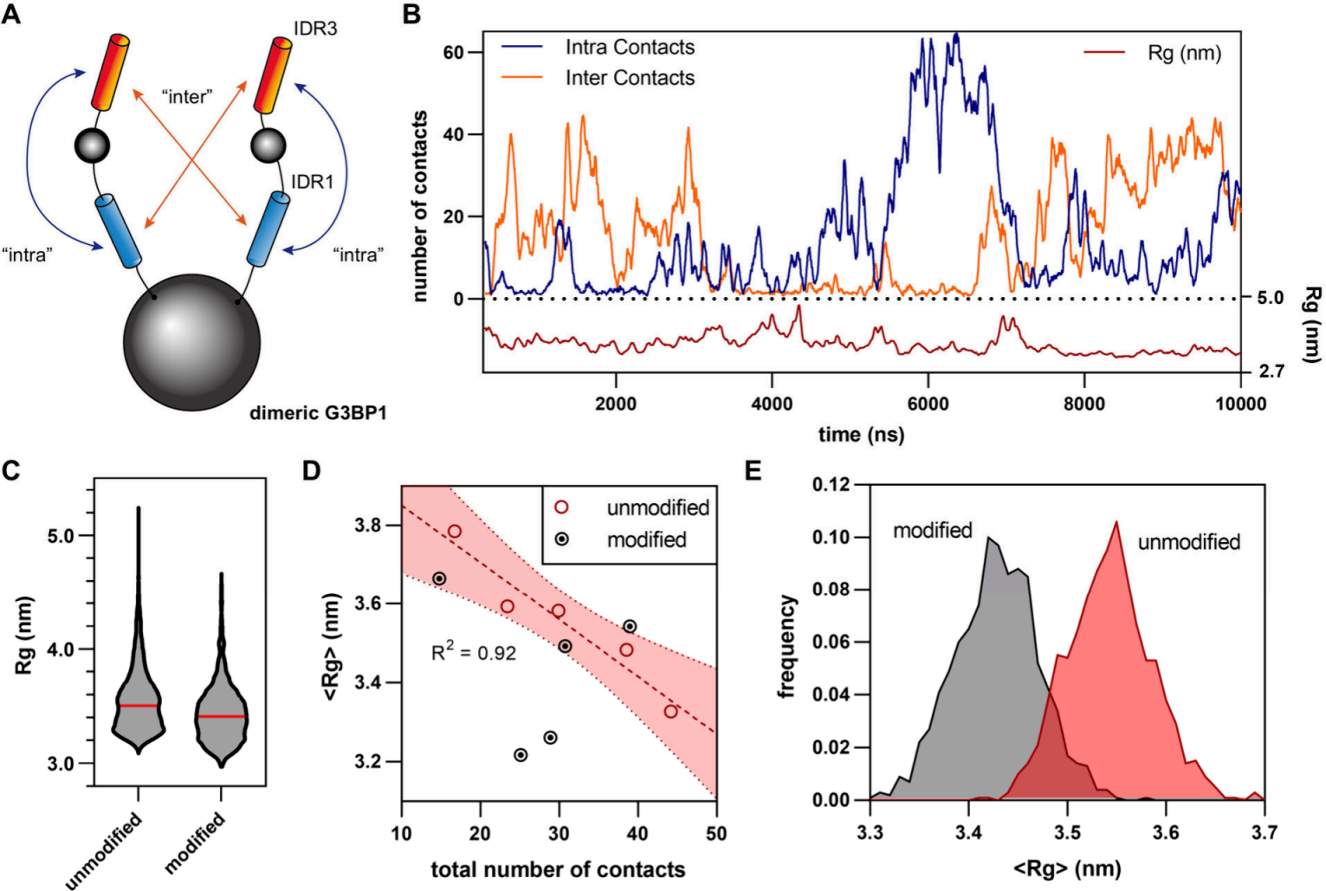
Coarse-grained MD simulation of CAA-AA-modified dimeric G3BP1. (A) Number of bead-to-bead contacts between IDR1 and IDR3 is introduced as an observable to complement radius of gyration (Rg) in characterizing IDR conformation dynamics. Conceptual schematic, not to scale. (B) 10-μs MD simulation of unmodified dimeric G3BP1. (C) Distribution of Rg values over 10-μs simulations for unmodified and CAA-AA-modified dimeric G3BP1. Red line, arithmetic mean (⟨Rg⟩). (D) Scatter plot of simulation observables following block averaging with a 2-μs block size. In unmodified dimeric G3BP1, ⟨Rg⟩ is inversely correlated with the total number of contacts. Dashed line, linear regression; shaded red area, 95% confidence region. (E) ⟨Rg⟩ distribution from 1000 time series simulated using linear autoregressive fit to MD data. Δ⟨Rg⟩ = 0.115 ± 0.0615 nm (s.d.).

In summary, we introduced a new charge-driven strategy of small-molecule modulation of biomolecular condensates with broad potential applicability to phase-separating proteins. We demonstrated the feasibility of this new strategy by developing covalent inhibitors of G3BP1-mediated SG formation via the proposed charge-driven MOA. We envision that the charge-driven strategy may apply to many phase-separating proteins whose phase behavior depends on charge-sensitive IDR dynamics. Following this approach, we are actively exploring opportunities to further rationalize the design of small-molecule modulators with potential for therapeutic utility.

## Supplementary Material



## Notes

Figures S1–S11, Schemes S1–S2, Materials, Methods, Synthesis protocols and characterization. (PDF)

## References

[1] Mitrea D. M., Mittasch M., Gomes B. F., Klein I. A., Murcko M. A. (2022). Modulating
biomolecular condensates: a novel approach to drug discovery. Nat. Rev. Drug Discov.

[2] Silva J. L. (2023). Targeting Biomolecular Condensation and Protein
Aggregation against
Cancer. Chem. Rev..

[3] Han T. W., Portz B., Young R. A., Boija A., Klein I. A. (2024). RNA and
condensates: Disease implications and therapeutic opportunities. Cell Chem. Biol..

[4] Brangwynne C. P. (2009). Germline P Granules
Are Liquid Droplets That Localize by Controlled
Dissolution/Condensation. Science.

[5] Brangwynne C. P., Mitchison T. J., Hyman A. A. (2011). Active liquid-like behavior of nucleoli
determines their size and shape in Xenopus laevis oocytes. Proc. Natl. Acad. Sci. U. S. A..

[6] Li P. (2012). Phase transitions in
the assembly of multivalent signalling proteins. Nature.

[7] Alberti S., Hyman A. A. (2021). Biomolecular condensates
at the nexus of cellular stress,
protein aggregation disease and ageing. Nat.
Rev. Mol. Cell Biol..

[8] Boija A., Klein I. A., Young R. A. (2021). Biomolecular Condensates
and Cancer. Cancer Cell.

[9] Shin Y., Brangwynne C. P. (2017). Liquid
phase condensation in cell physiology and disease. Science.

[10] Banani S. F., Lee H. O., Hyman A. A., Rosen M. K. (2017). Biomolecular condensates:
organizers of cellular biochemistry. Nat. Rev.
Mol. Cell Biol..

[11] Banani S. F. (2022). Genetic variation associated with condensate dysregulation in disease. Dev Cell.

[12] Patel A. (2022). Principles and functions
of condensate modifying drugs. Front Mol. Biosci.

[13] Lu B. (2021). Pharmacological Inhibition
of Core Regulatory Circuitry Liquid-liquid
Phase Separation Suppresses Metastasis and Chemoresistance in Osteosarcoma. Adv. Sci. (Weinh).

[14] Heberle A. M. (2019). The PI3K and MAPK/p38 pathways control stress granule assembly in
a hierarchical manner. Life Sci. Alliance.

[15] Fang M. Y. (2019). Small-Molecule Modulation
of TDP-43 Recruitment to Stress Granules
Prevents Persistent TDP-43 Accumulation in ALS/FTD. Neuron.

[16] Brunetti L. (2018). Mutant NPM1Maintains
the Leukemic State through HOX Expression. Cancer
Cell.

[17] Roden C., Gladfelter A. S. (2021). RNA contributions to the form and
function of biomolecular
condensates. Nat. Rev. Mol. Cell Biol..

[18] Biesaga M., Frigole-Vivas M., Salvatella X. (2021). Intrinsically disordered proteins
and biomolecular condensates as drug targets. Curr. Opin Chem. Biol..

[19] Schreiber S. L. (2019). A Chemical
Biology View of Bioactive Small Molecules and a Binder-Based Approach
to Connect Biology to Precision Medicines. Isr
J. Chem..

[20] Plenge R. M., Scolnick E. M., Altshuler D. (2013). Validating therapeutic targets through
human genetics. Nat. Rev. Drug Discov.

[21] Ochoa D. (2022). Human genetics evidence
supports two-thirds of the 2021 FDA-approved
drugs. Nat. Rev. Drug Discov.

[22] Dong T. (2025). G3BP1/2-Targeting PROTAC Disrupts Stress Granules Dependent
ATF4Migracytosis
as Cancer Therapy. J. Am. Chem. Soc..

[23] Nakamura T. (2023). Phase separation of
FSP1 promotes ferroptosis. Nature.

[24] Freibaum B. D. (2024). Identification of small
molecule inhibitors of G3BP-driven stress
granule formation. J. Cell Biol..

[25] Qi W. (2008). NSC348884, a nucleophosmin
inhibitor disrupts oligomer formation
and induces apoptosis in human cancer cells. Oncogene.

[26] Risso-Ballester J. (2021). A condensate-hardening drug blocks RSV replication in vivo. Nature.

[27] Zhu G. (2020). Phase Separation of Disease-Associated SHP2Mutants
Underlies MAPK
Hyperactivation. Cell.

[28] Li S., Wang Y., Lai L. (2023). Small molecules
in regulating protein
phase separation. Acta Biochim Biophys Sin (Shanghai).

[29] Bertoldo J. B., Muller S., Huttelmaier S. (2023). RNA-binding
proteins in cancer drug
discovery. Drug Discov Today.

[30] Campanile M., Kurtul E. D., Dec R., Mobitz S., Del Vecchio P., Petraccone L., Tatzelt J., Oliva R., Winter R. (2024). Morphological Transformations of SARS-CoV-2 Nucleocapsid
Protein
Biocondensates Mediated by Antimicrobial Peptides. Chem.—Eur. J..

[31] Mayer G. (2020). Targeting an Interaction Between Two Disordered Domains by Using
a Designed Peptide. Chem.—Eur. J..

[32] Biesaga M., Frigole-Vivas M., Salvatella X. (2021). Intrinsically disordered proteins
and biomolecular condensates as drug targets. Curr. Opin Chem. Biol..

[33] Wang H., Xiong R., Lai L. (2023). Rational drug design
targeting intrinsically
disordered proteins. WIREs Computational Molecular
Science.

[34] Martin E.
W. (2021). Interplay
of folded domains and the disordered low-complexity domain
in mediating hnRNPA1 phase separation. Nucleic
Acids Res..

[35] Taneja I., Holehouse A. S. (2021). Folded domain charge properties influence the conformational
behavior of disordered tails. Curr. Res. Struct
Biol..

[36] Kim J., Qin S., Zhou H. X., Rosen M. K. (2024). Surface Charge Can. Modulate Phase
Separation of Multidomain Proteins. J. Am. Chem.
Soc..

[37] Hess N., Joseph J. A. (2025). Structured protein domains enter the spotlight: modulators
of biomolecular condensate form and function. Trends Biochem. Sci..

[38] Yang P. (2020). G3BP1 Is a Tunable Switch that Triggers Phase Separation
to Assemble
Stress Granules. Cell.

[39] Bernardim B. (2016). Stoichiometric and irreversible
cysteine-selective protein modification
using carbonylacrylic reagents. Nat. Commun..

[40] Bernardim B. (2019). Efficient and irreversible
antibody-cysteine bioconjugation using
carbonylacrylic reagents. Nat. Protoc.

[41] Ciancone A. M. (2023). Global Discovery of
Covalent Modulators of Ribonucleoprotein Granules. J. Am. Chem. Soc..

[42] Julio A. R. (2025). Delineating cysteine-reactive compound modulation of cellular proteostasis
processes. Nat. Chem. Biol..

[43] Uechi H. (2025). Small-molecule dissolution
of stress granules by redox modulation
benefits ALS models. Nat. Chem. Biol..

[44] Chen L., Liu B. (2017). Relationships between Stress Granules, Oxidative Stress, and Neurodegenerative
Diseases. Oxid Med. Cell Longev.

[45] Yang P. G. (2020). G3BP1 Is a Tunable Switch
that Triggers Phase Separation to Assemble
Stress Granules. Cell.

[46] Bhardwaj G. (2016). Accurate de novo design
of hyperstable constrained peptides. Nature.

[47] Fleishman S. J. (2011). RosettaScripts: a scripting
language interface to the Rosetta macromolecular
modeling suite. PLoS One.

[48] Kroon P. C., Barnoud F. G. J, van
Tilburg M., Souza P. C. T., Wassenaar T. A., Marrink S. J. (2022). Martinize2 and Vermouth: Unified Framework for Topology
Generation. arXiv:2212.01191.

[49] Souza P. C. T. (2021). Martini 3: a general purpose force field for
coarse-grained
molecular dynamics. Nat. Methods.

