# Supplementary material for: A Charge-Driven Strategy for Covalent Modification and Modulation of Biomolecular Condensates
Source: J Am Chem Soc. 2025 Aug 2;147(32):28558–63. doi: 10.1021/jacs.5c06625 (PMC12356531; doi:10.1021/jacs.5c06625)
Supplement: Supplementary file 1 [file ja5c06625_si_001.pdf]

Supporting Information

A Charge-Driven Strategy for Covalent Modification and Modulation of Biomolecular Condensates

Zhewei Chen[1], Lu Liu[1], Jerome Cattin[2], Tuomas P. J. Knowles[1,3], Gonçalo J. L. Bernardes[1,4]*

[1]Yusuf Hamied Department of Chemistry, University of Cambridge, Cambridge CB2 1EW, U.K.

[2]Transition Bio, Ltd., Cambridge CB2 8DU, U.K.

[3]Cavendish Laboratory, Department of Physics, University of Cambridge, Cambridge CB3 0HE, U.K.

[4]Translational Chemical Biology Group, Spanish National Cancer Research Centre (CNIO), C/ Melchor Fernández Almagro, 3., 28029 Madrid, Spain.

Contents

Supporting Figures and Schemes 2
Biology Reagents and Kits 12
Experimental Methods 14
Organic Synthesis 19
NMR Spectra 29
References 55

Supporting Figures and Schemes

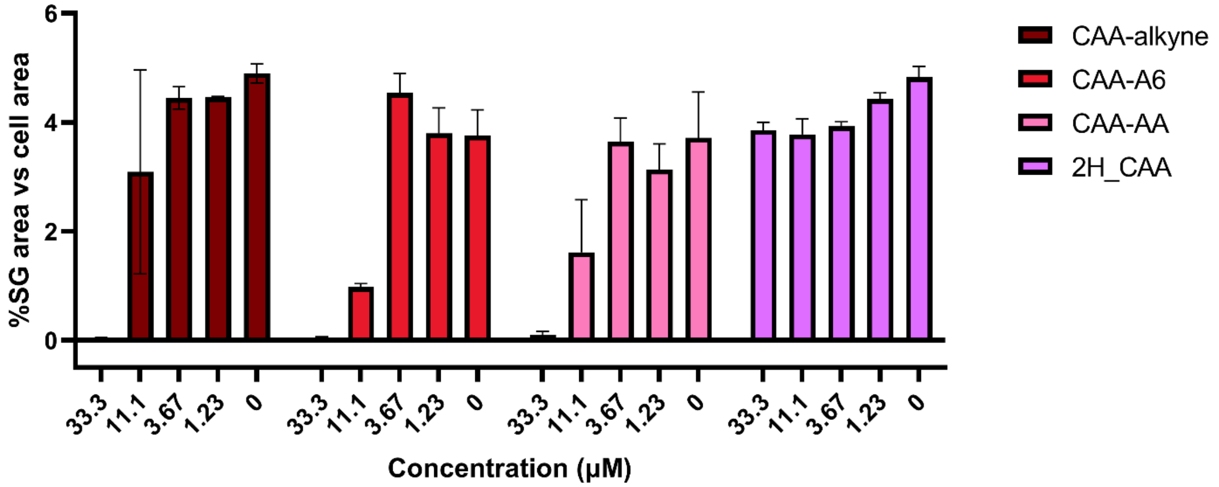

Figure S1. Dose-dependent inhibition of G3BP1-mediated stress granule (SG) formation. SG formation was assessed in HeLa cells expressing G3BP1-mScarlet using fluorescence microscopy. Quantification was performed algorithmically with CellPathFinder based on share of stress granule area versus total cell area. Data are presented as mean ± standard deviation.

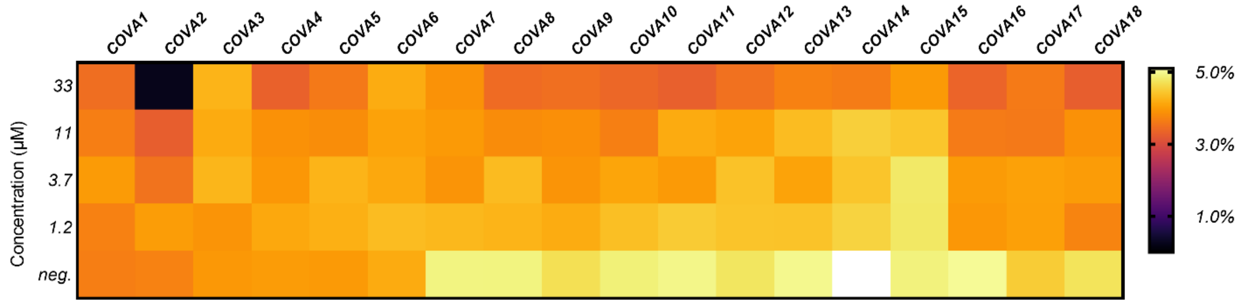

Figure S2. Generic electrophilic covalent probes do not inhibit G3BP1-mediated stress granule formation. A panel of 18 generic electrophilic covalent probes was screened using the fluorescence microscopy assay in HeLa cells expressing G3BP1-mScarlet. Among the compounds tested, only COVA2—an electrophile with cysteine reactivity comparable to CAA—significantly inhibited stress granule formation.

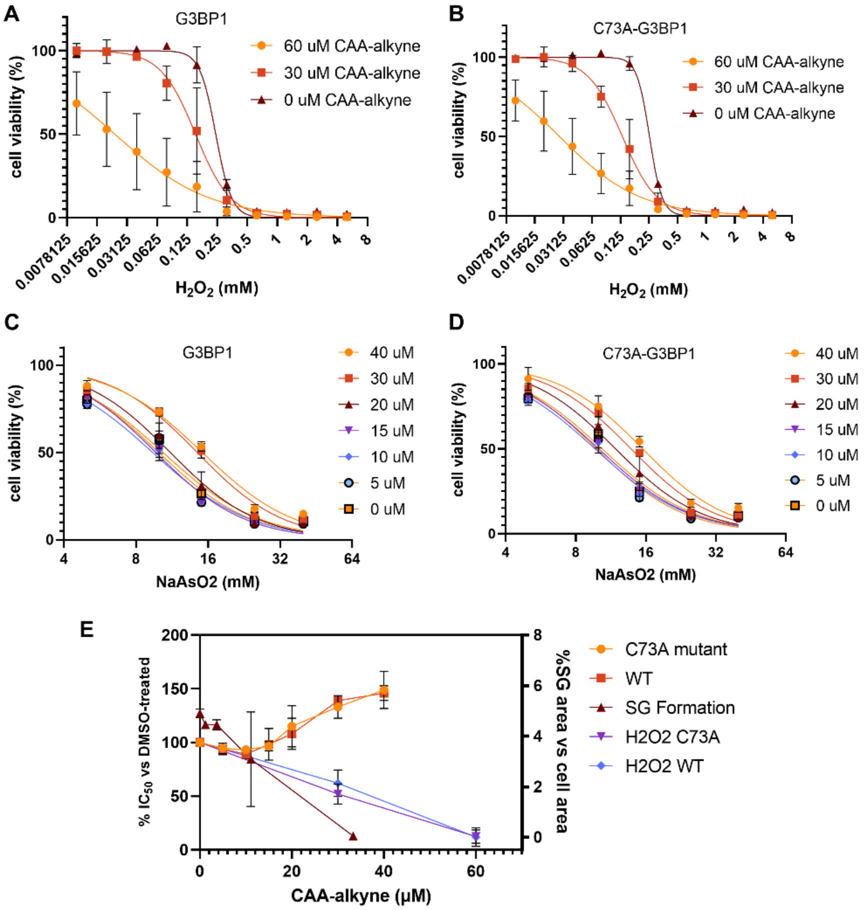

Figure S3. G3BP1 C73A mutation does not alter CAA-alkyne-induced H_2O_2 sensitization or $NaAsO_2$ desensitization in HeLa cells. (A-B) H_2O_2 sensitization assays in HeLa expressing wildtype or C73A-mutant G3BP1. *N* = 3. (C-D) $NaAsO_2$ desensitization assays in HeLa expressing wildtype or C73A-mutant G3BP1. *N* = 4. E. Summary alignment of dose-dependent trends in CAA-alkyne-induced stress sensitization/desensitization and inhibition of stress granule formation.

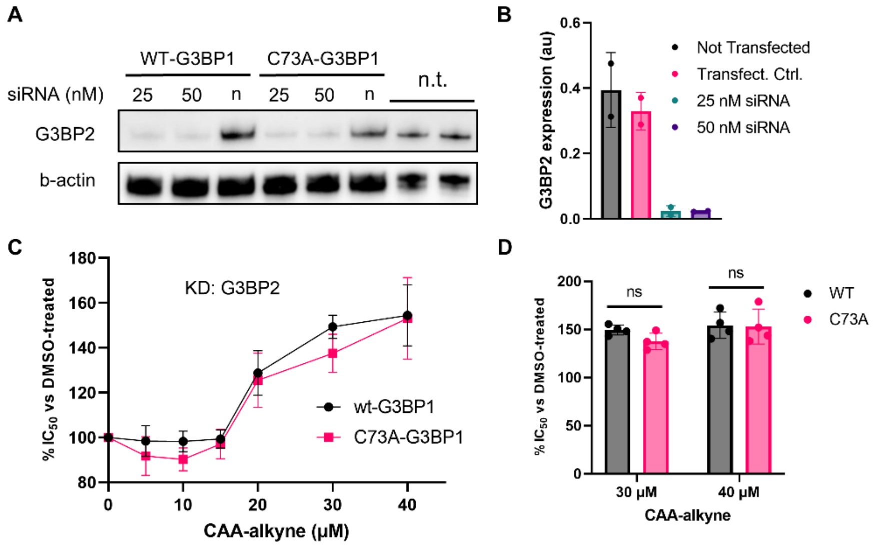

Figure S4. RNAi knockdown (KD) of G3BP2 does not alter the C73A mutation's effect on stress desensitization in HeLa cells treated with CAA-alkyne. A. Western blot analysis of mutant HeLa cells 54 hours after transfection with RNAi reagents. *n* = non-targeting control; *n.t.* = not transfected. B. G3BP2 expression was reduced by ~94% 54 hours after transfection with 25 nM siRNA reagents. This condition was used for stress sensitization assays with G3BP2 KD. C. Stress desensitization assays with wildtype and C73A-mutant G3BP1 HeLa cells were performed in parallel across four passages under $NaAsO_2$ stress. D. Paired *t*-test analysis (*n* = 4) showed that the C73A mutation did not significantly affect the desensitization response to CAA-alkyne treatment in the context of G3BP2 knockdown.

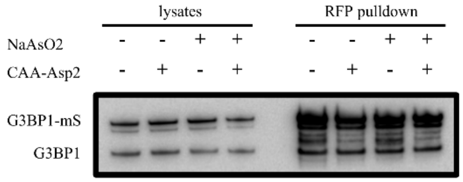

Figure S5. RFP pulldown experiments indicate that CAA-probe treatment does not disrupt G3BP1 dimerization. Western blot analysis of HeLa cells expressing G3BP1-mScarlet showed that both endogenous G3BP1 and G3BP1-mScarlet were co-purified using RFP-Trap following treatment with 75 μM CAA-Asp2 for 2 hours. Subsequent stress induction with 250 μM $NaAsO_2$ for 45 minutes did not alter the pulldown pattern, suggesting that G3BP1 dimerization is maintained under these conditions.

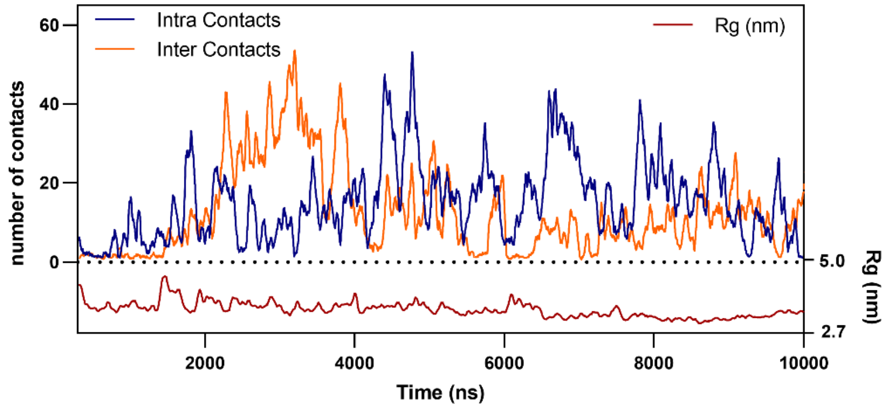

Figure S6. 10-μs MD simulation of CAA-AA-modified dimeric G3BP1. 25-step running averages of the observables plotted.

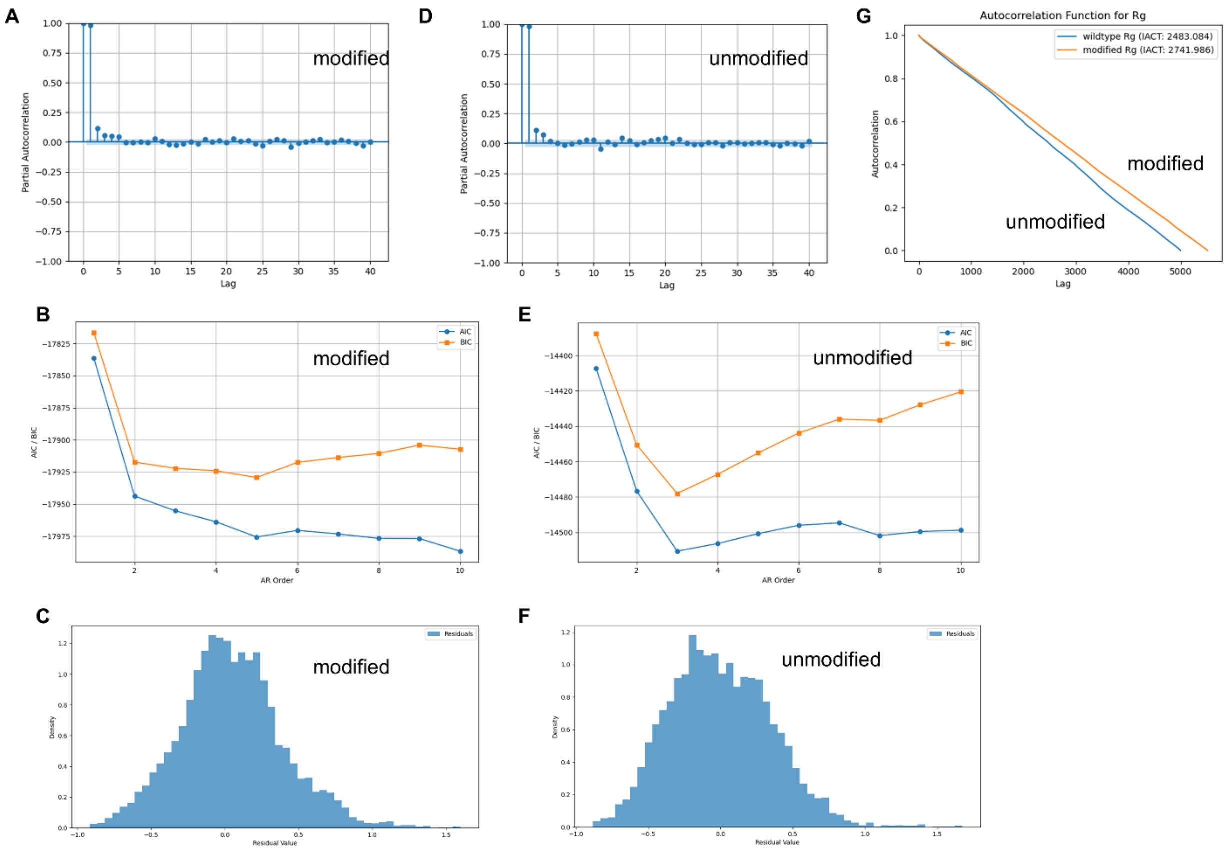

Figure S7. MD data analysis with autoregressive modeling. (A,D) Partial autocorrelation functions (PACFs) of Rg time series show a clear decay beyond lag 3, indicating that linear autoregressive modeling is appropriate. (B,E) Model order selection based on Akaike (AIC) and Bayesian (BIC) information criteria supports the use of a third-order autoregressive model (AR(3)). (C,F) Residuals from AR(3) model fitting are normally distributed around zero, indicating good model fit. G. The Rg time series is highly autocorrelated, with integrated autocorrelation times estimated between 2–3 μs.

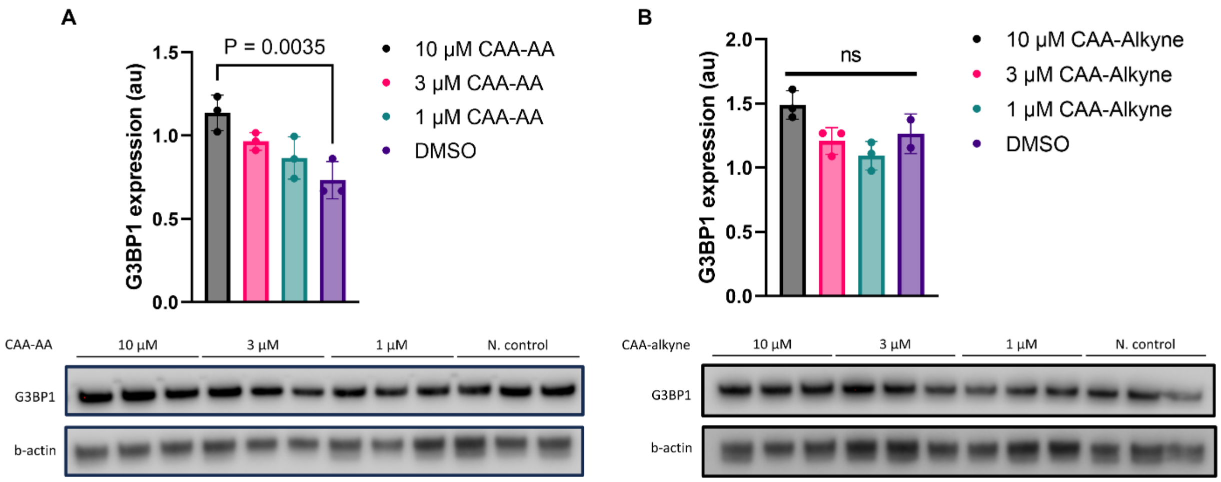

Figure S8. Western blot quantification of G3BP1 expression level in HeLa cells treated with CAA-AA or CAA-alkyne for 24 hours. Protein expression levels were normalized to β-actin and are shown relative to DMSO-treated control. Treatment with CAA-AA resulted in a ~55% increase in G3BP1 expression compared to control, while CAA-alkyne had no statistically significant effect. Statistical analysis was performed using ordinary one-way ANOVA (n=3).

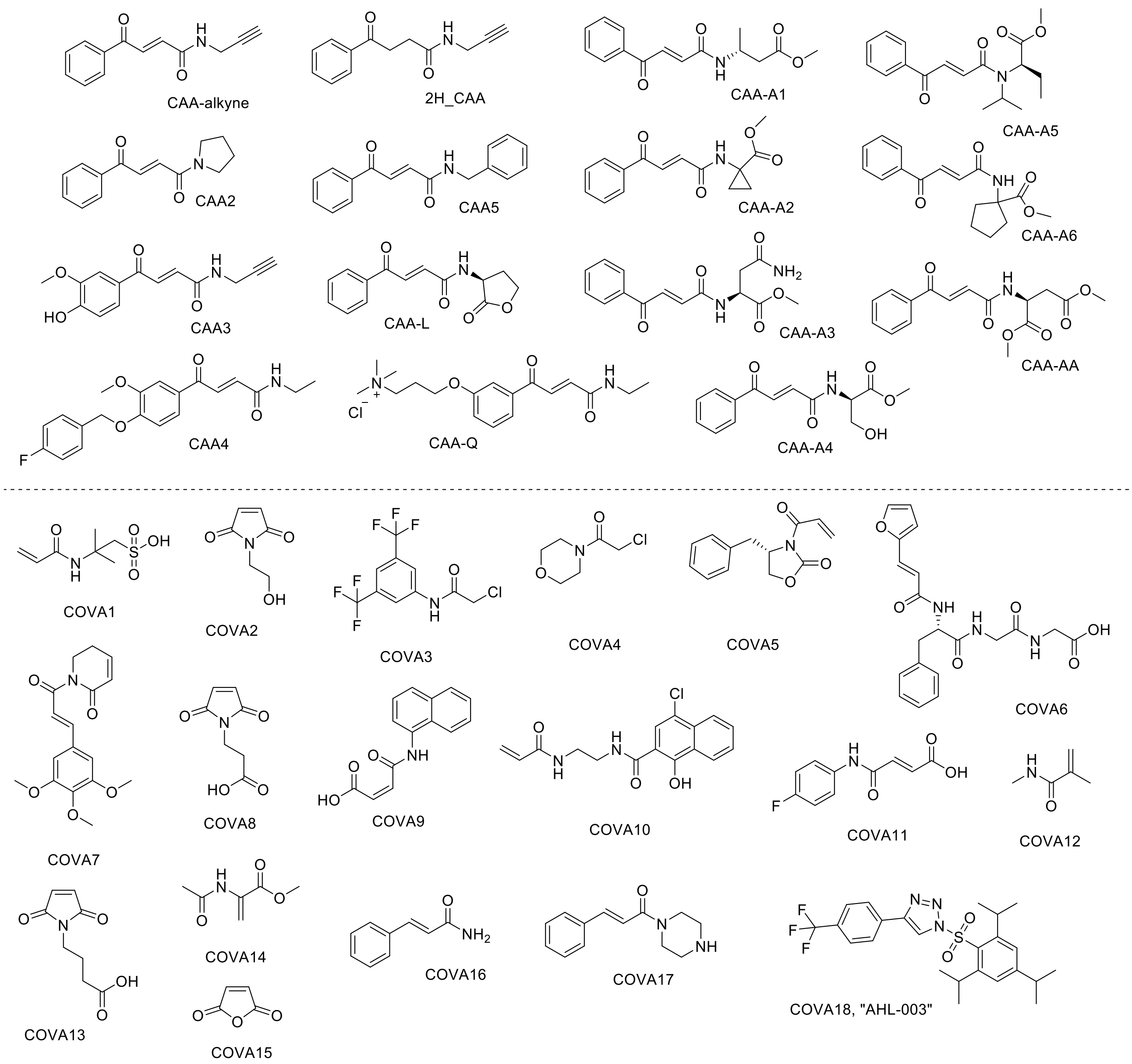

Figure S9. Structures of chemical probes screened in fluorescence microscopy experiments. The derivatives of CAA and COVA18 were synthesized. COVA1 to COVA17 were obtained from commercial vendors.

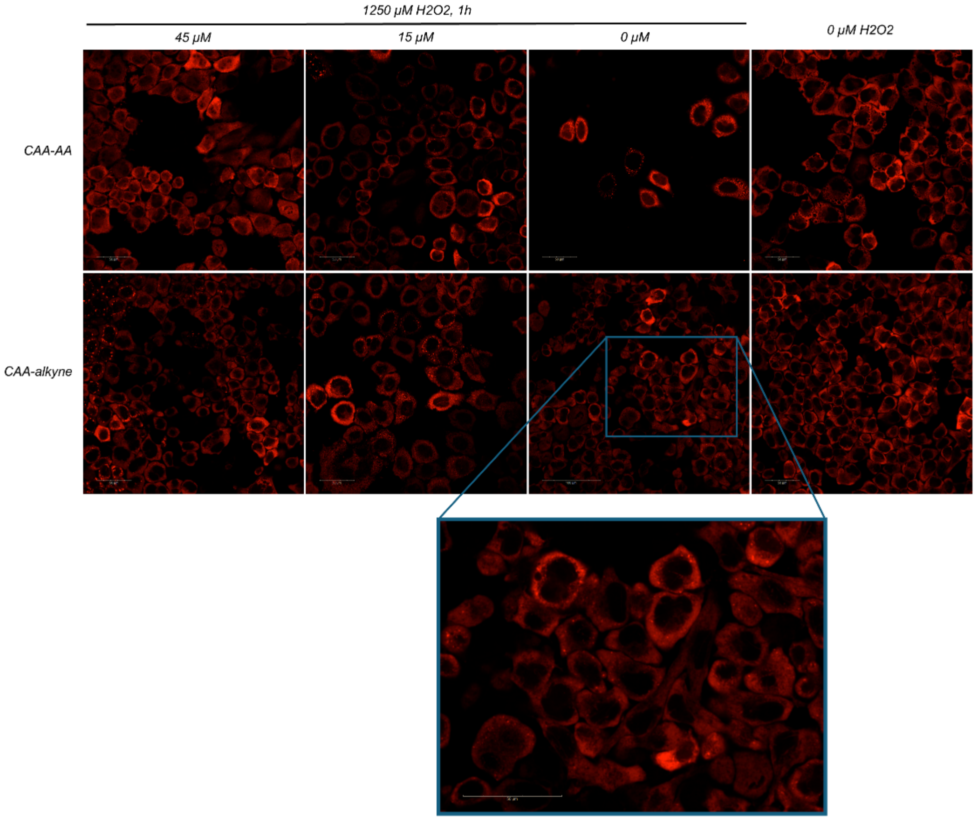

Figure S10. Fluorescence microscopy images showing inhibition of SGs induced by H_2O_2. HeLa cells expressing mScarlet_G3BP1 were treated with the CAA-derived chemical probes for 2 h before induction of SGs by H_2O_2. In these HeLa cells, SG formed in response to H_2O_2 are smaller than those that form in response to $NaAsO_2$. CAA-AA partially inhibits the formation of SGs at 15 μM and completely inhibits the formation of SGs at 45 μM. CAA-alkyne partially inhibits the formation of SGs at 45 μM.

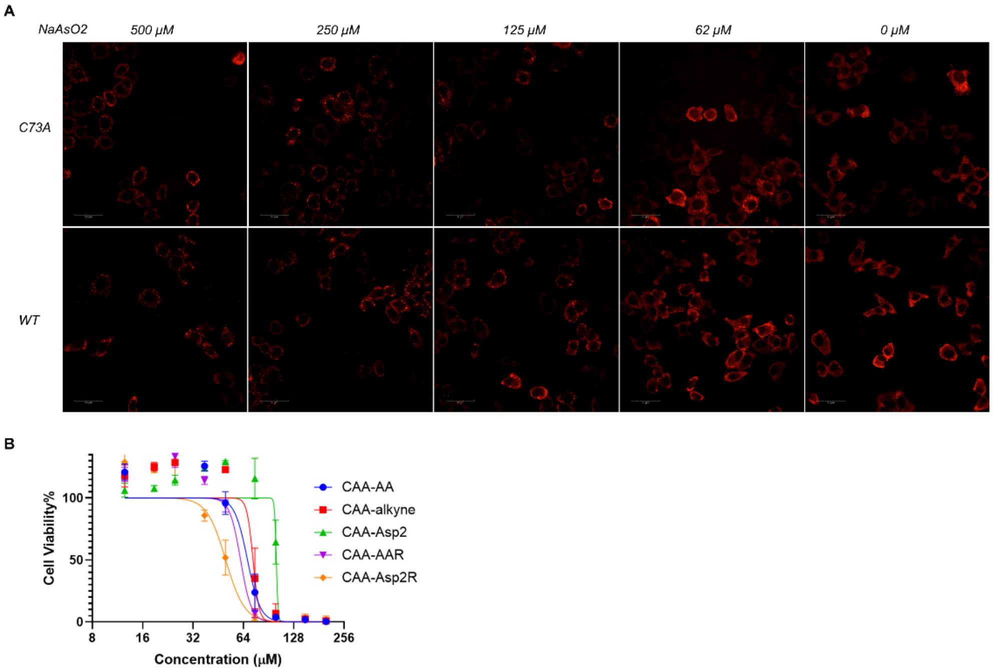

Figure S11. Control experiments. A. SGs were induced by treatment with various concentrations of $NaAsO_2$ for 45 min. HeLa cells expressing C73A-mutated G3BP1 and C73-G3BP1 respond similarly to $NaAsO_2$ treatment. B. Cell viability assays (cell titer glo) of the CAA-derived chemical probes used in mechanistic studies in HeLa. The cells were treated with the chemical probes at indicated concentrations for 4 h and assayed for cell viability. The CAA-derived chemical probes do not affect HeLa cell viability at concentrations relevant for SG inhibition, except for CAA-Asp2R, which induced a 15% decrease in cell viability at 37.5 µM. All other derivatives had a minimal effect (<10%) on cell viability at 50 µM. In particular, CAA-Asp_2 had no effect on cell viability at 75 µM and only showed toxicity at a very high concentration of 100 µM.

Scheme S1. Rg AR(3) fit report for MD simulation of CAA-AA-modified dimeric G3BP1.

```
                    AutoReg Model Results
==============================================================================
=========
Dep. Variable:              value   No. Observations:              5513
Model:                AutoReg(3)   Log Likelihood             8982.584
Method:             Conditional MLE   S.D. of innovations            0.047
Date:            Tue, 25 Mar 2025   AIC                       -17955.168
Time:                    04:59:09   BIC                    -17922.096
Sample:                         3   HQIC                   -17943.634
                         5513
==============================================================================
=========
              coef    std err          z      P>|z|      [0.025      0.975]
------------------------------------------------------------------------------
const          0.0544      0.009      6.125      0.000      0.037      0.072
value.L1       0.8482      0.013     63.061      0.000      0.822      0.875
value.L2       0.0795      0.018      4.515      0.000      0.045      0.114
value.L3       0.0565      0.013      4.211      0.000      0.030      0.083
                         Roots
==============================================================================
========
              Real         Imaginary          Modulus         Frequency
-----------------------------------------------------------------------------
AR.1           1.0134           -0.0000j           1.0134           -0.0000
AR.2          -1.2098           -3.9998j           4.1788           -0.2967
AR.3          -1.2098           +3.9998j           4.1788            0.2967
```

Scheme S2. Rg AR(3) fit report for MD simulation of unmodified dimeric G3BP1.

```
AR(3) Fit Summary for unmodified:
                   AutoReg Model Results
==============================================================================
Dep. Variable:              value   No. Observations:              4998
Model:                 AutoReg(3)   Log Likelihood             7260.312
Method:           Conditional MLE   S.D. of innovations           0.057
Date:            Tue, 25 Mar 2025   AIC                      -14510.624
Time:                    04:59:03   BIC                      -14478.043
Sample:                         3   HQIC                     -14499.204
                             4998
==============================================================================
                 coef    std err          z      P>|z|      [0.025      0.975]
------------------------------------------------------------------------------
const          0.0622      0.010      6.144      0.000       0.042       0.082
value.L1       0.8537      0.014     60.579      0.000       0.826       0.881
value.L2       0.0493      0.019      2.660      0.008       0.013       0.086
value.L3       0.0794      0.014      5.637      0.000       0.052       0.107
                                    Roots
=============================================================================
                  Real          Imaginary           Modulus         Frequency
-----------------------------------------------------------------------------
AR.1            1.0147           -0.0000j            1.0147           -0.0000
AR.2           -0.8182           -3.4275j            3.5238           -0.2873
AR.3           -0.8182           +3.4275j            3.5238            0.2873
-----------------------------------------------------------------------------
```

Biology Reagents and Kits

Name and Category	Supplier
Cloning	
One Shot® Mach1™-T1R Chemically Competent E. coli	Thermo Scientific C862003
pSpCas9(BB)-2A-Puro (PX459) V2.0	Addgene 62988
PiggyBac plasmids for G3BP1-mScarlet expression	Gifts from Dr. Jonnathan Nixon-Abell
FastDigest BbsI	Thermo Scientific FD1014
Tango Buffer	Thermo Scientific BY5
DNase I (RNase-free)	New England Biolabs M0303S
T4 polynucleotide kinase	New England Biolabs M0201S
PlasmidSafe ATP-dependent DNase	Epicentre E3101K
ATP, 10 mM	New England Biolabs P0756S
T4 DNA Ligase Reaction Buffer	New England Biolabs B0202S
Gibson Assembly Cloning Kit	New England Biolabs E5510S
FastDigest XhoI	Thermo Scientific FD0694
FastDigest XbaII	Thermo Scientific FD0684
VWR Gold Gel Extraction Kit	VWR 13-2500-01
QIAGEN Plasmid Plus Midi Prep	Qiagen 12941
DNA Clean and Concentrator™-5	Zymo D4003
Cell Work and Microscopy	
Lipofectamine 3000	Invitrogen 15282465
Puromycin dihydrochloride	Thermo Scientific 10781691
Hygromycin B	Stratech Scientific A2515-APE-200mg
Opti-MEM I reduced-serum medium	Life Technologies 11058-021
Halt Protease Inhibitor Cocktail, EDTA-Free (100X)	Thermo Scientific 10320015
RIPA Lysis and Extraction Buffer	Thermo Scientific 10017003
ON-TARGETplus Non-targeting Pool	Dharmacon D-001810-10-05
ON-TARGETplus Human G3BP2 (9908) siRNA - SMARTpool	Dharmacon L-015329-01-0005
DharmaFECT 1 Transfection Reagent	Dharmacon T-2001-01
Paraformaldehyde, 16% w/v	Thermo Scientific 11490570
Triton-X100	VWR PERCN9300260
DAPI Staining Solution	AbCam ab228549
poly-D-lysine coated PhenoPlate 384-well	Revvity 6007710
CellTiter Glo Luminescent Cell Viability Assay	Promega G7572
TrypLE Express	Gibco 12604
DMEM	Gibco 10566
FBS	Sigma-Aldrich F9665-500mL
Pen/Strep	Gibco 15140
Nuncleon Delta cell culture treated dishes	Thermo Scientific 150464
HeLa	ATCC CCL-2
Western Blot	
G3BP1 (E9G1M) XP® Rabbit mAb	CST 61559S
Anti-G3BP2 antibody	AbCam ab86135
Goat Anti-Rabbit IgG H&L (HRP)	Abcam ab205718
HRP-conjugated Streptavidin	Proteintech SA00001-0
β-Actin Antibody #4967	CST 4967S
NuPAGE™ 4-12% Bis-Tris Gel	Invitrogen NP0323
iBlot 2 PVDF Mini Stacks	Invitrogen IB24002
NuPAGE™ LDS Sample Buffer (4x)	Invitrogen NP0008
20x TBS Tween-20 Buffer	Thermo Scientific 28360
NuPAGE™ MES SDS Running Buffer (20x)	Invitrogen NP0002
Clarity Western ECL Substrate	Bio-RAD 170-5060
Pierce™ BCA Protein Assay Kits	Thermo Scientific 23225
SeeBlue™ Plus2 Pre-stained Protein Standard	Invitrogen LC5925

Others	
Recombinant Human G3BP1 His Protein	Bio-Techne, NBP1-50925-50ug
Biotin-PEG3-azide	Lumiprobe F3730
Zeba™ spin desalting columns	Thermo Scientific 89890
Pierce™ Streptavidin magnetic beads	Thermo Scientific ZF287022
Pierce™ BS3	Thermo Scientific A39266
RFP-Trap magnetic agarose kit	Chromotek rtmak
Oligo Sequences	*Supplier and Application*
CACCGGGAGAAGCCTAGTCCCCTGC	IDT, G3BP1 KO Forward-1
AAACGCAGGGGACTAGGCTTCTCCC	IDT, G3BP1 KO Reverse-1
CACCGAAGCCTAGTCCCCTGCTGGT	IDT, G3BP1 KO Forward-2
AAACACCAGCAGGGGACTAGGCTTC	IDT, G3BP1 KO Reverse-2
UGAAUAAAGCUCCGGAAUA	Dharmacon, siRNA J-015329-09
GAAUUUAAGUCUGGGACGA	Dharmacon, siRNA J-015329-10
ACAACGACCUAGAGAACGA	Dharmacon, siRNA J-015329-11
GCGAUGGUCUUGACUAUUA	Dharmacon, siRNA J-015329-12
GGTTCGGCTTCTGGCGTGTGACCGGCGGCTCTAGAGCCTCTGCTAACCATG	Life Technologies, PiggyBac Forward-1
GCATCAACATGGCGAATCTTGGTGTGTGCGTTGGTGAAGTTTTGTGACATCAC	Life Technologies, PiggyBac Reverse-1
GTGATGTCACAAAACTTCACCAACGCACACACCAAGATTCGCCATG	Life Technologies, PiggyBac Forward-2
GGGGAGGGCCTCTCATTCCACCACCCAGCCCACCTCGA	Life Technologies, PiggyBac Reverse-2

Experimental Methods

CAA-alkyne click pulldown
HeLa cells were cultured in 10-cm dishes to ~80% confluency. Cells were then incubated for 1 hour at 37°C with either DMSO (0.25%), CAA-alkyne (50 μM), or 2H_CAA (50 μM). After treatment, cells were harvested with ice-cooled PBS and lysed using RIPA lysis buffer (Pierce™ RIPA buffer + 1× Halt™ protease inhibitor cocktail). Cell lysates were incubated with a freshly prepared click reaction mixture and shaken at 400 rpm for 1 hour at 37°C. The click reaction mixture consisted of 3 volumes of 0.1 mg/mL THPTA, 1 volume of 2.0 mg/mL $CuSO_4$, 1 volume of 2.0 mg/mL TCEP, and 1 volume of 1 mM Biotin-PEG3-azide (in DMSO; Lumiprobe, F3730). A portion of DMSO-treated lysate was reserved for later analysis. Following the click reaction, excess small molecules were removed using Zeba™ spin desalting columns (Thermo Scientific, 89890). Each sample was then incubated with 50 μL of Pierce™ streptavidin magnetic beads (Thermo Fisher Scientific, ZF287022) for 1 hour at 20°C with gentle agitation. For quality control, additional samples containing only streptavidin beads and samples combining lysate with beads were also prepared. After incubation, the supernatant was collected for downstream analysis. Beads were washed thoroughly with TBST, and bound proteins were eluted by incubating with SDS-PAGE sample buffer (Invitrogen, NP0008) supplemented with 2 mM DTT at 95°C for 5 minutes. Supernatant (unbound fraction) and eluates (bound proteins) were analyzed by SDS-PAGE followed by Western blotting. The following antibodies were used: HRP-conjugated streptavidin (Proteintech, SA00001-0; 1:10,000), Goat Anti-Rabbit IgG H&L (HRP) (Abcam, ab205718; 1:5,000), and G3BP1 XP® Rabbit mAb (Cell Signaling Technology, 61559S; 1:1,000). The experiment was repeated once.

General mammalian cell culture
HeLa cells (acquired from ATCC), HeLa cell line expressing G3BP1-mScarlet (a gift from Dr. Jonnathan Nixon-Abell, constructed using PiggyBac method), and various mutant HeLa cells lines, were cultured in DMEM (Gibco 10566) supplemented with 10% fetal bovine serum (Sigma-Aldrich F9665) and 1x Pen/Strep (Gibco 15140) at 37°C and 5% CO_2. For general maintenance, the cells were split every 2-3 days as they reached ~70-80% confluency with TrypLE (Gibco 12604) into Nuncleon Delta cell culture treated dishes (ThermoFisher Scientific 150464). Passage numbers count either from receiving the cell line or from establishment of a mutant line.

Fluorescence microscopy quantification of SG formation
HeLa cells expressing G3BP1-mScarlet were cultured to approximately 70% confluency, then seeded at ~2,000 cells per well into poly-D-lysine-coated 384-well PhenoPlates (Revvity, 6007710). At the time of the experiment, cells had reached ~80–90% confluency. Cells were treated with various concentrations of chemical probes (final DMSO concentration: 1%) and incubated at 37°C for 2 hours. Stress granule formation was then induced by treating cells with 250 μM sodium arsenite ($NaAsO_2$) for 45 minutes at 37°C. Following stress induction, cells were fixed with 4% paraformaldehyde (PFA) for 10 minutes at 20°C, permeabilized with PBS containing 0.25% Triton X-100 for 10 minutes at 20°C, and stained with DAPI (1:2000 dilution; Abcam, ab228549; 10 mM stock) in PBS for 10 minutes at 20°C. Cells were washed three times with PBS before and after each fixation, permeabilization, and staining step. Fluorescence microscopy was performed using a high-content imaging system (Yokogawa CQ1 or Yokogawa CV8000). For each well, 4–6 image fields were acquired. Stress granule formation was quantified based on granule area relative to cell area using a custom analysis algorithm configured in CellPathFinder.

Hydrogen peroxide and sodium arsenite stress sensitization assays
HeLa cells and various mutant HeLa lines were cultured to ~70% confluency. Cell passage numbers ranged from 2 to 10. They were then seeded *accurately and uniformly* into 96-well plates 38–42 hours before the experiment, at densities expected to reach 80–90% confluency at the time of chemical probe dosing. Cells were treated with varying concentrations of chemical probes (final DMSO concentration: 1%) and incubated at 37°C for 2 hours. After this initial treatment, cells were exposed to different concentrations of $NaAsO_2$ or H_2O_2 and incubated for an additional 2 hours at 37°C. Cell viability was then measured using CellTiter-Glo (Promega G7572), following the manufacturer's protocol. For head-to-head comparisons between mutant lines, the cell lines were constructed, selected, and passaged in parallel. Paired t-tests were used when comparing data from different passages cultured in parallel. Unpaired t-tests were used when analyzing multiple cultures of the same passage or when one experimental arm was repeated to increase statistical power. Statistical analyses were performed using Prism software. Before conducting stress sensitization assays, ensure that media additives do not interfere with the experiment.

CRISPR KO of G3BP1 in HeLa
Protocol described by Ran *et al.*[1] was adapted for cloning the editor plasmids and performing CRISPR KO. Guide RNA sequences used for knocking out G3BP1 has been reported by Kedersha *et al.*[2] Briefly, complementary spacer DNA oligonucleotides (IDT) were annealed and cloned into the pSpCas9(BB)-2A-Puro (PX459) V2.0 vector (Addgene plasmid #62988; a gift from Feng Zhang) via direct ligation. The ligation products were treated with PlasmidSafe DNase (Epicentre, E3103K) and transformed into *E. coli* One Shot® Mach1™-T1R chemically competent cells (Thermo Fisher Scientific, C862003). Plasmids with correct insertions, confirmed by Sanger sequencing, were produced and transfected into HeLa cells (ATCC) using Lipofectamine™ 3000 (Invitrogen, 15282465) according to the manufacturer's instructions.
At 24 hours post-transfection, the medium was replaced with fresh growth medium containing 2 µg/mL puromycin. Cells were selected under puromycin for 3 days, after which the selection was removed. Single-cell clone selection was performed over the following 3 weeks to isolate G3BP1 knockout HeLa clones. Knockout efficiency was confirmed by Western blot analysis of G3BP1 expression.

PiggyBac transposon KI of G3BP1 (and C73A mutant) expression to HeLa
The PiggyBac™ transposon and transposase[3] plasmids for overexpression of G3BP1-mScarlet were kindly provided by Dr. Jonnathan Nixon-Abell. A PiggyBac transposon plasmid encoding a C73A-mutated variant of G3BP1 was generated via Gibson assembly. The assembled plasmids were transformed into One Shot® Mach1™-T1R chemically competent *E. coli* (Thermo Fisher Scientific, C862003), and the resulting clones were verified by full-plasmid sequencing (Plasmidsaurus). In parallel, both wild-type and C73A-mutated PiggyBac transposon plasmids were transfected into HeLa cells at approximately 90% confluency using Lipofectamine™ 3000 (Invitrogen, 15282465), following the manufacturer's protocol. Approximately 40 hours post-transfection, cells were placed under selection with Hygromycin B (200 µg/mL) for 7 days to establish stable expression of the G3BP1 constructs.

RNAi knock-down of G3BP2 expression in HeLa
HeLa cells and mutant derivatives were transfected at approximately 70% confluency with 25 nM or 50 nM ON-TARGETplus SMARTpool siRNA targeting **G3BP2** (L-012099-00) using DharmaFECT1 transfection reagent, according to the manufacturer's protocol.
At 24 hours post-transfection, cells were seeded into 96-well plates for downstream assays and into separate dishes for knockdown efficiency assessment. Experiments using these cells were

conducted 72 hours after siRNA transfection. Western blot analysis performed ~54 hours post-transfection indicated ~94% knockdown efficiency with the 25 nM siRNA condition.

Western Blot quantification of protein expression level
For protein extraction, cultured cells were washed with ice-cold PBS, placed on ice for 5 minutes, and gently scraped into PBS. Cells were collected by centrifugation and resuspended in RIPA buffer supplemented with 1× Halt™ protease inhibitor cocktail (Thermo Scientific). Lysates were incubated on ice for 15 minutes and then centrifuged at 14,000 × *g* at 4°C for 20 minutes. Protein concentrations in the supernatant were quantified using the BCA Protein Assay (Thermo Scientific). Equal amounts of protein were resolved by SDS-PAGE using NuPAGE™ 4–12% Bis-Tris Gels (Invitrogen) in NuPAGE™ MES SDS Running Buffer at 180 V for 35 minutes. Proteins were transferred onto PVDF membranes using the iBlot™ 2 Dry Blotting System (Invitrogen). Membranes were blocked in 5% skim milk in TBST for 1 hour at 20°C and incubated with primary antibodies overnight at 4°C with gentle shaking. After washing three times with TBST, membranes were incubated with HRP-conjugated secondary antibodies for 1 hour at 20°C. Detection was performed using Clarity™ Western ECL Substrate (Bio-Rad, 170-5060).
The following primary antibodies were used:

- G3BP1: (E9G1M) XP® Rabbit mAb (Cell Signaling Technology, 61559S), 1:1000 dilution
- G3BP2: Rabbit polyclonal antibody (Abcam, ab86135), 1:2000 dilution
- β-actin: Rabbit polyclonal antibody (Cell Signaling Technology, 4967S), 1:1000 dilution

The secondary antibody used was HRP-conjugated goat anti-rabbit IgG (Abcam, ab205718) at 1:5000 dilution.

RFP-beads pull down assay
HeLa cells expressing G3BP1-mScarlet were treated with 75 μM small-molecule probes for 2 hours followed by treatment with 250 μM $NaAsO_2$ for 45 min. Cells were collected and lysed with co-IP buffer (PBS pH 7.4, 0.5% NP-40, 0.2% Triton-X100) supplemented with protease inhibitors cocktail on ice for 30 min. Cell lysates were then centrifuged at 12,000 rpm for 20 min at 4°C. The supernatant was collected and incubated with RFP-beads (Chromotek rtmak) at 4°C overnight. The protein-bound beads were then gently washed three times using the wash buffer (PBS pH 7.4, 0.05% NP-40) and subsequently subjected for anti-G3BP1 Western blotting analysis.

Protein dimerization assay using BS3 crosslinker
HeLa cells were treated with small molecule probes for 2 hours at various concentrations followed by treatment with 250 μM $NaAsO_2$ for 45 min. Cells were collected and lysed with lysis buffer (PBS pH 7.4, 0.2% Triton-X100) supplemented with protease inhibitors cocktail on ice for 30 min. Cell lysates were then centrifuged at 12,000 rpm for 20 min at 4°C. The supernatant was collected and quantified with BSA assay. Then the lysates were added by 2.5 mM BS3 ((bis(sulfosuccinimidyl)suberate)) probe (Thermo Scientific, A39266) for 30 min at room temperature and subsequently subjected for anti-G3BP1 and anti-Actin Western blotting analysis

Coarse-grained molecular dynamics simulations of dimeric G3BP1 proteins
To generate a structural model of dimeric G3BP1 modified by the CAA-AA acid for use as molecular dynamics simulation input, the loop (aa. 69-74) that contains Cys73 was remodeled with Rosetta using the Generalized Kinematic Closure (GenKIC).[4] The CAA-modified cysteine was parametrized (250 rotamers) as a non-canonical amino acid (NCAA) for Rosetta with the recently reported FakeRotLib tools.[5] A structural model of the unmodified dimeric G3BP1 was generated from a crystal structure of the dimerized NTF2 domain (pdb3q90, accessed from

PDB, deposited by Welin M et al, “to be published”) and loop regions not well defined in this structure was completed with AlphaFold predicted structure (AF-Q13283)[6]. Cys73 of this structure was replaced with the CAA-modified cysteine (CAA-Cys) as NCAAs. Based on this structure of the dimer, the CAA-modified loops of interest were remodeled with GenKIC (nstruct = 250) using a Monte Carlo protocol (10000 trials at temperature 1.0), the standard ref2015 scoring function (ref15sfxn) and a backbone-centric scoring function (bb_only). The best-scored dimer structure was used as input for downstream molecular dynamics simulations. Next, both the all-atom CAA-modified and unmodified dimeric G3BP1 structures were coarse-grained for molecular dynamics simulations with MARTINI3001 force field using GROMAC 2024.5. CAA-Cys was mapped to the MARTINI3001 force field as the scheme below shows and the bond parameters were calculated with Bartender, which performs quantum mechanics-based molecular dynamics simulations on the CAA-Cys structure.[7] With calculated bond parameters, the CAA-modified and unmodified dimeric G3BP1 were coarse-grained with the Martinize2 python script (-p backbone -ef 1000 -el 0.5 -eu 0.9 -ea 0 -ep 0).[8] Elastic network (EN) was applied to the folded domains of G3BP1 (aa. 11-133 and aa. 335-410). Additionally, to maintain the dimeric structure of G3BP1 through MD simulations, 43 rubber bands were added across the dimerization surface. After coarse-graining the structures, solvation and neutralization was performed with the Insane python script where a salt concentration of 150 mM NaCl was modeled.[9] Molecular dynamics simulations were then performed using parameters adapted from a previous study on FD-IDR interplay.[10] Briefly, minimization was performed for 0.3 ns with a 30 fs timestep using the Berendsen thermostat, the Berendsen barostat, and the Verlet cut-off scheme; equilibration was performed for 50 ns with a 20 fs timestep using the v-rescale thermostat at 310K, the Parrinello-Rahman barostat, and the Verlet cut-off scheme; molecular dynamics simulations were then performed with a timestep of 20 fs for the unmodified dimeric G3BP1 and 18 fs for the CAA-modified dimeric G3BP1 (reduced timestep due to numeric stability concerns) using the v-rescale thermostat at 310K, the Parrinello-Rahman barostat, and the Verlet cut-off scheme. Setup files, scripts, output data are available from the corresponding author upon reasonable request.

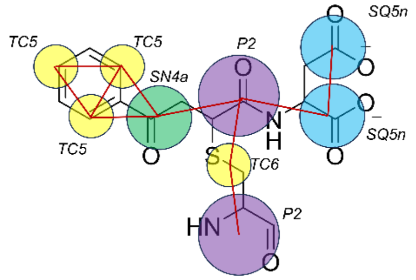

Analysis of molecular dynamics simulations

After completion of 10-microsecond MD simulations, the observables of radius of gyration (Rg) and number of contacts (ncontact) data were extracted with GROMAC tools of *gyrate* and *mindist* from simulation frames saved every 2 nanoseconds. Contacts between IDR1 (aa. 143-225) and IDR3 (aa. 411-466) are defined by a bead-to-bead distance of <0.6 nm; the -group flag was used to prevent over-counting. Extracted data was preprocessed by discarding the first 300 nanoseconds of simulations and transforming ncontact data into the *inter* and *intra* classes (contacts between G3BP1 monomers vs contacts within a G3BP1 monomer). 25-step running averages of the extracted data were plotted (Fig. 5B and Fig. S6). Autocorrelation of Rg was calculated with autocorrelation function to show that the dynamic behavior of dimeric G3BP1 is highly auto-correlated, with integrated autocorrelation times between 2 to 3 microseconds (Fig. S7). Thus, we reasoned that analysis of the observables using autoregression models is

necessary while the observables were also blocked with a block size of 2 microseconds for analysis. Suitability of linear autoregressive models for analyzing Rg was evaluated with partial autocorrelation function and the AIC/BIC criteria. Our evaluations showed that linear autoregressive model of order 3 (AR(3)) is suitable for our analysis (Fig. S7). Thus, Rg extracted from molecular dynamics simulations was modeled with linear AR(3) (Scheme S1-S2). Residues of AR(3) fitting distribute normally around 0 (Fig. S7). Coefficients of the AR(3) model were then used for quantitative comparison of the behaviors of wildtype and modified dimeric G3BP1. While the coefficients of the best-fit AR(3) models were not stationary, our tests showed that in the simulation time span, they are stable. Therefore, we evaluated the long-term Rg of wiltype and modified dimeric G3BP1 based on the AR(3) model with 5000-step simulated Rg time series (Fig. 5E). The simulation results showed that unmodified and modified dimeric G3BP1 generate different long-term Rg behaviors. Our model predicts a 0.115 ± 0.0615 nm decrease of dimeric G3BP1 Rg in the long run upon modification with the negatively charged CAA-AA probe, the effect is comparable to that conferred by a ~26% increase in IDR1-IDR3 interaction (based on linear fit of Rg vs ncontact for wildtype dimeric G3BP1). Data analysis was performed with Python. Scripts used for data analysis are available from the corresponding author upon reasonable request.

Organic Synthesis

Proton and carbon nuclear magnetic resonance (1H NMR and 13C NMR) spectra were acquired with Bruker Avance III HD (400 MHz) or Bruker Avance NEO Prodigy (400 MHz) or Bruker Avance III 500 (500 MHz) spectrometer at ambient temperature, using deuterated solvents as indicated. Chemical shifts (δ) are reported in parts per million (ppm) relative to the residual solvent, and coupling constants (J) are reported in hertz (Hz). Multiplicity is reported using combinations of the following abbreviations: s = singlet, d = doublet, t = triplet, m = multiplet/overlapping peaks, br = broad. Analysis of NMR spectra was performed using MestReNova software. High resolution mass spectra (HRMS) for small molecules were recorded on a Waters LCT Premier TOF spectrometer.

General protocol: *amide coupling (ethyl chloroformate)*, adapted from *J. Org. Chem.* 2011, 76, 680–683.[11] with modification. Refer to the synthesis of "CAA-alkyne" for an example.

General protocol: *ether synthesis from alkyl bromides and phenols*. In a dry 4 mL glass vial equipped with a magnetic stir bar, the alkyl bromide (0.030 mmol) and the phenol (0.030 mmol) were dissolved in anhydrous DMF (1.0 mL). Oven-dried K_2CO_3 (0.075 mmol) and 4 Å molecular sieves were added to solution, which was allowed to be stirred at 20 °C for 16 h. The solution was extracted (ethyl acetate, water), dried (brine, $MgSO_4$), concentrated in vacuo. The residue was dissolved in HPLC grade water/acetonitrile (1:1) and purified by HPLC (C8, 95% to 30% water/acetonitrile gradient, 0.1% TFA) to give the product.

General protocol: *synthesis of carbonylacrylic acid by aldol condensation under acidic conditions*.[12] Example implementation: 1-(4-hydroxy-3-methoxyphenyl)ethan-1-one (3.32 g, 20 mmol) and glyoxylic acid monohydrate (1.84 g, 20 mmol) were refluxed with glacial acetic acid (4.0 mL) for 20 h. The mixture was cooled down. After about 10 min, solid precipitated from the solution. The solid was separated by filtration and washed with glacial acetic acid, then recrystallized from ethyl acetate/hexanes to yield (E)-4-(4-hydroxy-3-methoxyphenyl)-4-oxobut-2-enoic acid (1.55 g, 6.98 mmol, 35%) as a brown crystalline solid.

General protocol: *small-scale carbonylacrylic amide synthesis with HATU*. Carbonylacrylic acid (17.6 mg, 0.100 mmol) and HATU (39.1 mg, 0.103 mmol) were suspended in anhydrous DCM (2 mL) and stirred at 20 °C. DIPEA (5 equiv.) was added to the suspension, followed by the amine substrate (~0.10 mmol, 1.0 equiv.). The solution was stirred at 20 °C for 16 to 24 h. The solution was diluted into saturated NH_4Cl (4 mL) and extracted with ethyl acetate (4 mL). The organic layer was dried and evaporated. The remaining residue was purified by HPLC to give the product.

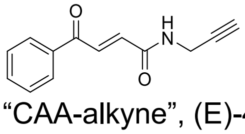

"CAA-alkyne", (E)-4-oxo-4-phenyl-N-(prop-2-yn-1-yl)but-2-enamide

To a cooled (-20°C using 1:3 NaCl/ice bath) solution of 4-oxo-4-phenylbutanoic acid (367 mg, 2.09 mmol) and N-methylmorpholine (NMM, 330 µL, 3.00 mmol) in THF (10 mL) was added $ClCO_2Et$ (194 µL, 2.04 mmol) under a stream of nitrogen and vigorous stirring. After 2 min, a suspension of propargyl amine hydrochloride (184 mg, 2.01 mmol) in DMF/THF (2/4 mL) was added to the reaction followed by more NMM (550 µL, 5.00 mmol). After 10 min, the reaction was removed from the ice bath and stirred at 20°C for 4 h. The volatile was removed under

reduced pressure and the residual material diluted into water (30 mL), extracted with ethyl acetate (25 mL, 3 times). The organic fraction was dried and concentrated under reduced pressure. The organic residue was purified by HPLC (C8, 5% to 75% acetonitrile/water, 0.1% TFA) to give (E)-4-oxo-4-phenyl-N-(prop-2-yn-1-yl)but-2-enamide (136 mg, 0.64 mmol, 32%) as a white solid. ^{1}H NMR (500 MHz, Acetone) δ 8.14 (s, 1H), 8.11 – 8.05 (m, 2H), 7.90 (d, *J* = 15.3 Hz, 1H), 7.74 – 7.67 (m, 1H), 7.64 – 7.57 (m, 2H), 7.07 (d, *J* = 15.3 Hz, 1H), 4.17 (dd, *J* = 5.5, 2.6 Hz, 2H), 2.74 (t, *J* = 2.6 Hz, 1H). Chemical Formula: C13H11NO2. $(M+H)^{+}$, m/z (calc) = 214.0863, found = 214.0856, diff = -3.19 ppm.

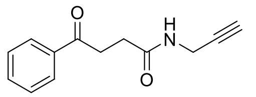

"2H_CAA", 4-oxo-4-phenyl-N-(prop-2-yn-1-yl)butanamide
At 20°C, to a stirred solution of 4-oxo-4-phenylbutanoic acid (178 mg, 1.00 mmol) and HATU (385 mg, 1.01 mmol) in DMF (4 mL) was added diisopropylethylamine (740 µL) followed by propargyl amine hydrochloride (151 mg, 1.66 mmol). After 16 h, the reaction mixture was diluted into ethyl acetate (50 mL) and washed sequentially with saturated aqueous ammonia chloride, saturated aqueous sodium bicarbonate, and saturated aqueous sodium chloride (25 mL each). The organic fraction was dried with $MgSO_4$ and concentrated under reduced pressure. The organic residue was then purified with flash column chromatography (silica, 35% ethyl acetate/hexanes) to give 4-oxo-4-phenyl-N-(prop-2-yn-1-yl)butanamide as a white solid (127 mg, 0.59 mmol, 59%). R_f = 0.30 in 50% ethyl acetate/hexanes. ^{1}H NMR (400 MHz, Acetone) δ 8.09 – 7.98 (m, 2H), 7.67 – 7.58 (m, 1H), 7.52 (ddd, *J* = 8.1, 6.5, 1.3 Hz, 2H), 4.02 (dd, *J* = 5.5, 2.6 Hz, 2H), 3.34 (t, *J* = 6.7 Hz, 2H), 2.71 – 2.59 (m, 3H). ^{13}C NMR (101 MHz, Acetone) δ 198.3, 171.2, 137.1, 132.9, 128.6, 127.9, 80.7, 71.1, 33.3, 29.2, 28.2. Chemical Formula: C13H13NO2. $(M+H)^{+}$, m/z (calc) = 216.1019, found = 216.1022, diff = 1.54 ppm.

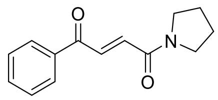

"CAA2", (E)-1-phenyl-4-(pyrrolidin-1-yl)but-2-ene-1,4-dione
General protocol *small scale carbonylacrylic amide synthesis with HATU* was followed for pyrrolidine (7.1 mg, 0.100 mmol) and (E)-4-oxo-4-phenylbut-2-enoic acid (17.6 mg, 0.100 mmol) to yield (E)-1-phenyl-4-(pyrrolidin-1-yl)but-2-ene-1,4-dione (7.5 mg, 0.033 mmol, 33%) as a white solid after purification with HPLC (C8 column, 5% to 80% acetonitrile/water, 0.1% TFA). ^{1}H NMR (400 MHz, DMSO) δ 8.08 – 8.00 (m, 2H), 7.79 (d, *J* = 15.2 Hz, 1H), 7.74 – 7.68 (m, 1H), 7.64 – 7.54 (m, 2H), 7.25 (d, *J* = 15.1 Hz, 1H), 3.63 (t, *J* = 6.8 Hz, 2H), 3.43 (t, *J* = 6.9 Hz, 2H), 1.98 – 1.77 (m, 4H). ^{13}C NMR (101 MHz, DMSO) δ 190.3, 162.5, 137.0, 134.8, 134.3, 133.3, 129.5, 129.1, 46.7, 46.4, 26.1, 24.3. Chemical Formula: C14H15NO2. $(M+H)^{+}$, m/z (calc) = 230.1176, found = 230.1177, diff = 0.64 ppm.

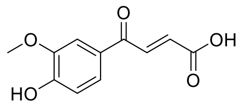

(E)-4-(4-hydroxy-3-methoxyphenyl)-4-oxobut-2-enoic acid
General protocol *synthesis of carbonylacrylic acid by aldol condensation under acidic conditions* was followed for 1-(4-hydroxy-3-methoxyphenyl)ethan-1-one (3.32 g, 20 mmol) and glyoxylic acid monohydrate (1.84 g, 20 mmol) to yield (E)-4-(4-hydroxy-3-methoxyphenyl)-4-oxobut-2-enoic acid (1.55 g, 6.98 mmol, 35%) as a brown crystalline solid (recrystallized from ethyl acetate/hexanes). ^{1}H NMR (400 MHz, DMSO) δ 13.03 (s, 1H), 10.31 (s, 1H), 7.91 (d, *J* = 15.5 Hz, 1H), 7.64 (dd, *J* = 8.3, 2.1 Hz, 1H), 7.52 (d, *J* = 2.1 Hz, 1H), 6.92 (d, *J* = 8.3 Hz, 1H), 6.65

(d, J = 15.4 Hz, 1H), 3.86 (s, 3H). ^{13}C NMR (101 MHz, DMSO) δ 187.4, 167.0, 153.3, 148.5, 136.7, 132.4, 128.6, 125.0, 115.6, 111.9, 56.1. Spectra match previous report.[12]

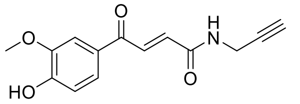

CAA3, (E)-4-(4-hydroxy-3-methoxyphenyl)-4-oxo-N-(prop-2-yn-1-yl)but-2-enamide
General protocol *amide coupling (ethyl chloroformate)* was followed for (E)-4-(4-hydroxy-3-methoxyphenyl)-4-oxobut-2-enoic acid (665 mg, 2.99 mmol) and propargylamine hydrochloride (273 mg, 2.98 mmol). After workup, the organic residue obtained after extraction and evaporation was unsuccessfully purified with flash column chromatography. A small sample was further purified with HPLC (C8 column, 5% to 80% acetonitrile/water, 0.1% TFA) to yield (E)-4-(4-hydroxy-3-methoxyphenyl)-4-oxo-N-(prop-2-yn-1-yl)but-2-enamide as a yellow solid. ^{1}H NMR (500 MHz, Acetone) δ 7.92 (d, J = 15.2 Hz, 1H), 7.71 (dd, J = 8.3, 2.0 Hz, 1H), 7.64 (d, J = 2.0 Hz, 1H), 7.04 (d, J = 15.3 Hz, 1H), 7.01 (d, J = 8.5 Hz, 1H), 4.16 (d, J = 2.6 Hz, 2H), 3.96 (s, 3H), 2.73 (t, J = 2.6 Hz, 1H). ^{13}C NMR (126 MHz, Acetone) δ 187.1, 163.4, 152.2, 147.8, 134.2, 132.9, 129.6, 124.1, 114.7, 111.1, 79.9, 71.5, 55.5. Chemical Formula: C14H13NO4. $(M+Na)^+$, m/z (calc) = 282.0737, found = 282.0745, diff = 2.81 ppm.

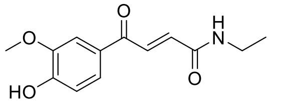

(E)-N-ethyl-4-(4-hydroxy-3-methoxyphenyl)-4-oxobut-2-enamide
General protocol *amide coupling (ethyl chloroformate)* was followed for (E)-4-(4-hydroxy-3-methoxyphenyl)-4-oxobut-2-enoic acid (222 mg, 1.00 mmol), albeit the cooling might not have been sufficient. After 30 min at 20°C, the reaction was considered completed by TLC. The organic residue obtained from workup was purified by flash column chromatography (silica gel, 4% MeOH/DCM) to yield (E)-N-ethyl-4-(4-hydroxy-3-methoxyphenyl)-4-oxobut-2-enamide (50 mg, 0.20 mmol, 20%) as a yellow solid. ^{1}H NMR (400 MHz, DMSO) δ 10.15 (s, 1H), 8.54 (t, J = 5.6 Hz, 1H), 7.78 (d, J = 15.2 Hz, 1H), 7.62 (dd, J = 8.3, 2.0 Hz, 1H), 7.51 (d, J = 2.1 Hz, 1H), 7.00 – 6.89 (m, 2H), 3.86 (s, 3H), 3.22 (qd, J = 7.2, 5.5 Hz, 2H), 1.09 (t, J = 7.3 Hz, 3H). ^{13}C NMR (101 MHz, DMSO) δ 187.7, 163.8, 153.2, 148.5, 136.0, 132.0, 128.9, 124.7, 115.6, 111.8, 56.1, 34.2, 15.0.

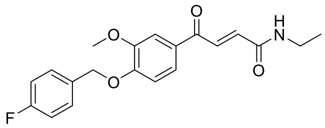

"CAA4", (E)-N-ethyl-4-(4-((4-fluorobenzyl)oxy)-3-methoxyphenyl)-4-oxobut-2-enamide
General protocol *ether synthesis from alkyl bromides and phenols* was followed for 1-(bromomethyl)-4-fluorobenzene (9.5 mg, 0.050 mmol) and (E)-N-ethyl-4-(4-hydroxy-3-methoxyphenyl)-4-oxobut-2-enamide (10.8 mg, 0.049 mmol) to yield (E)-N-ethyl-4-(3-((4-fluorobenzyl)oxy)phenyl)-4-oxobut-2-enamide (2.5 mg, 0.0076 mmol, 16%) as a white solid after purification with HPLC (C8 column, 5% to 80% acetonitrile/water, 0.1% TFA). ^{1}H NMR (400 MHz, DMSO) δ 8.59 (t, J = 5.6 Hz, 1H), 7.73 (d, J = 15.4 Hz, 1H), 7.63 (dt, J = 7.6, 1.2 Hz, 1H), 7.58 – 7.49 (m, 4H), 7.35 (ddd, J = 8.2, 2.7, 1.0 Hz, 1H), 7.29 – 7.17 (m, 2H), 6.96 (d, J = 15.3 Hz, 1H), 5.19 (s, 2H), 3.23 (qd, J = 7.2, 5.4 Hz, 2H), 1.09 (t, J = 7.2 Hz, 3H). ^{13}C NMR (101 MHz, DMSO) δ 190.1, 163.6, 159.1, 138.5, 137.2, 133.4, 132.1, 130.7, 130.5, 130.5, 122.0, 121.2, 115.9, 115.7, 114.3, 69.2, 34.3, 14.9. Chemical Formula: C20H20FNO4. $(M+Na)^+$, m/z (calc) = 380.1269, found = 380.1275, diff = 1.73 ppm.

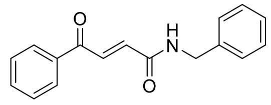

“CAA5”, (E)-N-benzyl-4-oxo-4-phenylbut-2-enamide
General protocol *small scale carbonylacrylic amide synthesis with HATU* was followed for benzylamine (12 µL, 0.11 mmol) and (E)-4-oxo-4-phenylbut-2-enoic acid (18.7 mg, 0.106 mmol) to yield (E)-N-benzyl-4-oxo-4-phenylbut-2-enamide (6.4 mg, 0.0241 mmol, 23%) as a white solid after purification with HPLC (C8 column, 5% to 80% acetonitrile/water, 0.1% TFA). ^{1}H NMR (400 MHz, DMSO) δ 9.10 (t, *J* = 6.0 Hz, 1H), 8.07 – 8.00 (m, 2H), 7.81 (d, *J* = 15.3 Hz, 1H), 7.59 (t, *J* = 7.7 Hz, 2H), 7.40 – 7.23 (m, 5H), 7.06 (d, *J* = 15.3 Hz, 1H), 4.44 (d, *J* = 5.9 Hz, 2H). ^{13}C NMR (101 MHz, DMSO) δ 190.3, 163.9, 139.2, 137.1, 136.8, 134.2, 132.7, 129.5, 129.1, 128.9, 127.9, 127.5, 43.0. Chemical Formula: C17H15NO2. (M+Na)$^{+}$, m/z (calc) = 288.0995, found = 288.0997, diff = 0.55 ppm.

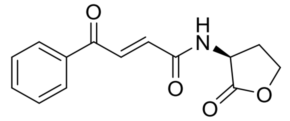

CAA-L, (S,E)-4-oxo-N-(2-oxotetrahydrofuran-3-yl)-4-phenylbut-2-enamide
General protocol *small scale carbonylacrylic amide synthesis with HATU* was followed for (S)-3-aminodihydrofuran-2(3H)-one hydrochloride (20.1 mg, 0.110 mmol) and (E)-4-oxo-4-phenylbut-2-enoic acid (17.6 mg, 0.100 mmol) to yield (S,E)-4-oxo-N-(2-oxotetrahydrofuran-3-yl)-4-phenylbut-2-enamide (4.7 mg, 0.016 mmol, 15%) as a white solid after purification with HPLC (C8 column, 5% to 80% acetonitrile/water, 0.1% TFA). ^{1}H NMR (400 MHz, DMSO) δ 9.14 (d, *J* = 7.9 Hz, 1H), 8.04 (dt, *J* = 7.2, 1.4 Hz, 2H), 7.82 (d, *J* = 15.3 Hz, 1H), 7.75 – 7.69 (m, 1H), 7.60 (dd, *J* = 8.3, 7.2 Hz, 2H), 6.98 (d, *J* = 15.4 Hz, 1H), 4.75 (ddd, *J* = 10.9, 9.1, 7.9 Hz, 1H), 4.42 (td, *J* = 8.8, 1.8 Hz, 1H), 4.28 (ddd, *J* = 10.6, 8.7, 6.4 Hz, 1H), 2.24 (dtd, *J* = 12.2, 10.7, 8.9 Hz, 1H). ^{13}C NMR (101 MHz, DMSO) δ 190.1, 175.3, 164.0, 136.9, 135.6, 134.3, 133.4, 129.5, 129.2, 65.9, 48.9, 28.8. Chemical Formula: C14H13NO4. (M+Na)$^{+}$, m/z (calc) = 282.0737, found = 282.0738, diff = 0.52 ppm.

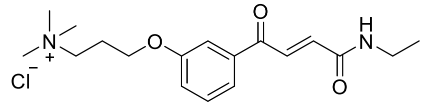

CAA-Q, (E)-3-(3-(4-(ethylamino)-4-oxobut-2-enoyl)phenoxy)-N,N,N-trimethylpropan-1-aminium chloride
General protocol *ether synthesis from alkyl bromides and phenols* was followed for 3-bromopropyl(trimethyl)azanium bromide (13.0 mg, 0.050 mmol) and (E)-N-ethyl-4-(3-hydroxyphenyl)-4-oxobut-2-enamide (10.8 mg, 0.049 mmol) to yield (E)-3-(3-(4-(ethylamino)-4-oxobut-2-enoyl)phenoxy)-N,N,N-trimethylpropan-1-aminium (3.3 mg, 0.010 mmol, 21%) as a yellow oil after purification with HPLC (C8 column, 5% to 80% acetonitrile/water, 0.1% TFA). ^{1}H NMR (400 MHz, DMSO) δ 8.62 (t, *J* = 5.6 Hz, 1H), 7.74 (d, *J* = 15.3 Hz, 1H), 7.66 (dt, *J* = 7.7, 1.1 Hz, 1H), 7.53 (t, *J* = 8.0 Hz, 1H), 7.50 (dd, *J* = 2.6, 1.6 Hz, 1H), 7.30 (ddd, *J* = 8.3, 2.7, 0.9 Hz, 1H), 6.99 (d, *J* = 15.3 Hz, 1H), 4.17 (t, *J* = 5.9 Hz, 2H), 3.56 – 3.45 (m, 2H), 3.23 (qd, *J* = 7.3, 5.5 Hz, 2H), 3.11 (s, 9H), 2.22 (dq, *J* = 11.6, 5.9 Hz, 3H), 1.09 (td, *J* = 7.3, 1.2 Hz, 3H). ^{13}C NMR (101 MHz, DMSO) δ 189.9, 163.5, 159.0, 138.5, 137.2, 132.0, 130.8, 122.0, 120.7, 114.2, 65.5, 63.5, 52.8, 34.3, 23.0, 14.9. Chemical Formula: C18H27N2O3Cl. (M-Cl)$^{+}$, m/z (calc) = 319.2016, found = 319.2035, diff = 5.95 ppm.

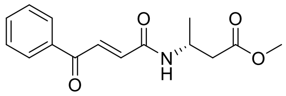

"CAA-A1", methyl (R,E)-3-(4-oxo-4-phenylbut-2-enamido)butanoate
General protocol *small scale carbonylacrylic amide synthesis with HATU* was followed for (R)-methyl 3-aminobutanoate (11.7 mg, 0.100 mmol) and (E)-4-oxo-4-phenylbut-2-enoic acid (17.6 mg, 0.100 mmol) to yield methyl (R,E)-3-(4-oxo-4-phenylbut-2-enamido)butanoate (3.8 mg, 0.014 mmol, 14%) as a colorless oil after purification with HPLC (C8 column, 5% to 80% acetonitrile/water, 0.1% TFA). ^{1}H NMR (400 MHz, DMSO) δ 8.60 (d, *J* = 7.7 Hz, 1H), 8.05 – 7.98 (m, 2H), 7.75 (d, *J* = 15.3 Hz, 1H), 7.72 – 7.68 (m, 1H), 7.66 – 7.55 (m, 2H), 6.95 (d, *J* = 15.3 Hz, 1H), 4.23 (dq, *J* = 7.8, 6.7 Hz, 1H), 3.61 (s, 3H), 2.58 (dd, *J* = 15.3, 6.7 Hz, 1H), 2.50 – 2.44 (m, 1H), 1.17 (d, *J* = 6.7 Hz, 3H). ^{13}C NMR (101 MHz, DMSO) δ 190.3, 171.5, 163.0, 137.1, 137.0, 134.2, 132.4, 129.5, 129.1, 51.9, 42.8, 40.5, 20.4. Chemical Formula: C15H17NO4. $(M+H)^+$, m/z (calc) = 276.1230, found = 276.1239, diff = 3.15 ppm.

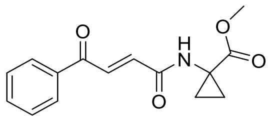

"CAA-A2", methyl (E)-1-(4-oxo-4-phenylbut-2-enamido)cyclopropane-1-carboxylate
General protocol *small scale carbonylacrylic amide synthesis with HATU* was followed for methyl 1-aminocyclopropanecarbonxylate (11.5 mg, 0.100 mmol) and (E)-4-oxo-4-phenylbut-2-enoic acid (17.6 mg, 0.100 mmol) to yield methyl (E)-1-(4-oxo-4-phenylbut-2-enamido)cyclopropane-1-carboxylate (12.8 mg, 0.047 mmol, 47%) as a white solid after purification with HPLC (C8 column, 5% to 80% acetonitrile/water, 0.1% TFA). ^{1}H NMR (400 MHz, DMSO) δ 9.20 (s, 1H), 8.03 (dt, *J* = 7.0, 1.4 Hz, 2H), 7.80 (d, *J* = 15.4 Hz, 1H), 7.75 – 7.68 (m, 1H), 7.59 (dd, *J* = 8.4, 7.1 Hz, 2H), 6.93 (d, *J* = 15.3 Hz, 1H), 3.62 (s, 3H), 1.45 (q, *J* = 4.6 Hz, 2H), 1.12 (q, *J* = 4.6 Hz, 2H). ^{13}C NMR (101 MHz, DMSO) δ 190.1, 172.6, 164.9, 136.9, 136.1, 134.3, 133.0, 129.5, 129.1, 52.7, 33.5, 17.3. Chemical Formula: C15H15NO4. $(M+H)^+$, m/z (calc) = 274.1074, found = 274.1084, diff = 3.84 ppm.

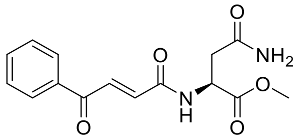

"CAA-A3", methyl (E)-(4-oxo-4-phenylbut-2-enoyl)-L-asparaginate
General protocol *small scale carbonylacrylic amide synthesis with HATU* was followed for L-Asparagine methyl ester hydrochloride (~18 mg, ~0.10 mmol) and (E)-4-oxo-4-phenylbut-2-enoic acid (17.6 mg, 0.100 mmol) to yield methyl (E)-(4-oxo-4-phenylbut-2-enoyl)-L-asparaginate (2.7 mg, 0.009 mmol, 9%) as a white solid after purification with HPLC (C8 column, 5% to 80% acetonitrile/water, 0.1% TFA). ^{1}H NMR (400 MHz, DMSO) δ 9.03 (d, *J* = 7.7 Hz, 1H), 8.02 (dt, *J* = 7.1, 1.3 Hz, 2H), 7.77 (d, *J* = 15.4 Hz, 1H), 7.73 – 7.68 (m, 1H), 7.59 (t, *J* = 7.7 Hz, 2H), 7.44 (s, 1H), 7.08 (d, *J* = 15.4 Hz, 1H), 7.03 – 6.90 (m, 1H), 4.73 (td, *J* = 7.4, 5.5 Hz, 1H), 3.64 (s, 3H), 2.61 (qd, *J* = 15.8, 6.4 Hz, 2H). ^{13}C NMR (101 MHz, DMSO) δ 190.3, 171.9, 171.0, 163.7, 137.0, 136.1, 134.3, 133.2, 129.5, 129.1, 52.5, 49.6, 37.0. Chemical Formula: C15H16N2O5. $(M+Na)^+$, m/z (calc) = 327.0951, found = 327.0967, diff = 4.61 ppm.

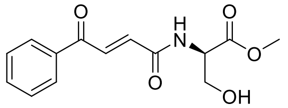

"CAA-A4", methyl (E)-(4-oxo-4-phenylbut-2-enoyl)-D-serinate

General protocol *small scale carbonylacrylic amide synthesis with HATU* was followed for D-Serine methyl ester hydrochloride (17.2 mg, 0.111 mmol) and (E)-4-oxo-4-phenylbut-2-enoic acid (18.3 mg, 0.104 mmol) to yield methyl (E)-(4-oxo-4-phenylbut-2-enoyl)-D-serinate (8.4 mg, 0.030 mmol, 30%) as a white solid after purification with HPLC (C8 column, 5% to 80% acetonitrile/water, 0.1% TFA). ^{1}H NMR (400 MHz, DMSO) δ 9.02 (d, *J* = 7.7 Hz, 1H), 8.06 – 7.98 (m, 2H), 7.78 (d, *J* = 15.4 Hz, 1H), 7.74 – 7.68 (m, 1H), 7.59 (dd, *J* = 8.4, 7.1 Hz, 2H), 7.17 (d, *J* = 15.4 Hz, 1H), 4.52 (dt, *J* = 7.7, 4.8 Hz, 1H), 3.78 (dd, *J* = 11.0, 5.4 Hz, 1H), 3.71 (t, *J* = 4.2 Hz, 1H), 3.67 (s, 3H). ^{13}C NMR (101 MHz, DMSO) δ 190.4, 171.1, 164.0, 137.0, 136.3, 134.2, 133.2, 129.5, 129.1, 61.6, 55.5, 52.5. Chemical Formula: C14H15NO5. (M+Na)$^+$, m/z (calc) = 300.0843, found = 300.0843, diff = 0.20 ppm.

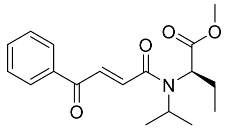

"CAA-A5", methyl (R,E)-2-(N-isopropyl-4-oxo-4-phenylbut-2-enamido)butanoate
General protocol *small scale carbonylacrylic amide synthesis with HATU* was followed for methyl (R)-2-(isopropylamino)butanoate (15.9 mg, 0.100 mmol) and (E)-4-oxo-4-phenylbut-2-enoic acid (17.6 mg, 1.00 mmol) reacted to yield methyl (R,E)-2-(N-isopropyl-4-oxo-4-phenylbut-2-enamido)butanoate as a yellow oil (1.9 mg, 0.0060 mmol, 6%). Mixture of rotamers. ^{1}H NMR (500 MHz, DMSO) δ 8.05 (dt, *J* = 10.0, 4.4 Hz, 2H), 7.76 – 7.62 (m, 2H), 7.59 (dt, *J* = 12.0, 6.1 Hz, 2H), 7.48 – 7.34 (m, 1H), 4.27 (h, *J* = 6.7 Hz, 1H), 4.04 (q, *J* = 6.5 Hz, 1H), 3.91 (q, *J* = 6.3 Hz, 1H), 3.71 (t, *J* = 11.5 Hz, 1H), 3.63 – 3.58 (m, 3H), 2.30 – 2.20 (m, 1H), 1.78 – 1.67 (m, 1H), 1.39 – 1.33 (m, 2H), 1.27 – 1.17 (m, 6H), 1.01 – 0.90 (m, 2H). Chemical Formula: C18H23NO4. (M+Na)$^+$, m/z (calc) = 340.1519, found = 340.1511, diff = -2.35 ppm.

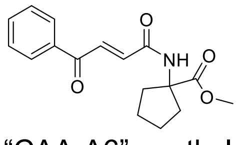

"CAA-A6", methyl (E)-1-(4-oxo-4-phenylbut-2-enamido)cyclopentane-1-carboxylate
General protocol *small scale carbonylacrylic amide synthesis with HATU* was followed for methyl 1-aminocyclopentane-1-carboxylate (13.2 µL, 0.100 mmol) and (E)-4-oxo-4-phenylbut-2-enoic acid (17.8 mg, 1.01 mmol) reacted to yield methyl (E)-1-(4-oxo-4-phenylbut-2-enamido)cyclopentane-1-carboxylate (15.4 mg, 0.051 mmol, 51%) as a white crystalline solid. ^{1}H NMR (400 MHz, DMSO) δ 8.97 (s, 1H), 8.06 – 7.98 (m, 2H), 7.78 – 7.67 (m, 2H), 7.59 (t, *J* = 7.7 Hz, 2H), 7.03 (d, *J* = 15.4 Hz, 1H), 3.60 (s, 3H), 2.13 (dt, *J* = 13.1, 7.9 Hz, 2H), 1.98 – 1.87 (m, 2H), 1.70 (td, *J* = 8.6, 4.9 Hz, 4H). ^{13}C NMR (101 MHz, DMSO) δ 190.3, 174.1, 163.5, 137.0, 136.4, 134.2, 132.8, 129.5, 129.1, 65.8, 52.5, 36.8, 24.4. Chemical Formula: C17H19NO4. (M+H)$^+$, m/z (calc) = 302.1387, found = 302.1397, diff = 3.43 ppm.

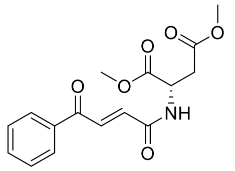

"CAA-AA", dimethyl (E)-(4-oxo-4-phenylbut-2-enoyl)-L-aspartate
At 20°C, to a stirred suspension of (E)-4-oxo-4-phenylbut-2-enoic acid (17.6 mg, 0.100 mmol) and HATU (38 mg, 0.10 mmol) in DCM (2 mL) was added diisopropylethylamine (0.080 mL). After stirring for 5 min, dimethyl L-aspartate hydrochloride (22.3 mg, 0.113 mmol) was added to the mixture. After stirring for 16 h at 20°C, the mixture was diluted into ethyl ether (5 mL) and

washed with saturated aqueous ammonia chloride (5 mL). The organic fraction was dried and concentrated by evaporation. The organic residue was purified by HPLC (C8, 5% to 75% acetonitrile/water, 0.1% TFA) to give dimethyl (E)-(4-oxo-4-phenylbut-2-enoyl)-L-aspartate (14.8 mg, 0.046 mmol, 46%) as a white solid. ^{1}H NMR (400 MHz, DMSO) δ 9.14 (d, *J* = 7.8 Hz, 1H), 8.06 – 7.99 (m, 2H), 7.80 (d, *J* = 15.4 Hz, 1H), 7.75 – 7.68 (m, 1H), 7.59 (dd, *J* = 8.3, 7.1 Hz, 2H), 7.04 (d, *J* = 15.4 Hz, 1H), 4.79 (td, *J* = 7.4, 5.7 Hz, 1H), 3.67 (s, 3H), 3.63 (s, 3H), 2.86 (qd, *J* = 16.6, 6.5 Hz, 2H). ^{13}C NMR (101 MHz, DMSO) δ 190.2, 171.2, 170.8, 163.8, 136.9, 135.8, 134.3, 133.4, 129.5, 129.2, 52.8, 52.3, 49.3, 35.9. Chemical Formula: C16H17NO6. $(M+Na)^+$, m/z (calc) = 342.0948, found = 342.0947, diff = -0.36 ppm.

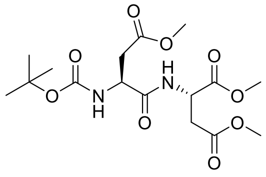

dimethyl ((S)-2-((tert-butoxycarbonyl)amino)-4-methoxy-4-oxobutanoyl)-L-aspartate
At 20°C, HATU (800 mg, 2.10 mmol) and DIPEA (1.56 mL, ~ 9 mmol) were added to a solution of (3S)-3-[(tert-butoxy)carbonyl]amino-4-methoxy-4-oxobutanoic acid (494 mg, 2.00 mmol) and dimethyl L-aspartate hydrochloride (394 mg, 2.00 mmol) in DCM (6 mL). After 16 h, the reaction mixture was diluted into ethyl acetate (100 mL), washed sequentially with 0.1 N aqueous HCl (thrice, 50 mL each), 5% aqueous $NaHCO_3$ (twice, 50 mL each), and saturated aqueous NaCl (50 mL). The organic fraction was dried over $MgSO_4$ and concentrated under reduced pressure. The organic residue was purified by flash column chromatography (silica, 25 to 35% ethyl acetate/hexanes) to give dimethyl ((S)-2-((tert-butoxycarbonyl)amino)-4-methoxy-4-oxobutanoyl)-L-aspartate (480 mg, 1.23 mmol, 62%) as a viscous colorless oil. R_f = 0.20 in 35% ethyl acetate/hexanes. ^{1}H NMR (500 MHz, Acetone) δ 7.64 (d, *J* = 8.0 Hz, 1H), 6.39 (d, *J* = 8.3 Hz, 1H), 4.81 (dt, *J* = 8.3, 5.5 Hz, 1H), 4.52 (q, *J* = 7.1 Hz, 1H), 3.70 (s, 3H), 3.66 (s, 3H), 3.65 (s, 3H), 2.94 – 2.70 (m, 4H), 1.44 (s, 9H). Spectrum matches precedence.[13]

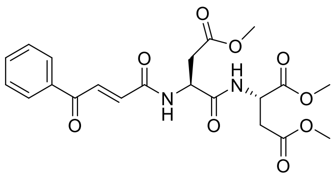

"CAA-Asp$_2$", dimethyl ((S)-4-methoxy-4-oxo-2-((E)-4-oxo-4-phenylbut-2-enamido)butanoyl)-L-aspartate
Boc-deprotection of dimethyl ((S)-2-((tert-butoxycarbonyl)amino)-4-methoxy-4-oxobutanoyl)-L-aspartate was performed with 50% TFA/DCM at 20°C for 2 h. The reaction mixture was concentrated under reduced pressure and passed through a plug of silica (eluted with methanol in DCM) to give crude dimethyl ((S)-2-amino-4-methoxy-4-oxobutanoyl)-L-aspartate as a white solid which was used directly for the next amide coupling step without further purification. Thus, at 27°C, HATU and DIPEA were added to a solution of crude dimethyl ((S)-2-amino-4-methoxy-4-oxobutanoyl)-L-aspartate (45 mg, 0.155 mmol) and (E)-4-oxo-4-phenylbut-2-enoic acid (37 mg, 0.210 mmol) in DCM (4 mL). After 16 h, the reaction mixture was diluted into ethyl acetate (20 mL), washed sequentially with 0.1 N aqueous HCl (twice, 10 mL each), 5% aqueous $NaHCO_3$ (10 mL), and saturated aqueous NaCl. The organic fraction was concentrated under reduced pressure and purified by HPLC (C8, 5% acetonitrile/water to 95% acetonitrile/water, 0.1% TFA) to give dimethyl ((S)-4-methoxy-4-oxo-2-((E)-4-oxo-4-phenylbut-2-

enamido)butanoyl)-L-aspartate (24.8 mg, 0.0553 mmol, 36%) as a white solid. ^{1}H NMR (500 MHz, Acetone) δ 8.15 (d, *J* = 8.2 Hz, 1H), 8.11 – 8.05 (m, 2H), 7.89 (d, *J* = 15.3 Hz, 1H), 7.81 (d, *J* = 8.2 Hz, 1H), 7.75 – 7.68 (m, 1H), 7.61 (dddd, *J* = 7.9, 6.8, 1.5, 1.0 Hz, 2H), 7.11 (d, *J* = 15.3 Hz, 1H), 4.98 (dt, *J* = 8.3, 6.5 Hz, 1H), 4.83 (dt, *J* = 8.3, 5.7 Hz, 1H), 3.70 (s, 3H), 3.67 (s, 3H), 3.64 (s, 3H), 2.98 – 2.76 (m, 4H). ^{13}C NMR (126 MHz, Acetone) δ 189.4, 170.7, 170.6, 170.5, 169.8, 163.9, 137.1, 135.2, 133.6, 133.1, 128.9, 128.6, 51.8, 51.2, 51.1, 49.9, 48.9, 35.6, 35.6. Chemical Formula: C21H24N2O9. $(M+Na)^{+}$, m/z (calc) = 471.1374, found = 471.1376, diff = 0.51 ppm.

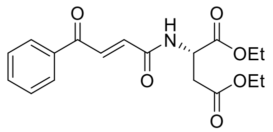

"CAA-AAR", diethyl (E)-(4-oxo-4-phenylbut-2-enoyl)-L-aspartate

To a stirred suspension of (E)-4-oxo-4-phenylbut-2-enoic acid (44.6 mg, 0.253 mmol) and HATU (91 mg, 0.239 mmol) in anhydrous DCM (2 mL) was added DIPEA (0.32 mL). After stirring for 5 min, diethyl L-aspartate hydrochloride (60.0 mg, 0.266 mmol) was added to the mixture. After stirring for 16 h at 27 °C, the mixture was diluted into ethyl ether (10 mL) and washed with saturated aqueous ammonia chloride (5 mL). The organic fraction was dried and concentrated by evaporation. The organic residue was purified by HPLC (C8, 5% acetonitrile/water to 80% acetonitrile/water, 0.1% TFA) to give diethyl (E)-(4-oxo-4-phenylbut-2-enoyl)-L-aspartate (52.9 mg, 0.152 mmol, 60%) as a white solid. ^{1}H NMR (500 MHz, Acetone) δ 8.10 – 8.04 (m, 2H), 7.89 (d, *J* = 15.3 Hz, 1H), 7.74 – 7.67 (m, 1H), 7.60 (t, *J* = 7.8 Hz, 2H), 7.17 (d, *J* = 15.2 Hz, 1H), 4.96 (t, *J* = 5.9 Hz, 1H), 4.26 – 4.05 (m, 4H), 2.94 (d, *J* = 5.9 Hz, 2H), 1.24 (dt, *J* = 11.4, 7.1 Hz, 6H). ^{13}C NMR (126 MHz, Acetone) δ 189.4, 170.1, 169.9, 163.4, 137.1, 135.2, 133.5, 133.2, 128.9, 128.6, 61.1, 60.4, 49.2, 36.1, 13.6, 13.5. Chemical Formula: C18H21NO6. $(M+Na)^{+}$, m/z (calc) = 370.1261, found = 370.1261, diff = 0.03 ppm.

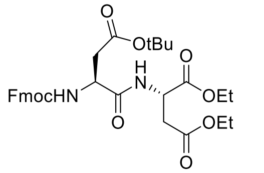

diethyl ((S)-2-((((9H-fluoren-9-yl)methoxy)carbonyl)amino)-4-(tert-butoxy)-4-oxobutanoyl)-L-aspartate

At 15°C, HATU (758 mg, 1.99 mmol) and DIPEA (1.50 mL, ~ 8 mmol) were added to a solution of (S)-2-((((9H-fluoren-9-yl)methoxy)carbonyl)amino)-4-(tert-butoxy)-4-oxobutanoic acid (800 mg, 1.94 mmol) and diethyl L-aspartate hydrochloride (452 mg, 2.00 mmol) in DCM (12 mL). After 16 h, the reaction mixture was diluted into ethyl acetate (100 mL), washed sequentially with 0.1 N aqueous HCl (twice, 50 mL each), 5% aqueous $NaHCO_3$ (50 mL), and saturated aqueous NaCl (50 mL). The organic fraction was dried over $MgSO_4$ and concentrated under reduced pressure. The organic residue was purified by flash column chromatography (silica, 20 to 30% ethyl acetate/hexanes) to give diethyl ((S)-2-((((9H-fluoren-9-yl)methoxy)carbonyl)amino)-4-(tert-butoxy)-4-oxobutanoyl)-L-aspartate (969 mg, 1.66 mmol, 86%) as a white solid. ^{1}H NMR (400 MHz, Acetone) δ 7.88 (d, *J* = 7.5 Hz, 2H), 7.71 (t, *J* = 7.1 Hz, 3H), 7.43 (t, *J* = 7.4 Hz, 2H), 7.35 (t, *J* = 7.4 Hz, 2H), 6.90 (d, *J* = 8.6 Hz, 1H), 4.84 – 4.76 (m, 1H), 4.61 (q, *J* = 7.5 Hz, 1H), 4.41 – 4.23 (m, 3H), 4.15 (qt, *J* = 7.7, 3.9 Hz, 2H), 4.08 (qd, *J* = 7.1, 2.1 Hz, 2H), 2.90 – 2.79 (m, 3H), 2.66 (dd, *J* = 16.3, 7.6 Hz, 1H), 1.45 (s, 9H), 1.21 (dt, *J* = 12.1, 7.1 Hz, 6H). ^{13}C NMR (101 MHz, Acetone) δ 170.4, 170.2, 170.1, 169.6, 156.0, 144.1, 141.2, 127.7, 127.1, 125.3, 119.9, 80.4, 66.6, 61.0, 60.3, 51.6, 48.9, 47.1, 37.4, 35.9, 27.3, 13.5,

13.5. Chemical Formula: C31H38N2O9. $(M+Na)^+$, m/z (calc) = 605.2470, found = 605.2500, diff = 5.04 ppm.

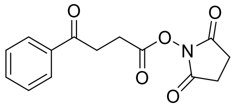

2,5-dioxopyrrolidin-1-yl 4-oxo-4-phenylbutanoate

At 17°C, N,N,N′,N′-Tetramethyl-O-(N-succinimidyl)uronium tetrafluoroborate (980 mg, 3.26 mmol) was added to a solution of 4-oxo-4-phenylbutanoic acid (385 mg, 2.16 mmol) and triethylamine (0.70 mL) in anhydrous DMF (5 mL). The mixture was stirred for 1 h before it was diluted into ethyl ether/ethyl acetate (100/15 mL) and washed sequentially with water (4 times, 25 mL each) and saturated aqueous NaCl (25 mL). The organic fraction was dried over $MgSO_4$ and concentrated under reduced pressure. The organic residue was purified by flash column chromatography (silica, 25 to 35% ethyl acetate/hexanes) to give 2,5-dioxopyrrolidin-1-yl 4-oxo-4-phenylbutanoate (124.8 mg, 0.453 mmol, 21%) as a yellow solid. ^1H NMR (500 MHz, Acetone) δ 8.09 – 8.04 (m, 2H), 7.70 – 7.62 (m, 1H), 7.56 (t, J = 7.8 Hz, 2H), 3.51 (t, J = 6.4 Hz, 2H), 3.09 (t, J = 6.5 Hz, 2H), 2.88 (s, 4H). ^{13}C NMR (126 MHz, Acetone) δ 196.7, 169.7, 168.7, 136.5, 133.3, 128.7, 128.0, 48.9, 32.7, 25.4, 24.9. Chemical Formula: C14H13NO5. $(M+Na)^+$, m/z (calc) = 298.0686, found = 298.0688, diff = 0.84 ppm.

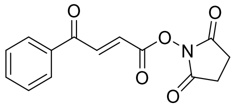

2,5-dioxopyrrolidin-1-yl (E)-4-oxo-4-phenylbut-2-enoate

At 17°C, N,N,N′,N′-Tetramethyl-O-(N-succinimidyl)uronium tetrafluoroborate (950 mg, 3.16 mmol) was added to a solution of (E)-4-oxo-4-phenylbut-2-enoic acid (369 mg, 2.10 mmol) and triethylamine (0.70 mL) in anhydrous DMF (5 mL). The mixture was stirred for 1 h before it was diluted into ethyl ether/ethyl acetate (100/15 mL) and washed sequentially with water (4 times, 25 mL each) and saturated aqueous NaCl (25 mL). The organic fraction was dried over $MgSO_4$ and concentrated under reduced pressure. The organic residue was purified by flash column chromatography (silica, 30 to 35% ethyl acetate/hexanes) to give 2,5-dioxopyrrolidin-1-yl (E)-4-oxo-4-phenylbut-2-enoate (158.5 mg, 0.58 mmol, 28%) as a yellow solid. ^1H NMR (500 MHz, Acetone) δ 8.27 (d, J = 15.7 Hz, 1H), 8.14 (dt, J = 7.1, 1.3 Hz, 2H), 7.78 – 7.71 (m, 1H), 7.63 (t, J = 7.8 Hz, 2H), 7.04 (d, J = 15.7 Hz, 1H), 2.97 (s, 4H). ^{13}C NMR (126 MHz, Acetone) δ 188.2, 169.4, 161.2, 141.2, 136.2, 134.2, 129.1, 129.0, 125.5, 25.5. Chemical Formula: C14H11NO5. $(M+Na)^+$, m/z (calc) = 296.0530, found = 296.0501, diff = -9.69 ppm.

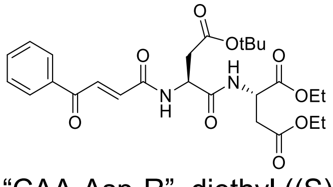

“CAA-Asp_2R”, diethyl ((S)-4-(tert-butoxy)-4-oxo-2-((E)-4-oxo-4-phenylbut-2-enamido)butanoyl)-L-aspartate

Fmoc-deprotection of diethyl ((S)-2-((((9H-fluoren-9-yl)methoxy)carbonyl)amino)-4-(tert-butoxy)-4-oxobutanoyl)-L-aspartate was performed with 20% piperidine/DMF at 20°C for 16 h. The volatile was evaporated under reduced pressure and the residue passed through a plug of silica (eluted with DCM) to give crude diethyl ((S)-2-amino-4-(tert-butoxy)-4-oxobutanoyl)-L-aspartate as a white solid which was used directly for the next amide coupling step without further purification. Thus, 2,5-dioxopyrrolidin-1-yl (E)-4-oxo-4-phenylbut-2-enoate (20 mg, 0.073 mmol)

was added to a solution of diethyl ((S)-2-amino-4-(tert-butoxy)-4-oxobutanoyl)-L-aspartate (26.4 mg, 0.073 mmol) and triethylamine (20 µL, 0.15 mmol) in DCM (3 mL). The mixture was stirred at 20°C for 3 h. The volatile was evaporated under reduced pressure and the organic residue was purified by HPLC (C8, 5% to 75% acetonitrile/water, 0.1% TFA) to give diethyl ((S)-4-(tert-butoxy)-4-oxo-2-((E)-4-oxo-4-phenylbut-2-enamido)butanoyl)-L-aspartate (12.9 mg, 0.025 mmol, 34%) as a yellow solid. ^{1}H NMR (400 MHz, DMSO) δ 8.94 (d, *J* = 8.2 Hz, 1H), 8.52 (d, *J* = 7.9 Hz, 1H), 8.05 – 7.98 (m, 2H), 7.78 (d, *J* = 15.4 Hz, 1H), 7.74 – 7.68 (m, 1H), 7.59 (t, *J* = 7.7 Hz, 2H), 7.04 (d, *J* = 15.4 Hz, 1H), 4.78 (td, *J* = 8.7, 5.1 Hz, 1H), 4.67 – 4.57 (m, 1H), 4.14 – 4.01 (m, 4H), 2.85 – 2.64 (m, 3H), 2.49 – 2.44 (m, 1H), 1.38 (s, 9H), 1.17 (td, *J* = 7.1, 1.8 Hz, 6H). ^{13}C NMR (101 MHz, DMSO) δ 190.3, 170.8, 170.5, 170.3, 169.4, 163.7, 137.0, 136.6, 134.3, 132.8, 129.5, 129.1, 80.8, 61.4, 60.8, 50.0, 49.2, 38.0, 36.1, 28.1, 14.4, 14.4. Chemical Formula: C26H34N2O9. $(M+Na)^+$, m/z (calc) = 541.2157, found = 541.2158, diff = 0.20 ppm.

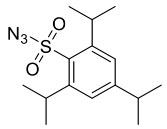

2,4,6-triisopropylbenzenesulfonyl azide
2,4,6-triisopropylbenzenesulfonyl chloride (1.55 g, 5.12 mmol) was dissolved in a solution of acetone/water (5 mL/1 mL) and cooled to 0°C. To the stirred solution was then added, in batches, sodium azide (0.69 g, 10.6 mmol). After addition of sodium azide, the reaction was warmed to 20°C and reaction progress was monitored by TLC (hexanes). After 1.5 h, substrate was fully converted. The reaction mixture was diluted into ethyl acetate (100 mL) and washed sequentially with water (30 mL, twice), saturated sodium bicarbonate (30 mL, twice), water (30 mL, twice). The organic fraction was then dried and concentrated under reduced pressure to give 2,4,6-triisopropylbenzenesulfonyl azide (1.16 g, 3.75 mmol, 73%) as a colorless oil that slowly solidifies into a white solid.

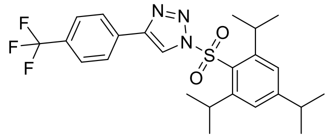

"AHL-003", 4-(4-(trifluoromethyl)phenyl)-1-((2,4,6-triisopropylphenyl)sulfonyl)-1H-1,2,3-triazole
Briefly, to a solution of 2,4,6-triisopropylbenzenesulfonyl azide (562 mg, 1.82 mmol) in toluene (15 mL) at room temperature was added 1-ethynyl-4-(trifluoromethyl)benzene (309 mg, 1.82 mmol) followed by addition of CuTC (36 mg, 0.19 mmol). Upon completion, saturated ammonium chloride solution was added to the reaction mixture and stirred for 15 min before the addition of brine. The mixture was extracted twice with ethyl acetate. The organic layer was washed with brine and the aqueous layer back-extracted with ethyl acetate. The combined organic layer was washed with brine and dried over $MgSO_4$. The crude product was recryallized with hot ethyl acetate and hexanes to yield 4-(4-(trifluoromethyl)phenyl)-1-((2,4,6-triisopropylphenyl)sulfonyl)-1H-1,2,3-triazole (113 mg, 0.236 mmol, 13%) as a white solid. ^{1}H NMR (400 MHz, DMSO) δ 9.74 (s, 1H), 8.26 – 8.19 (m, 2H), 7.91 – 7.84 (m, 2H), 7.45 (s, 2H), 4.11 (h, *J* = 6.6 Hz, 2H), 3.00 (h, *J* = 6.7 Hz, 1H), 1.23 (d, *J* = 6.9 Hz, 6H), 1.17 (d, *J* = 6.8 Hz, 12H), agrees with precedence.[14] Low yield was due to inefficiency in recrystallization.

NMR Spectra

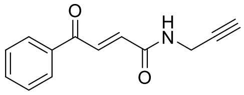

“CAA-alkyne”, (E)-4-oxo-4-phenyl-N-(prop-2-yn-1-yl)but-2-enamide

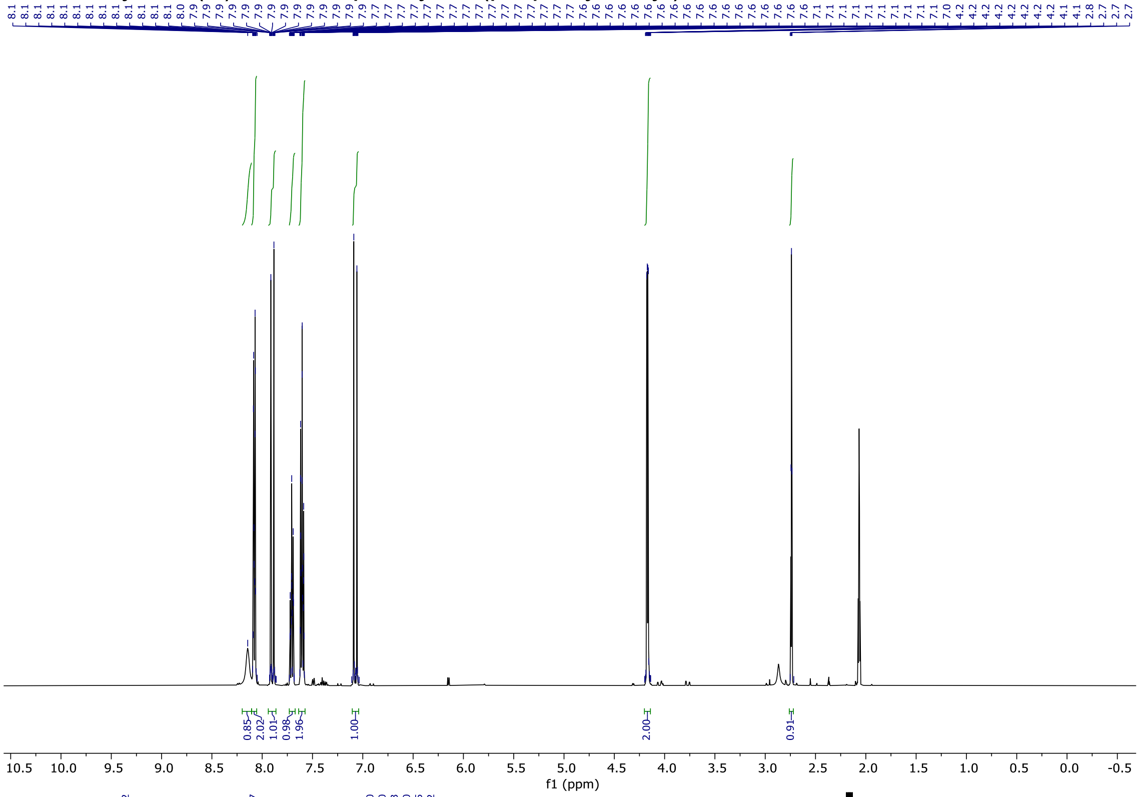

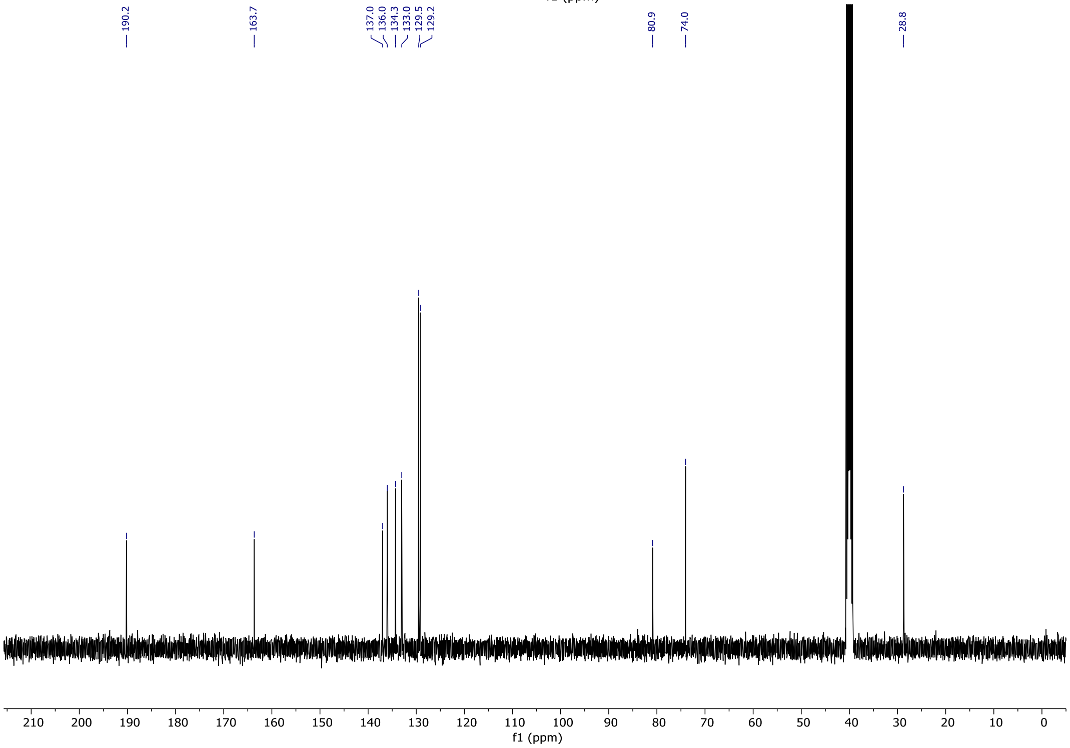

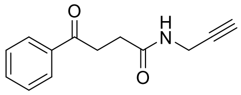

“2H_CAA”, 4-oxo-4-phenyl-N-(prop-2-yn-1-yl)butanamide

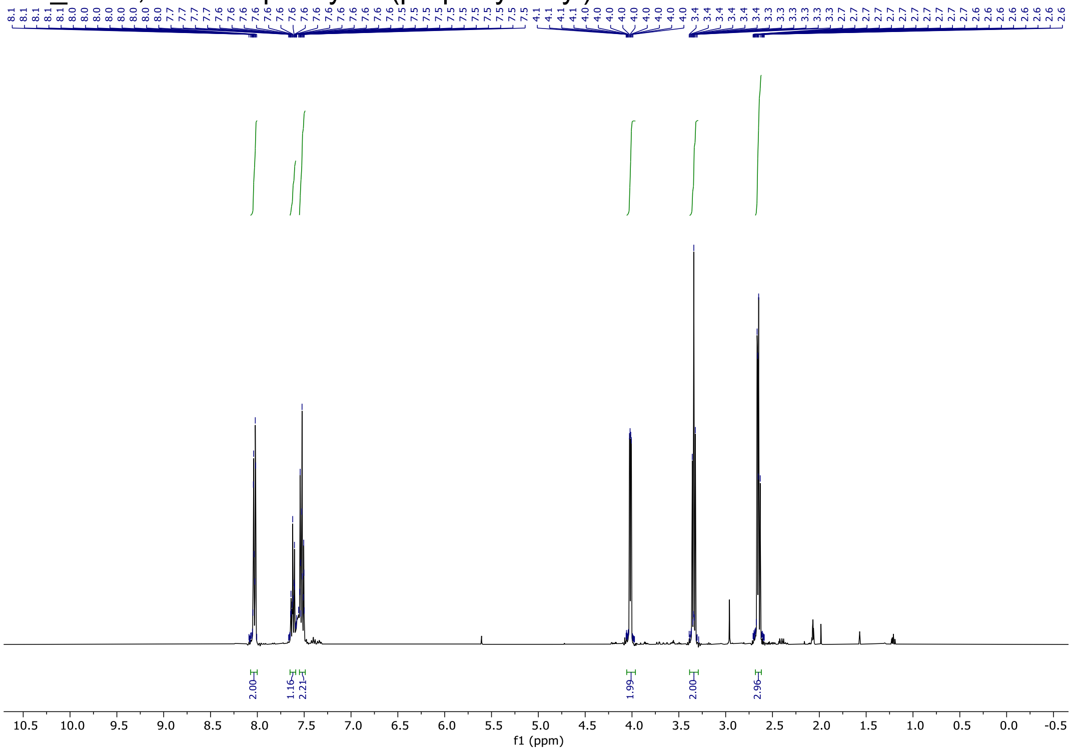

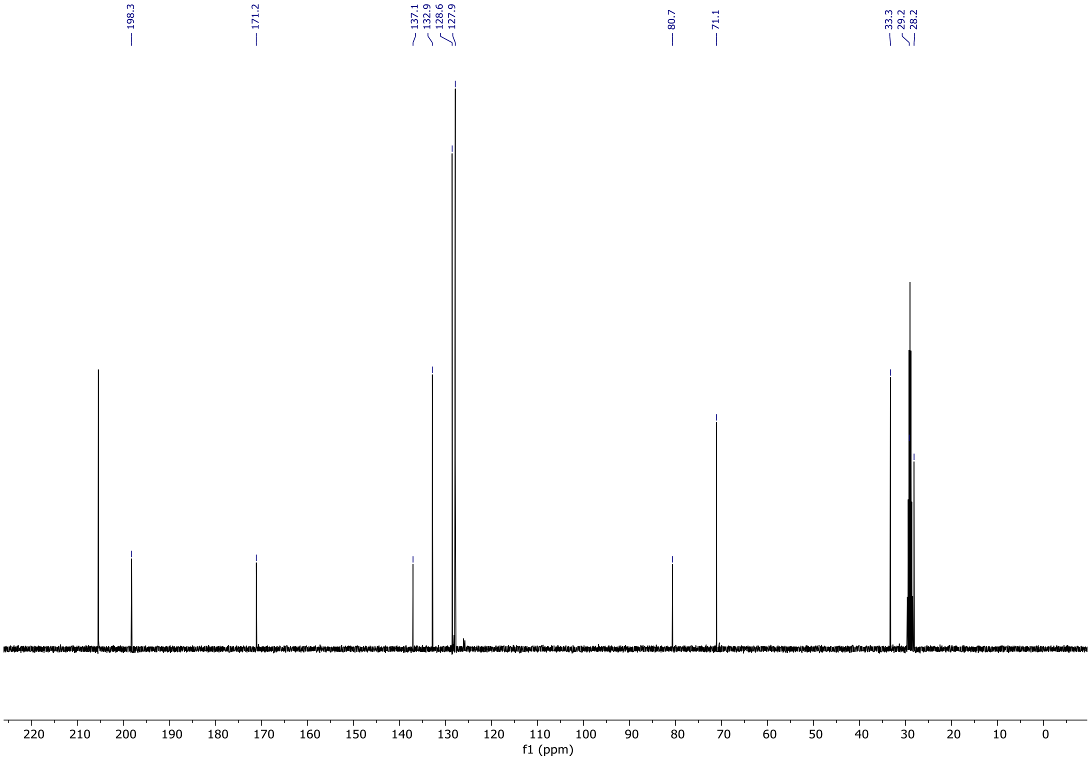

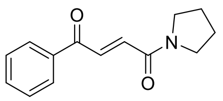

"CAA2", (E)-1-phenyl-4-(pyrrolidin-1-yl)but-2-ene-1,4-dione

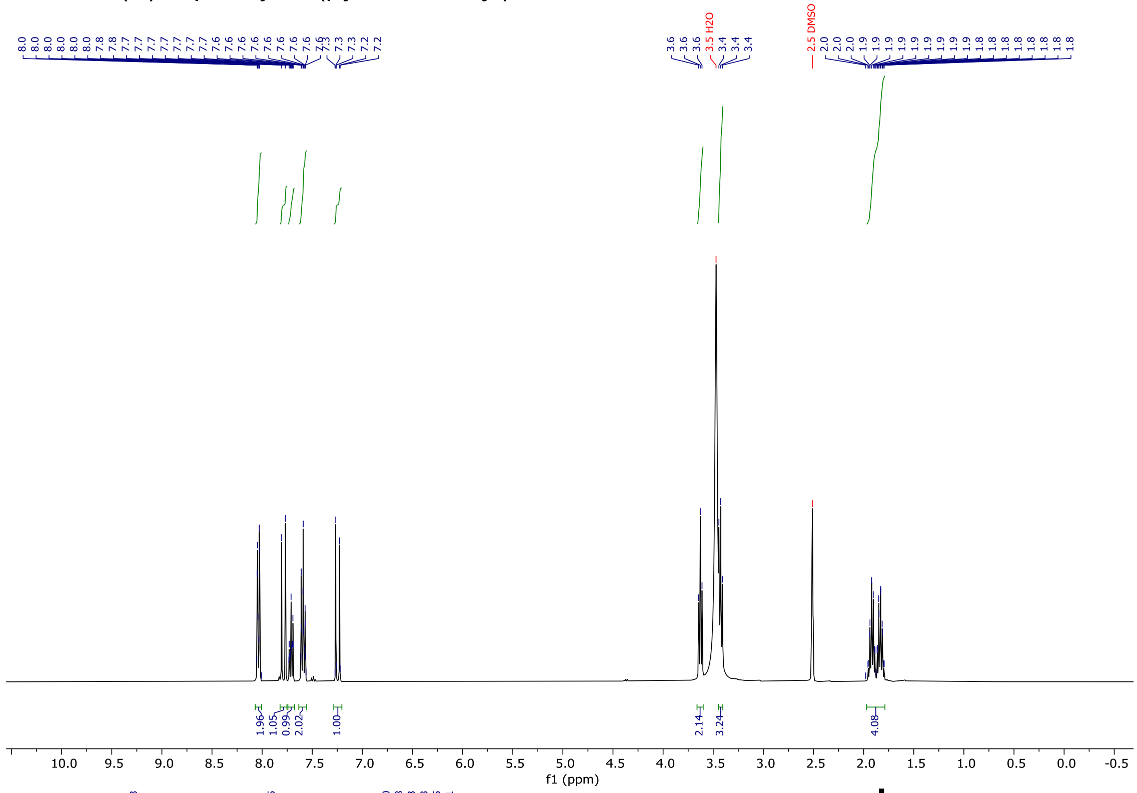

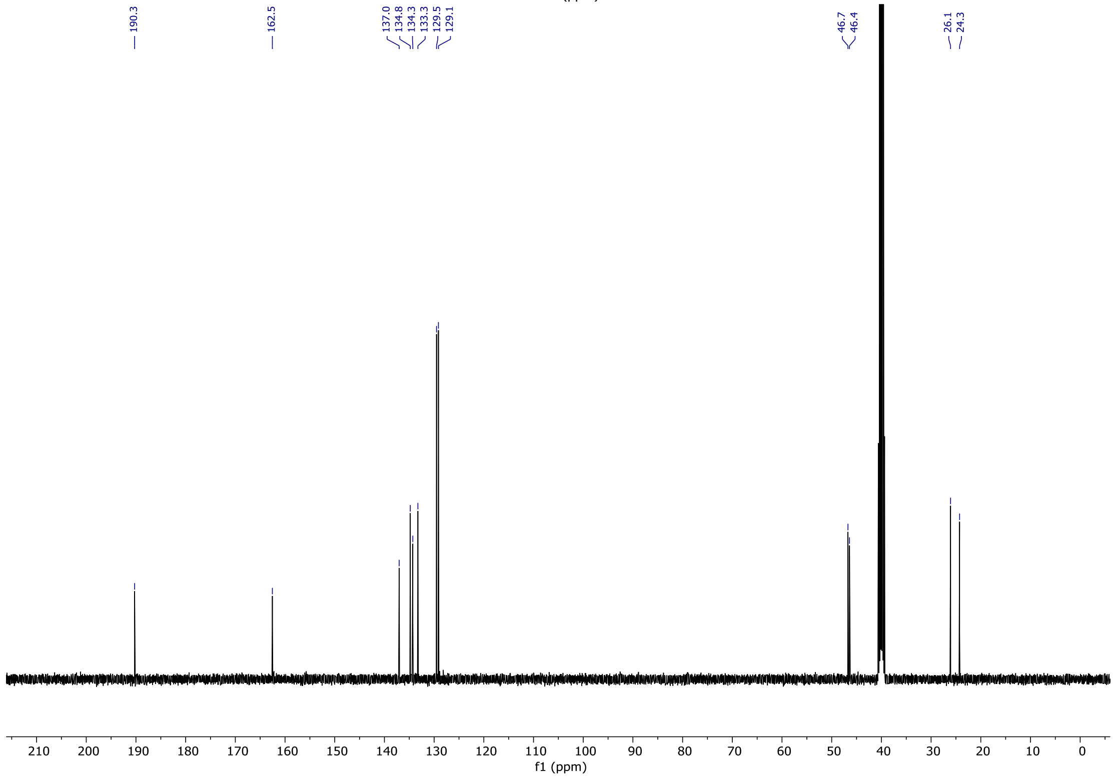

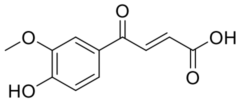

(E)-4-(4-hydroxy-3-methoxyphenyl)-4-oxobut-2-enoic acid

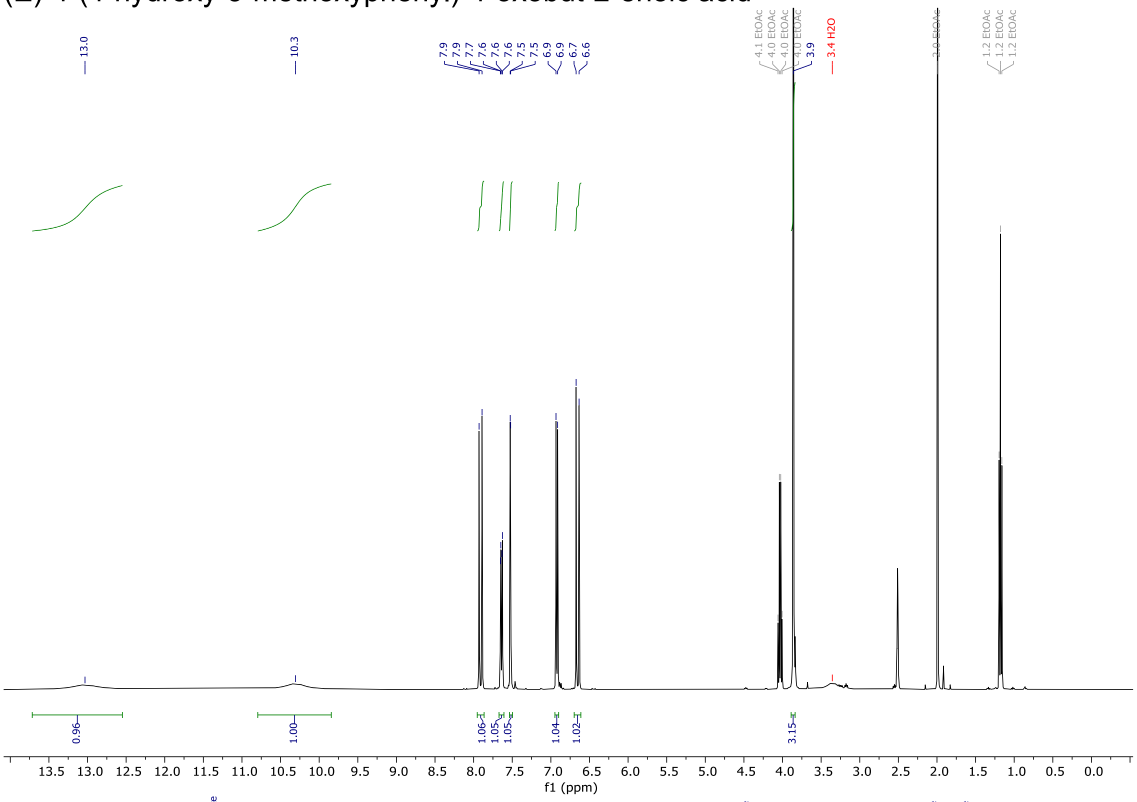

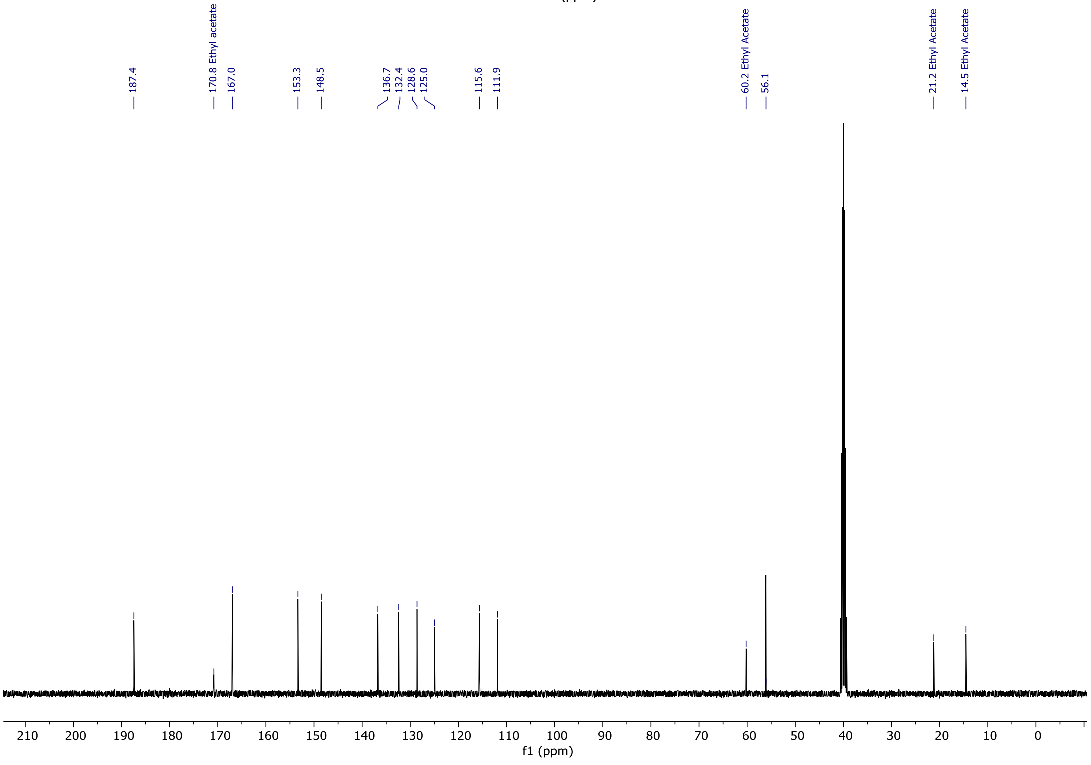

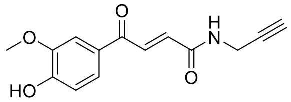

“CAA3”, (E)-4-(4-hydroxy-3-methoxyphenyl)-4-oxo-N-(prop-2-yn-1-yl)but-2-enamide

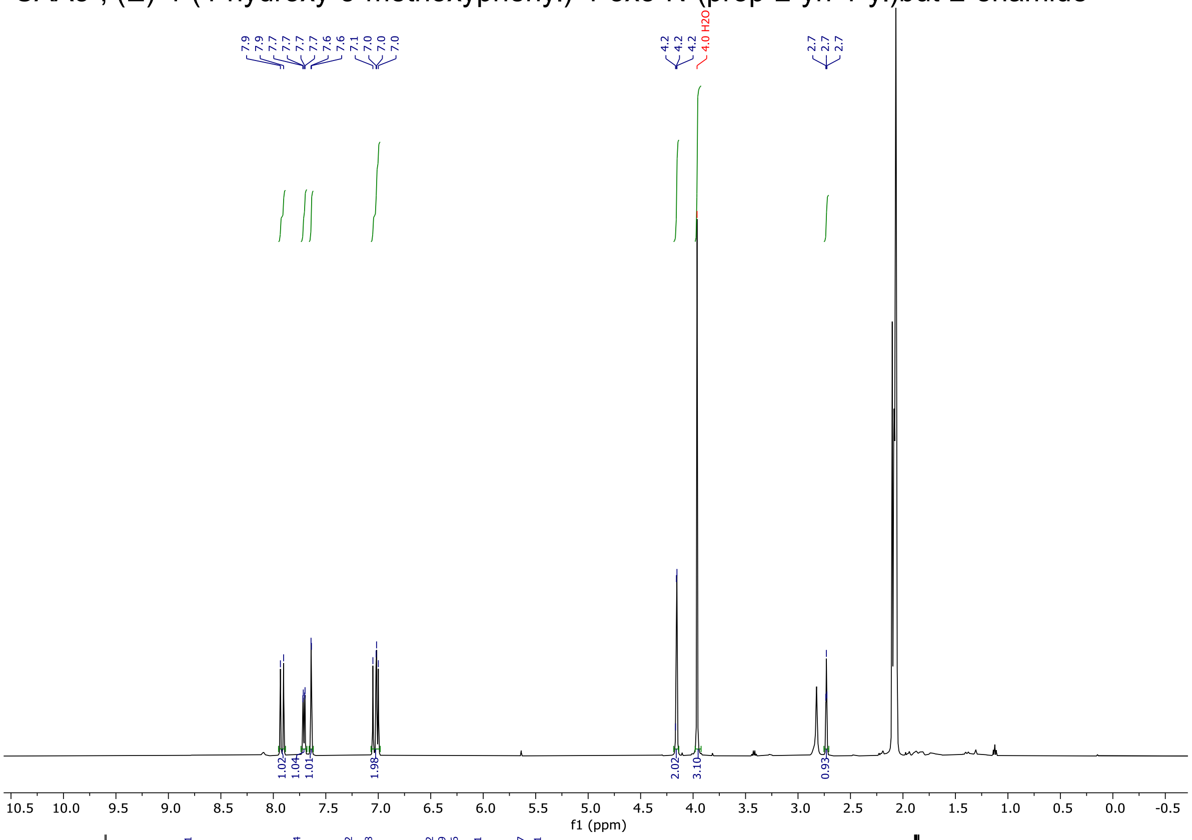

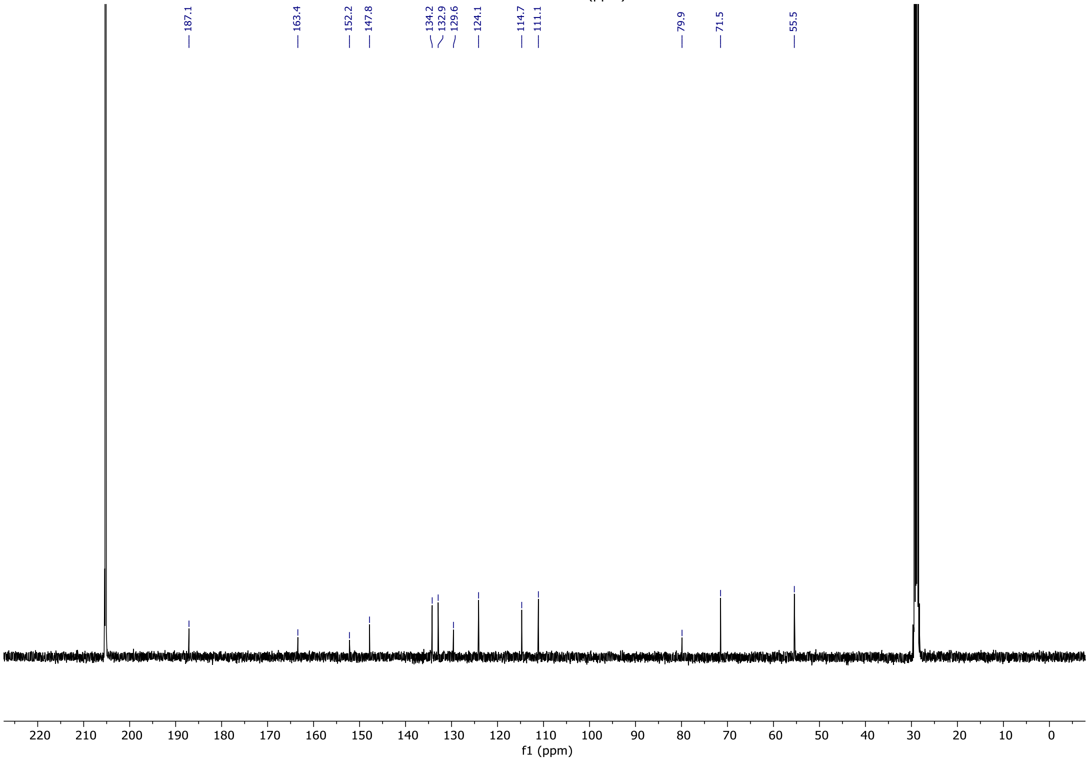

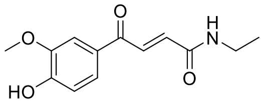

(E)-N-ethyl-4-(4-hydroxy-3-methoxyphenyl)-4-oxobut-2-enamide

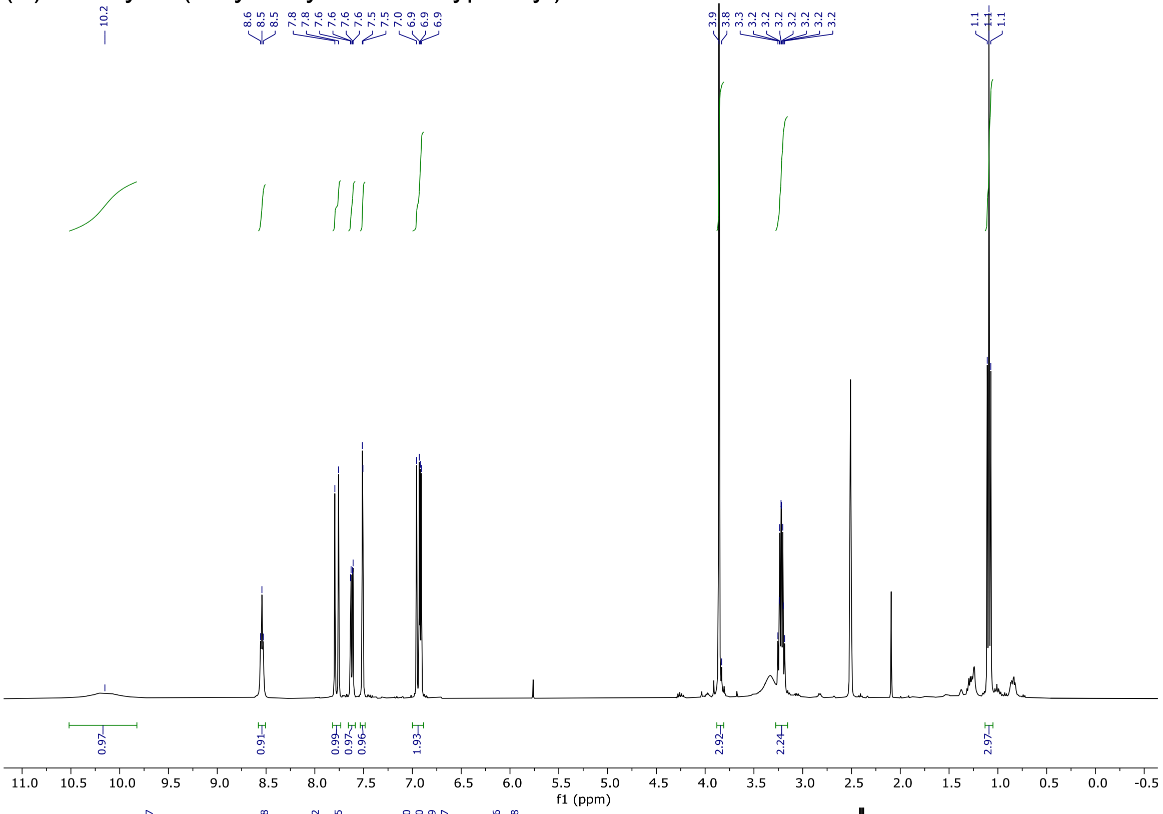

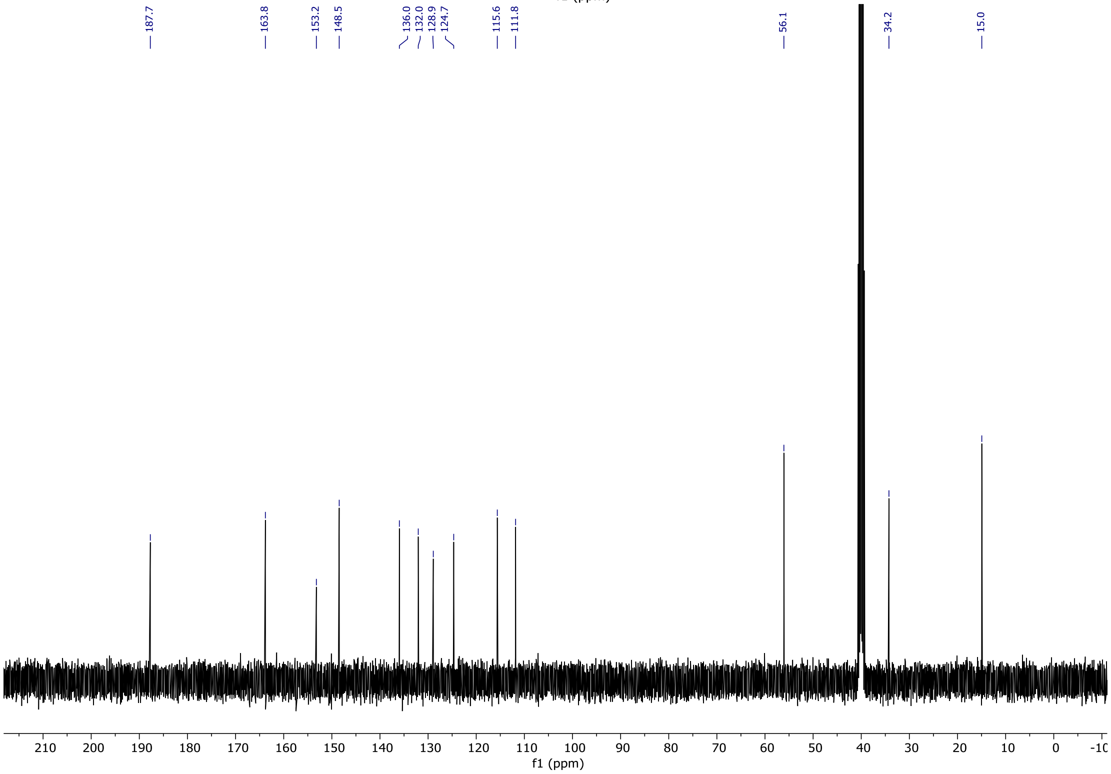

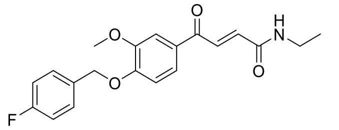

"CAA4", (E)-N-ethyl-4-(4-((4-fluorobenzyl)oxy)-3-methoxyphenyl)-4-oxobut-2-enamide

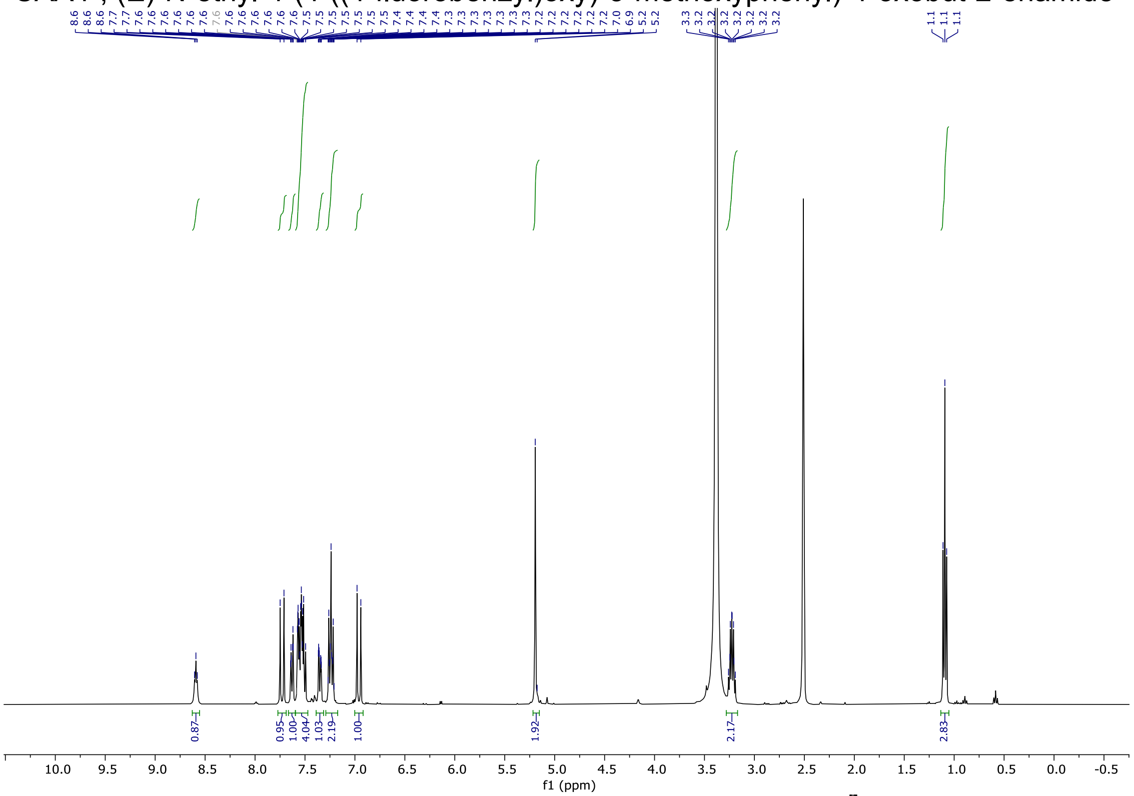

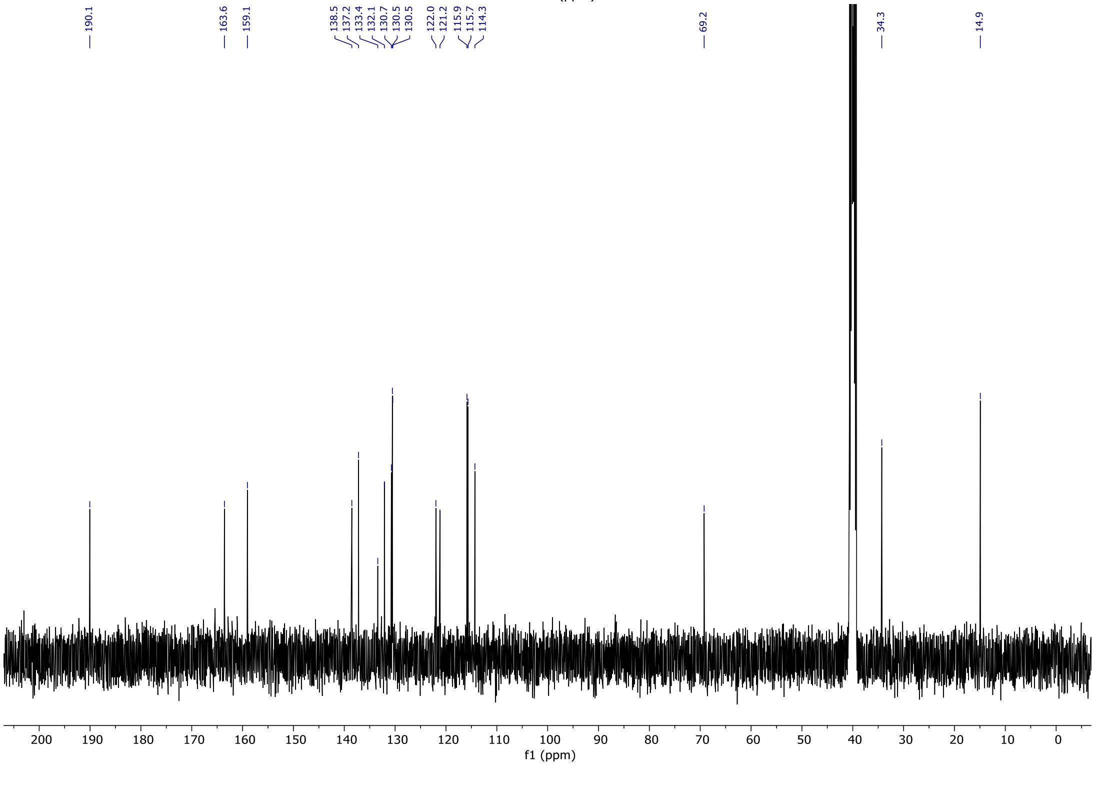

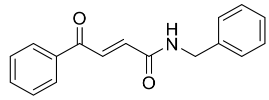

“CAA5”, (E)-N-benzyl-4-oxo-4-phenylbut-2-enamide

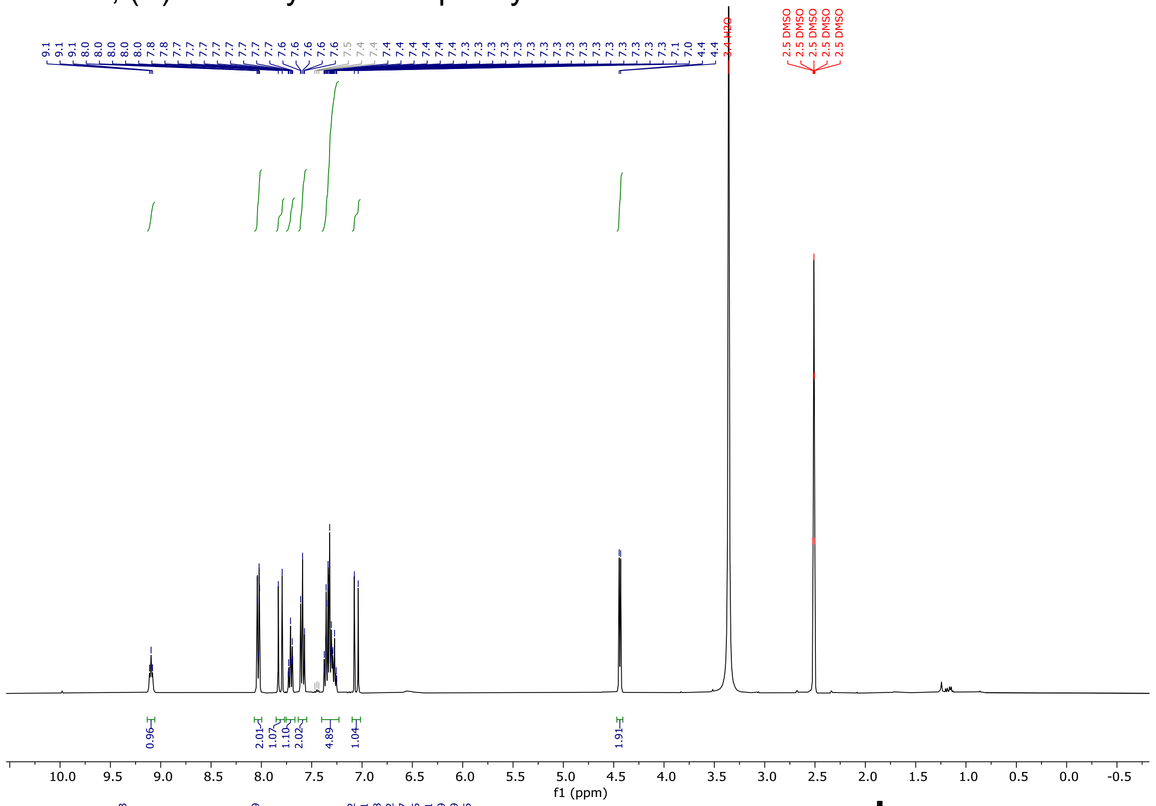

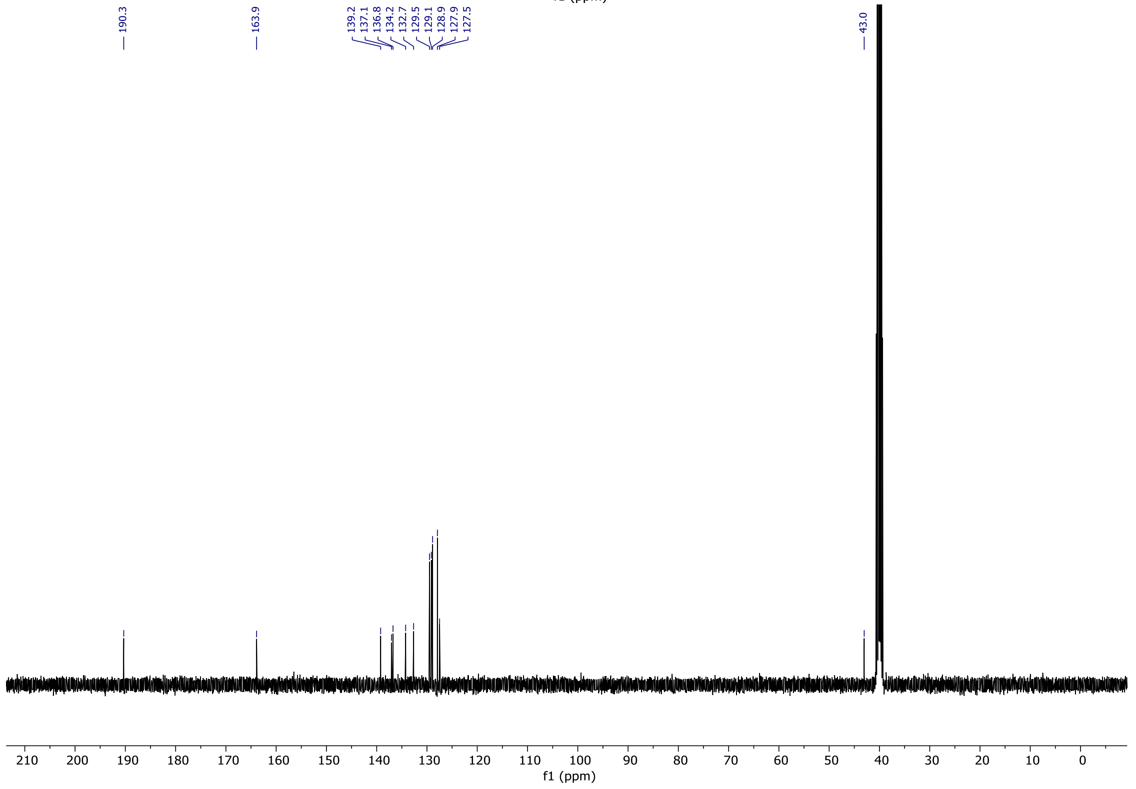

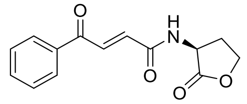

CAA-L, (S,E)-4-oxo-N-(2-oxotetrahydrofuran-3-yl)-4-phenylbut-2-enamide,

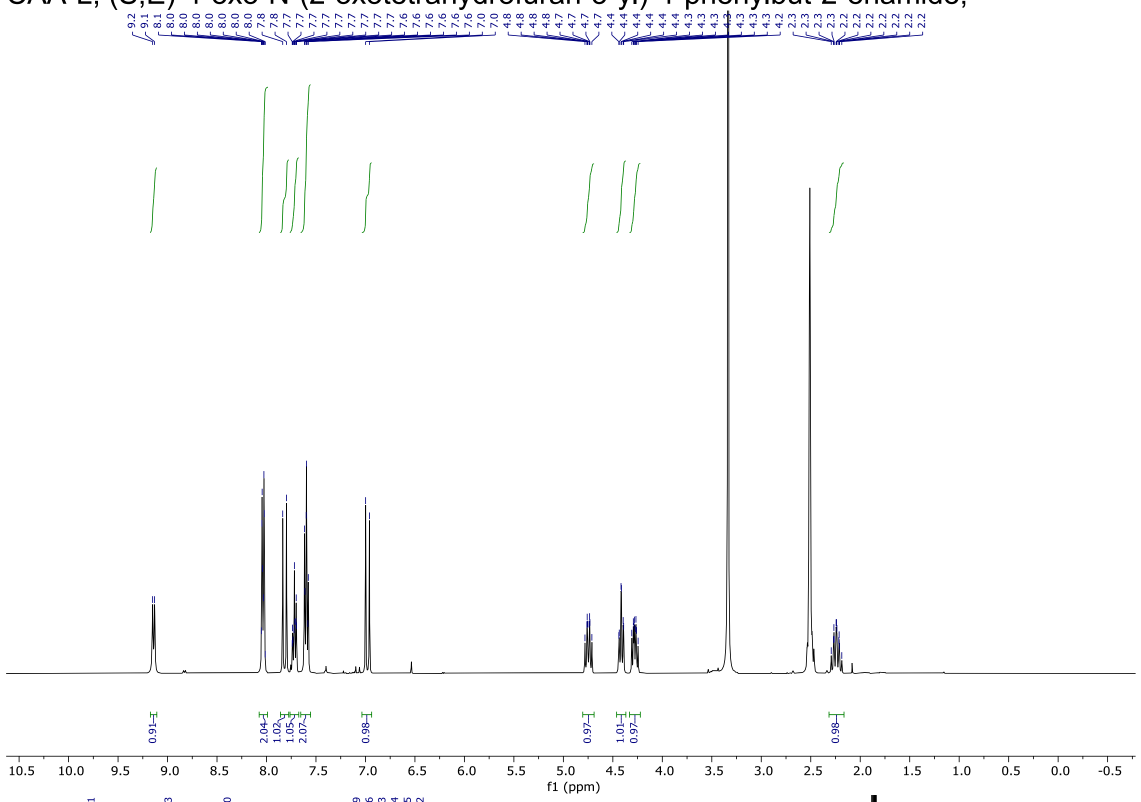

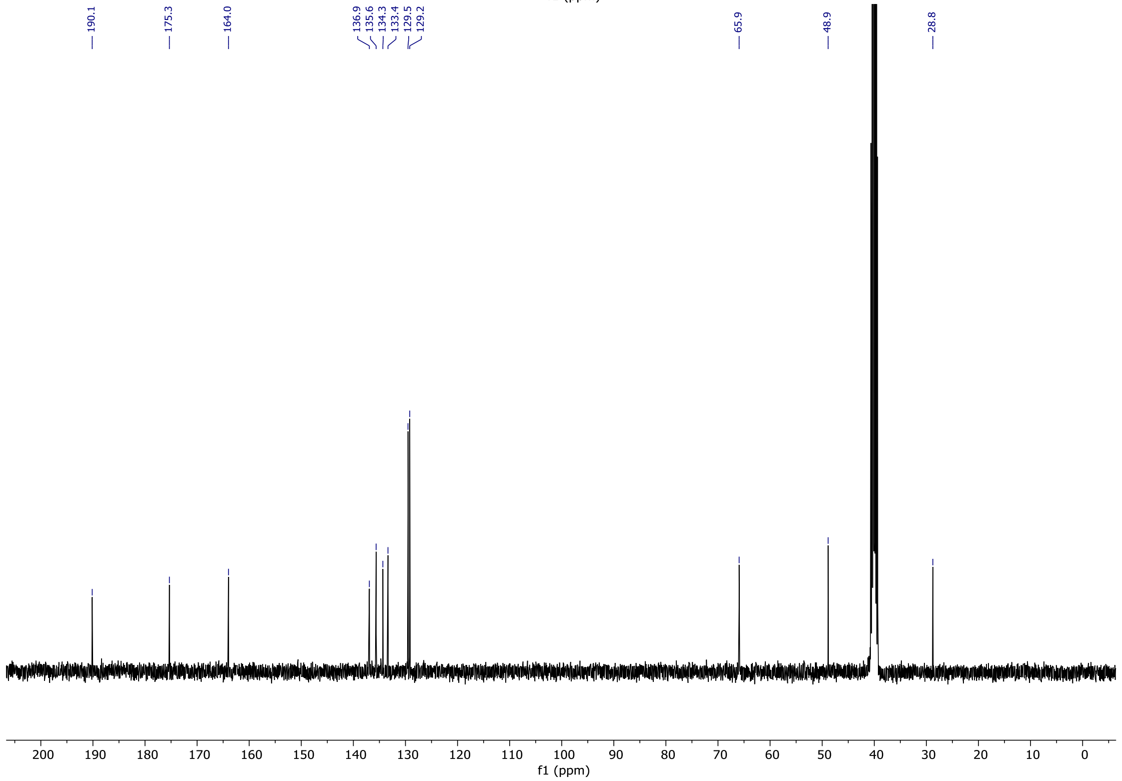

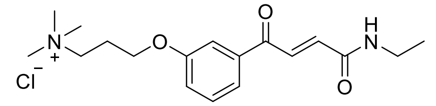

"CAA-Q", (E)-3-(3-(4-(ethylamino)-4-oxobut-2-enoyl)phenoxy)-N,N,N-trimethylpropan-1-aminium chloride

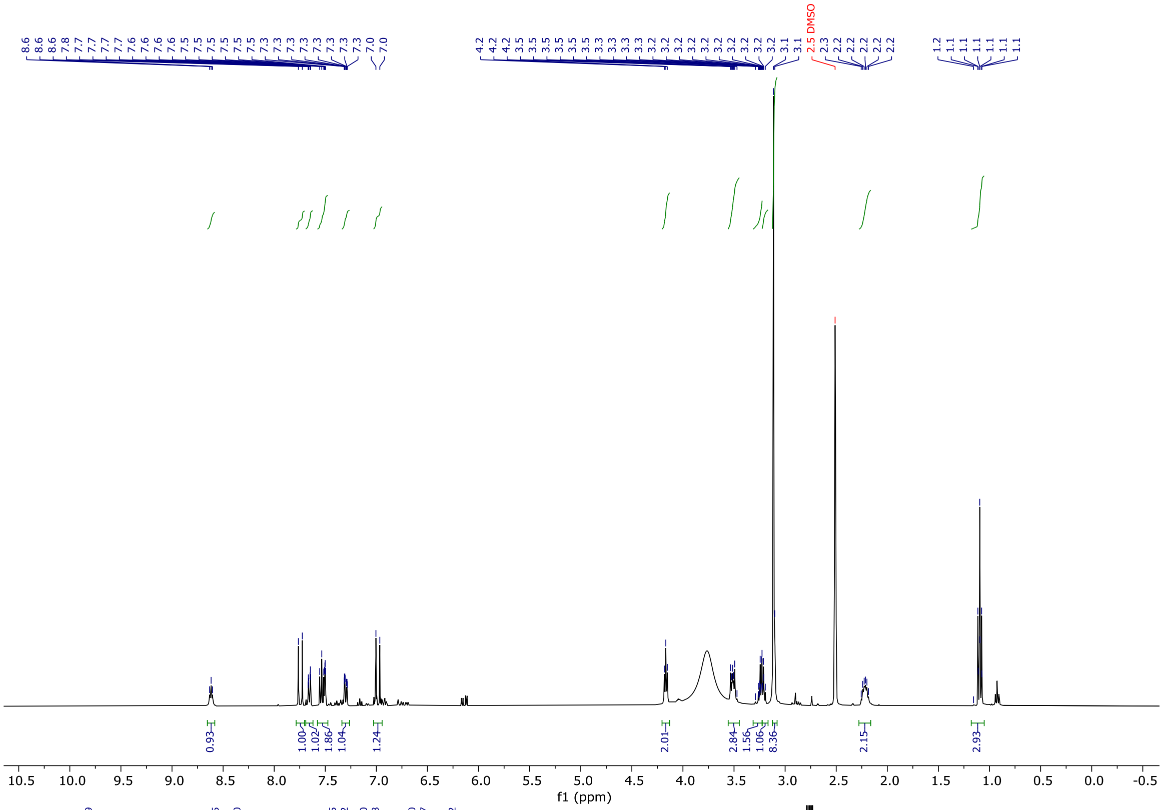

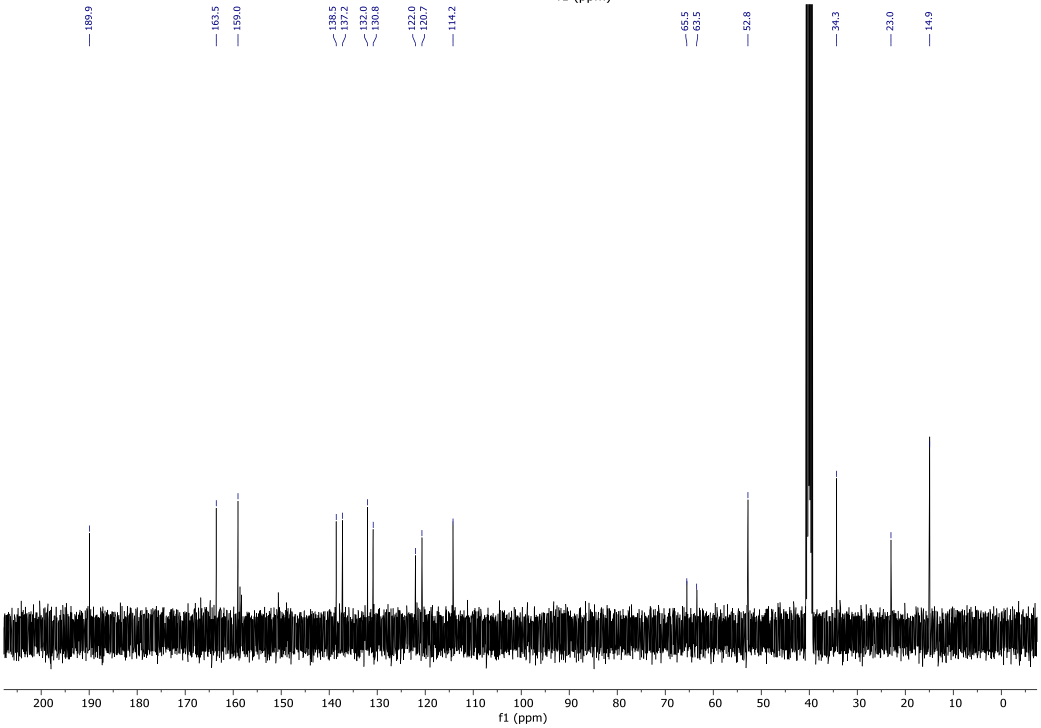

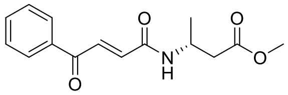

“CAA-A1”, methyl (R,E)-3-(4-oxo-4-phenylbut-2-enamido)butanoate

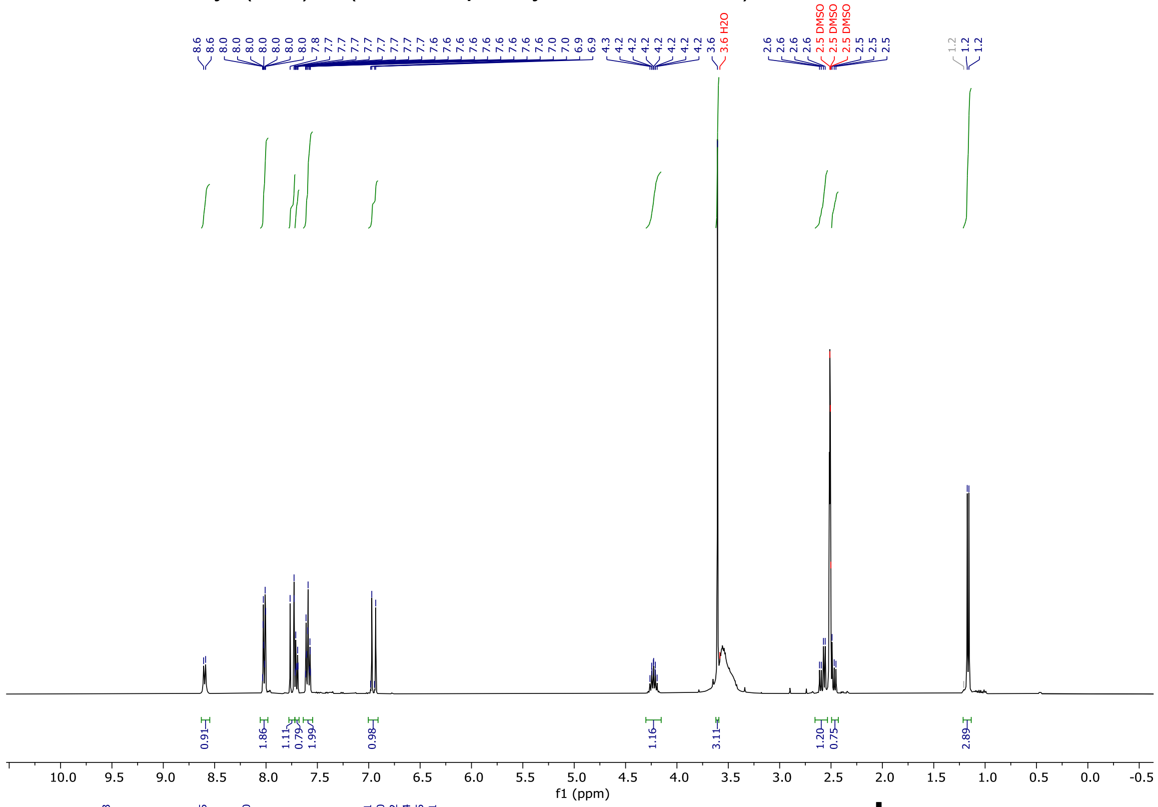

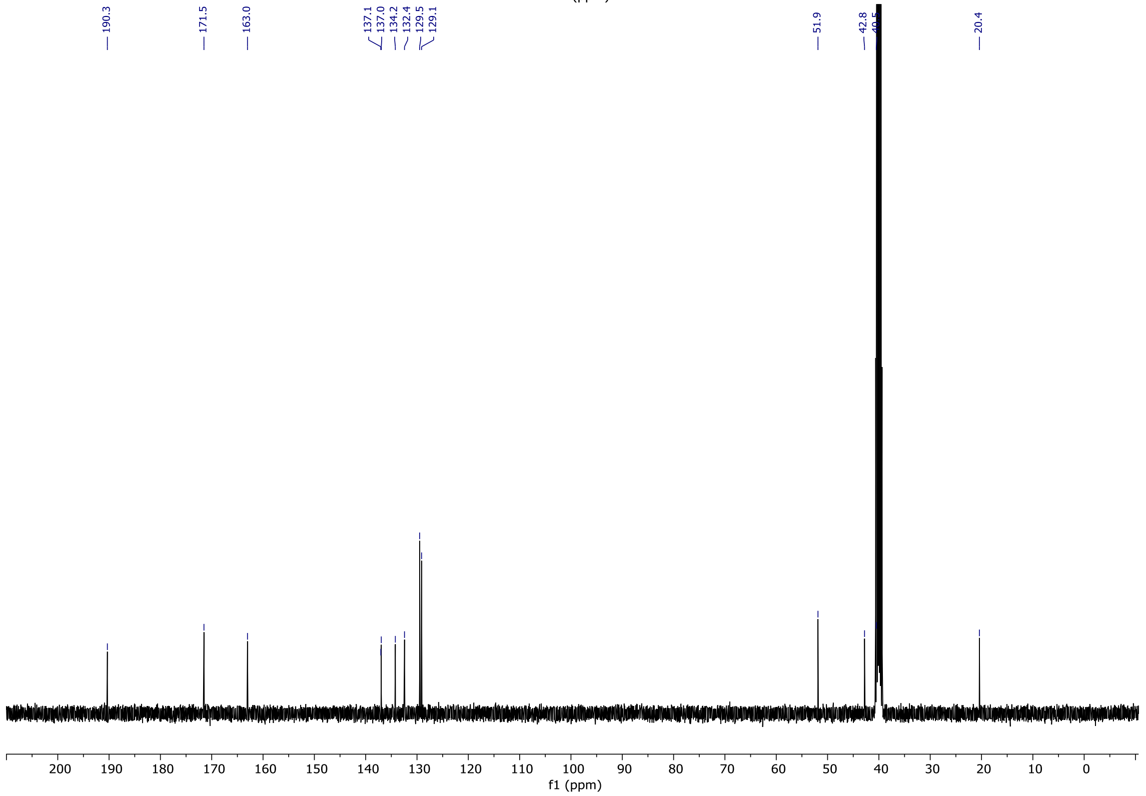

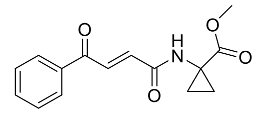

“CAA-A2”, methyl (E)-1-(4-oxo-4-phenylbut-2-enamido)cyclopropane-1-carboxylate,

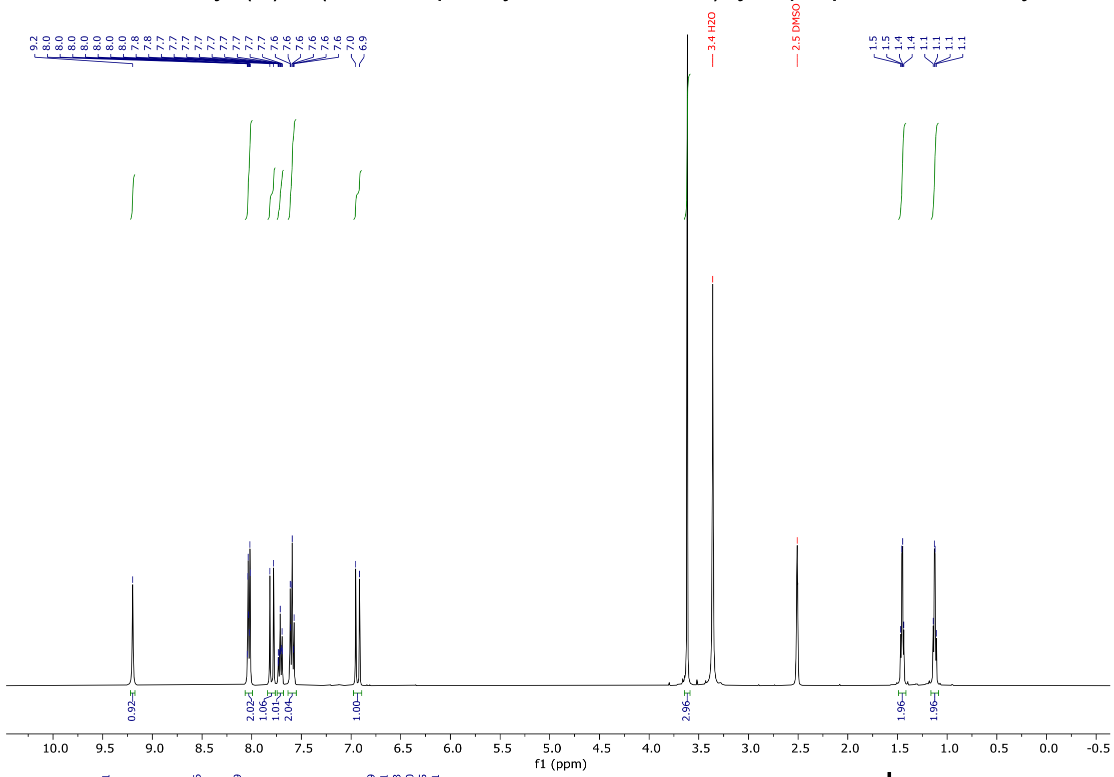

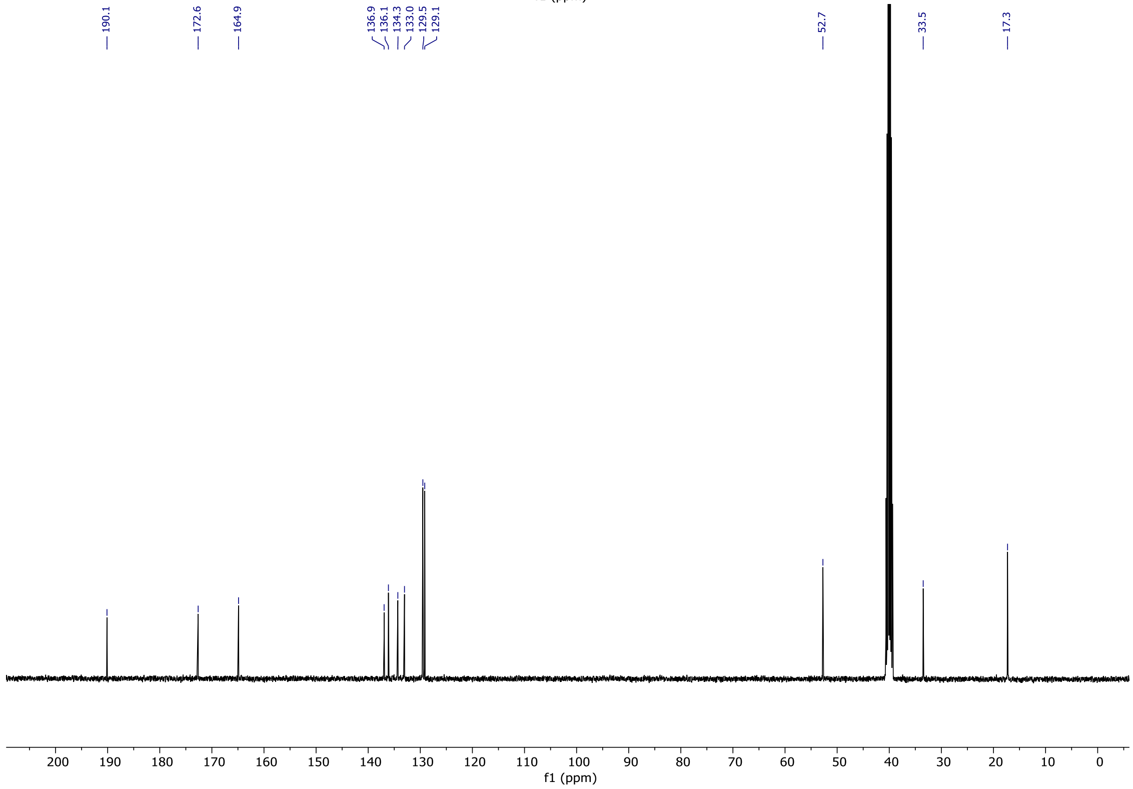

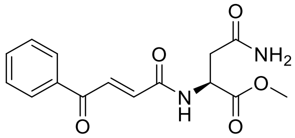

“CAA-A3”, methyl (E)-(4-oxo-4-phenylbut-2-enoyl)-L-asparaginate

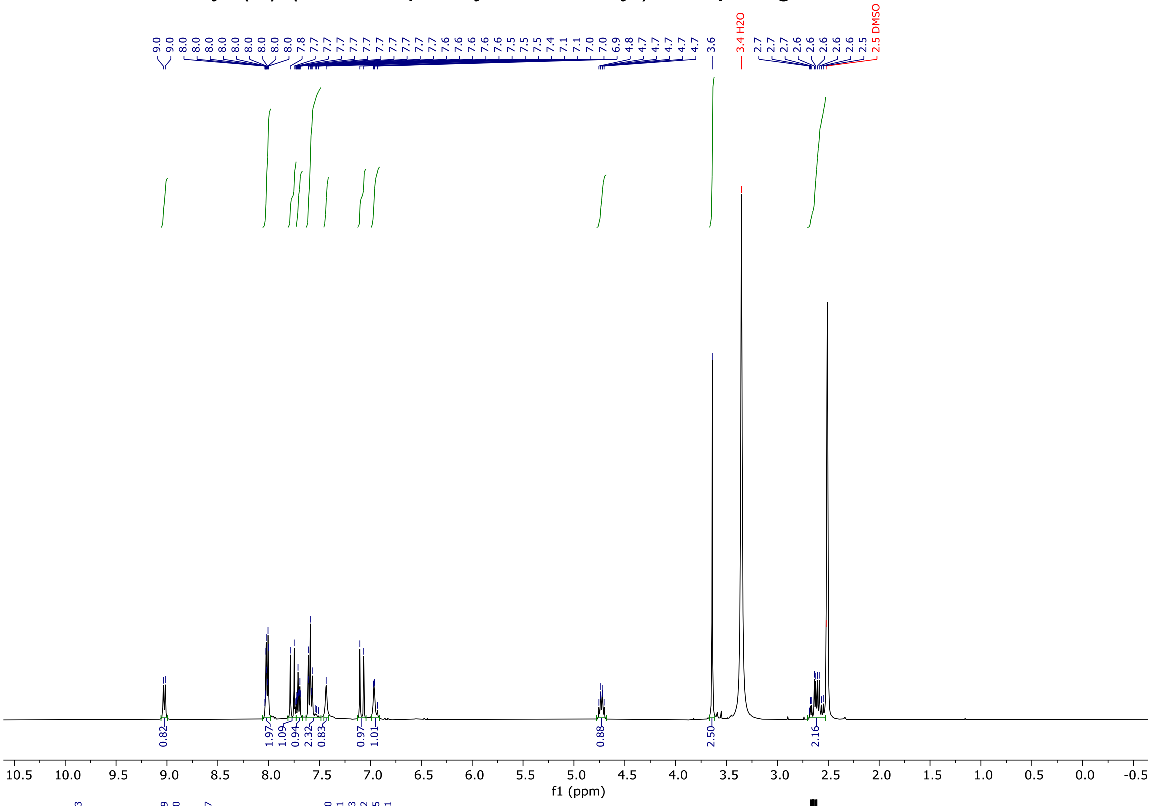

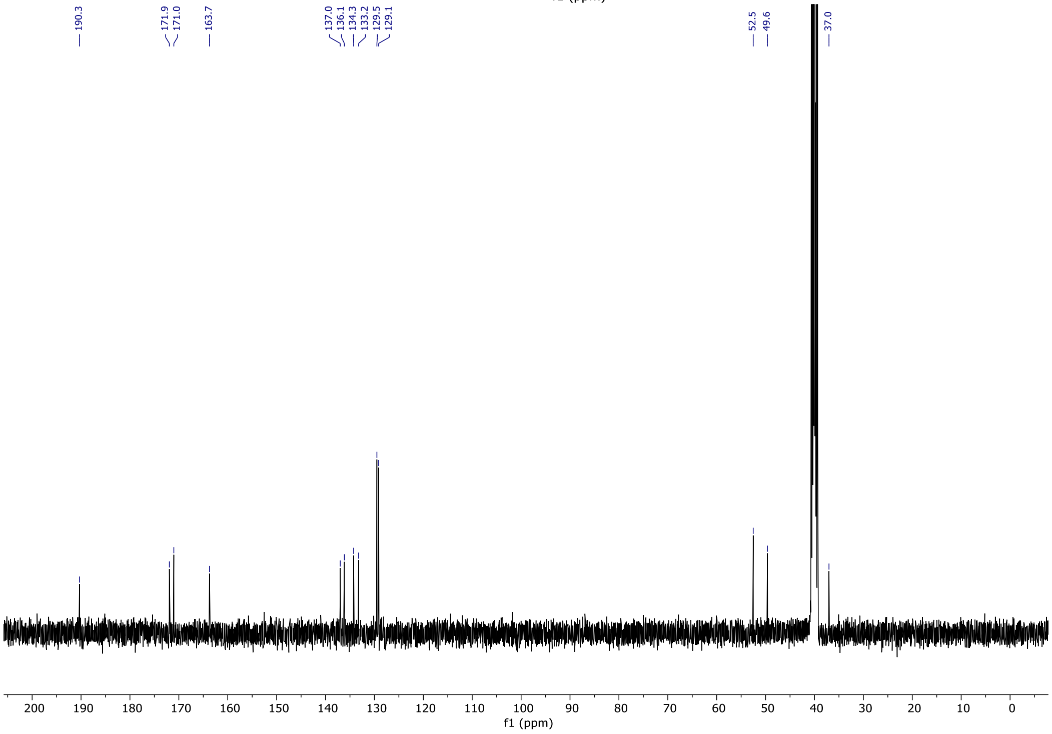

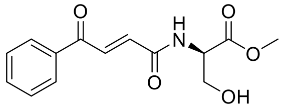

“CAA-A4”, Methyl (E)-(4-oxo-4-phenylbut-2-enoyl)-D-serinate

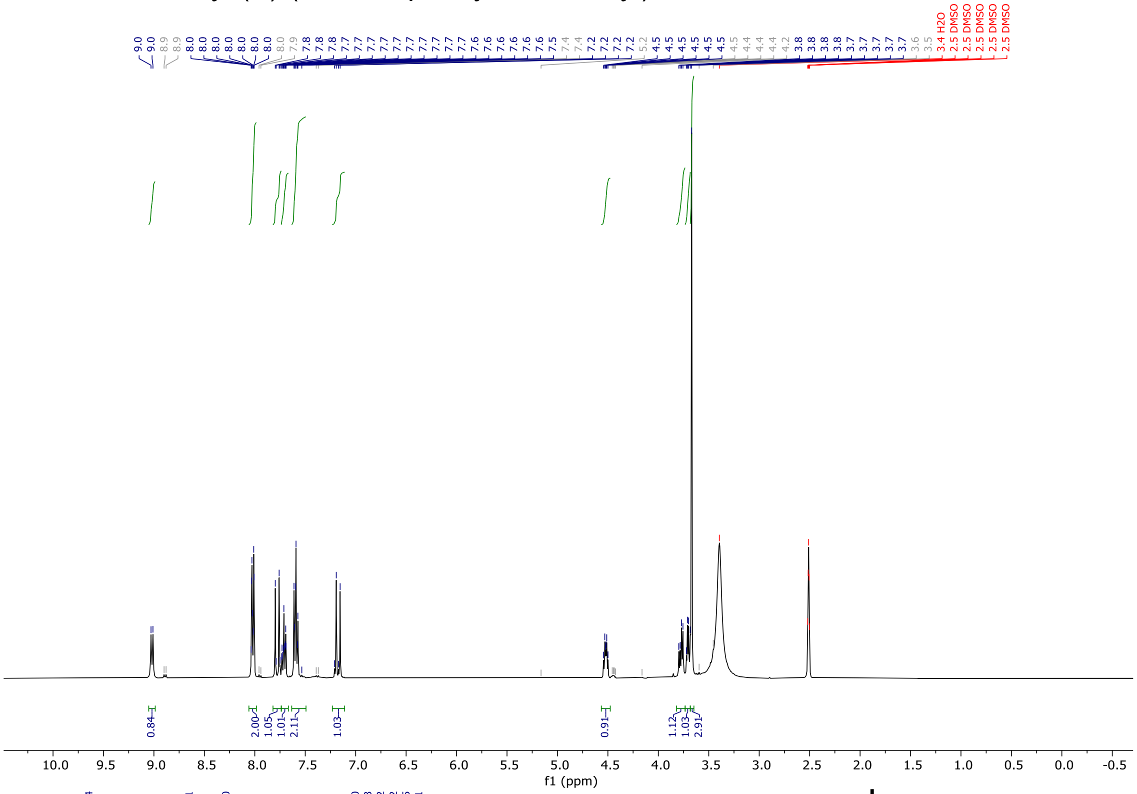

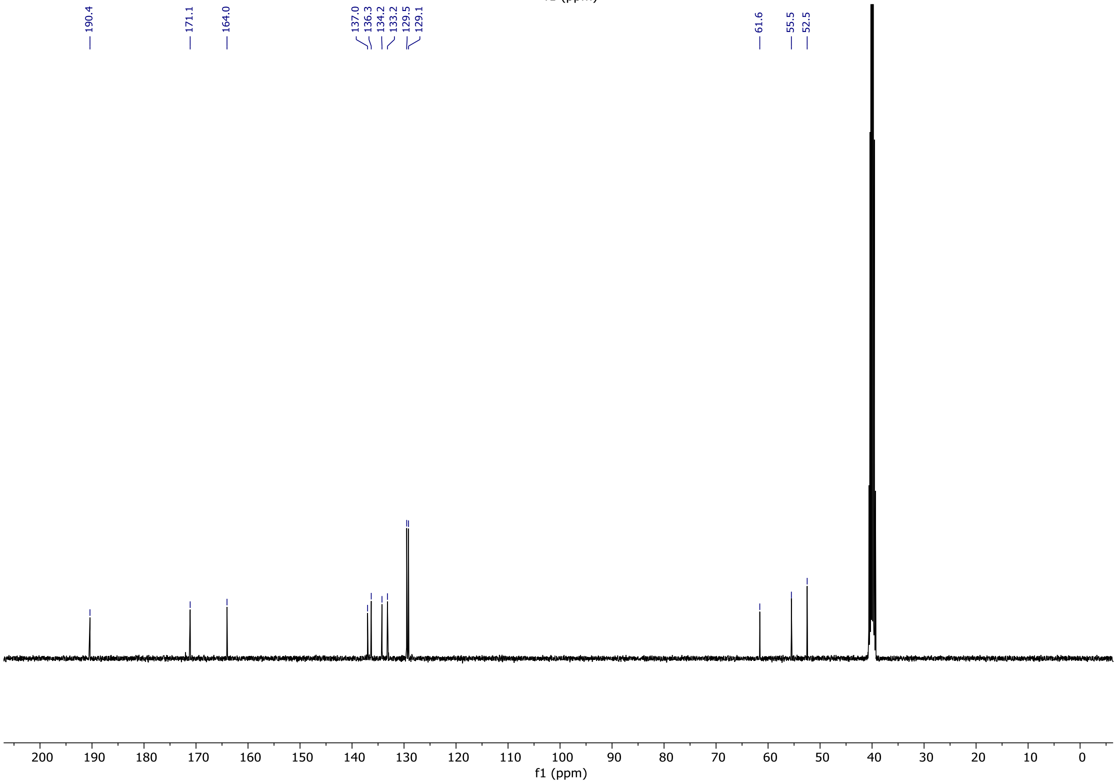

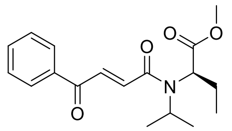

“CAA-A5”, methyl (R,E)-2-(N-isopropyl-4-oxo-4-phenylbut-2-enamido)butanoate

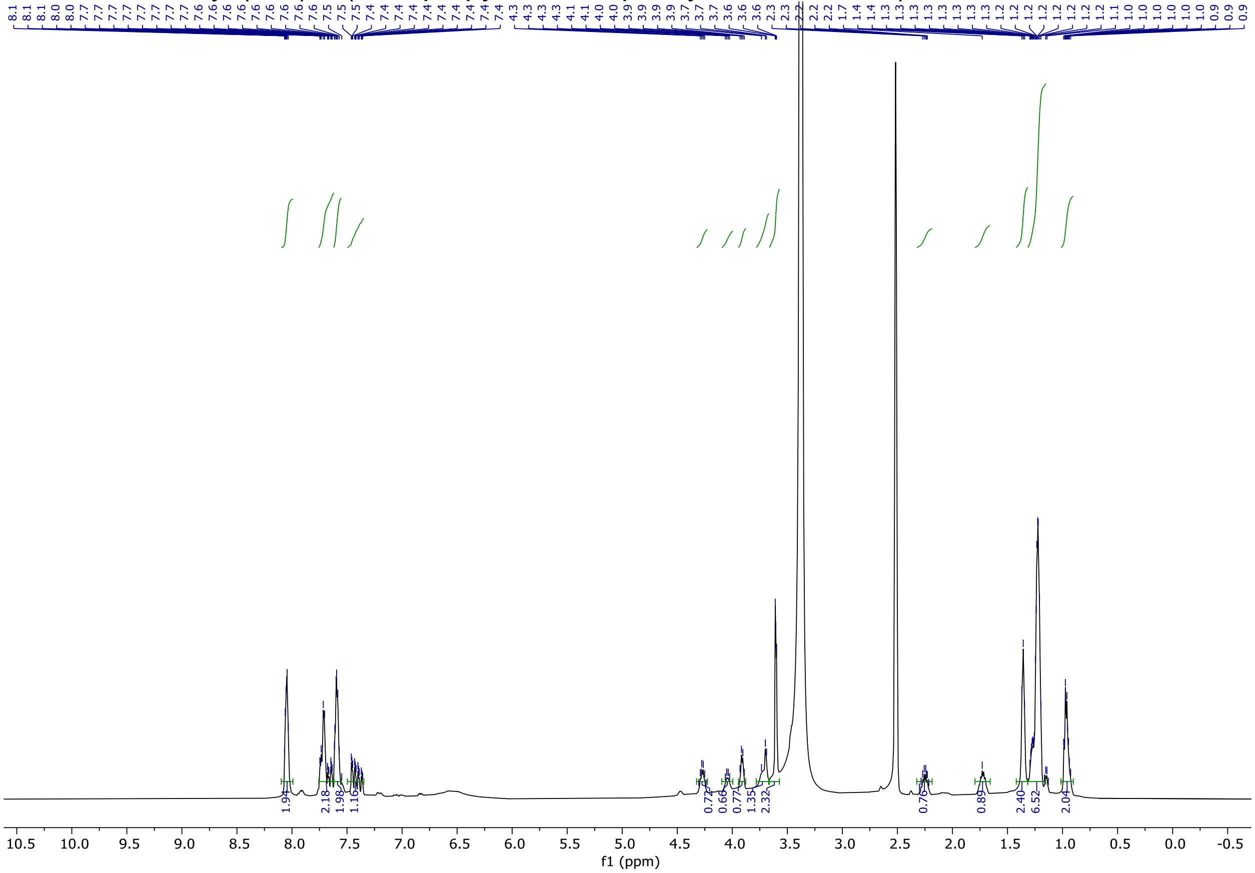

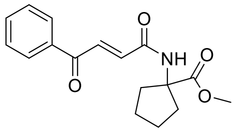

"CAA-A6", methyl (E)-1-(4-oxo-4-phenylbut-2-enamido)cyclopentane-1-carboxylate

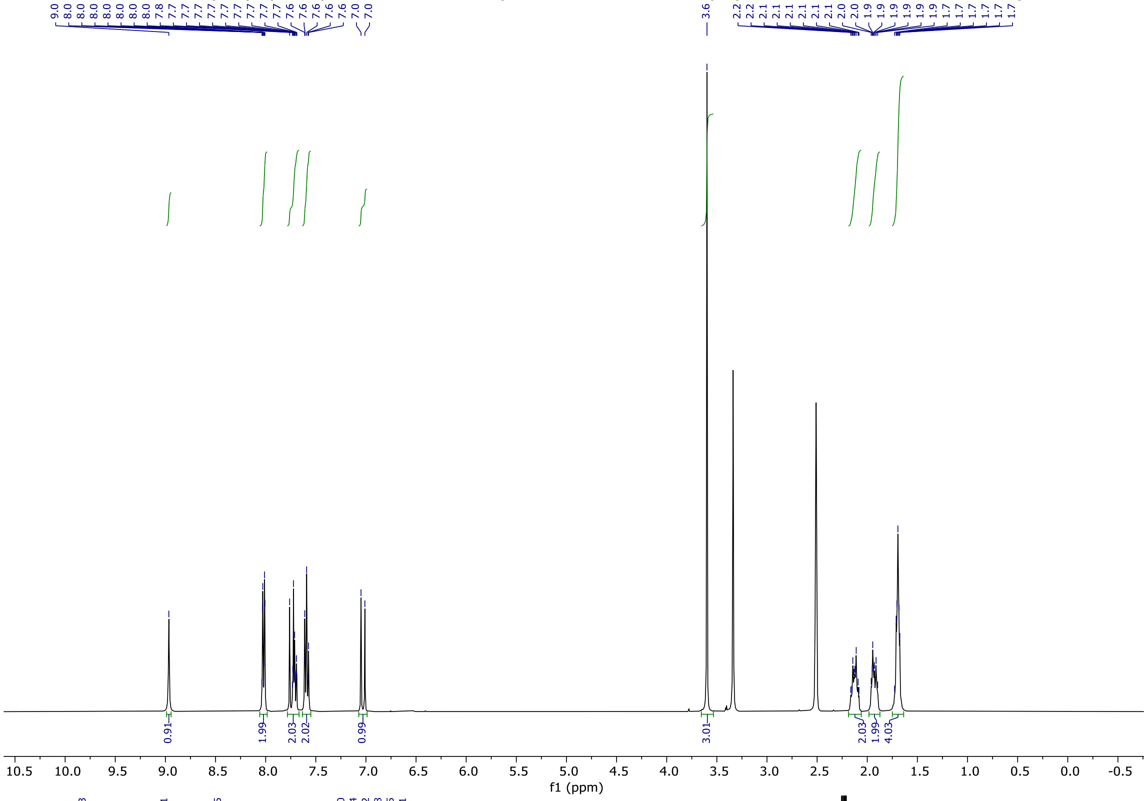

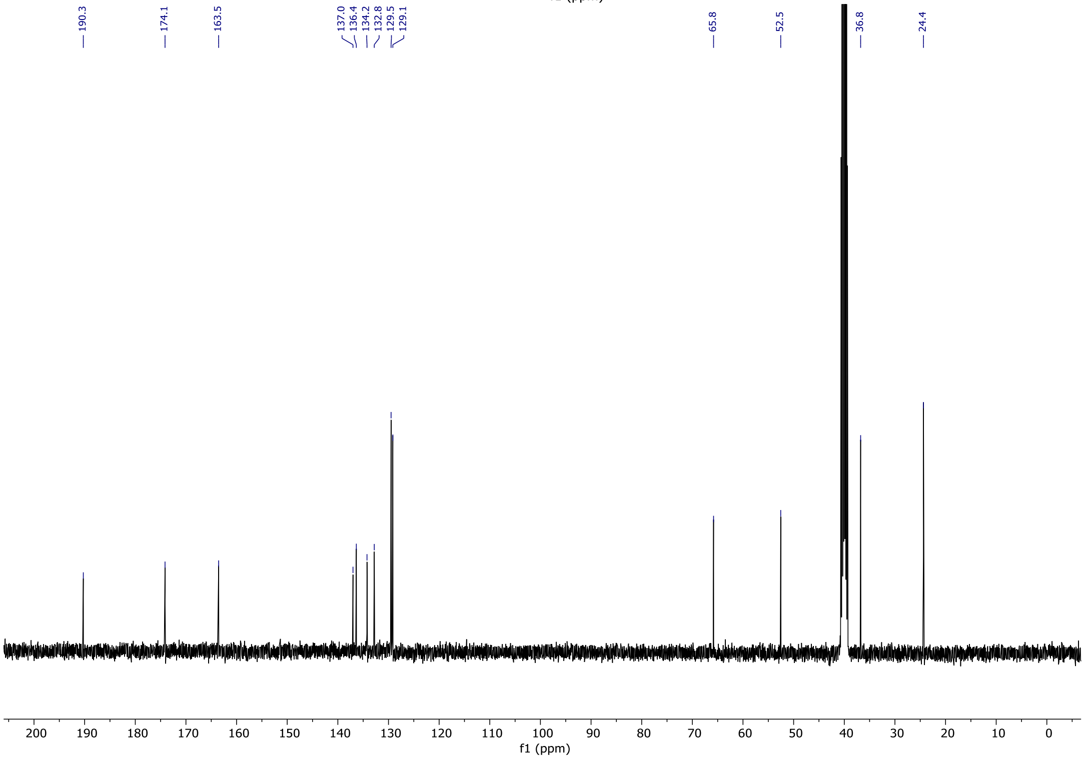

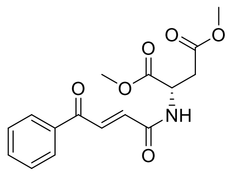

“CAA-AA”, dimethyl (E)-(4-oxo-4-phenylbut-2-enoyl)-L-aspartate

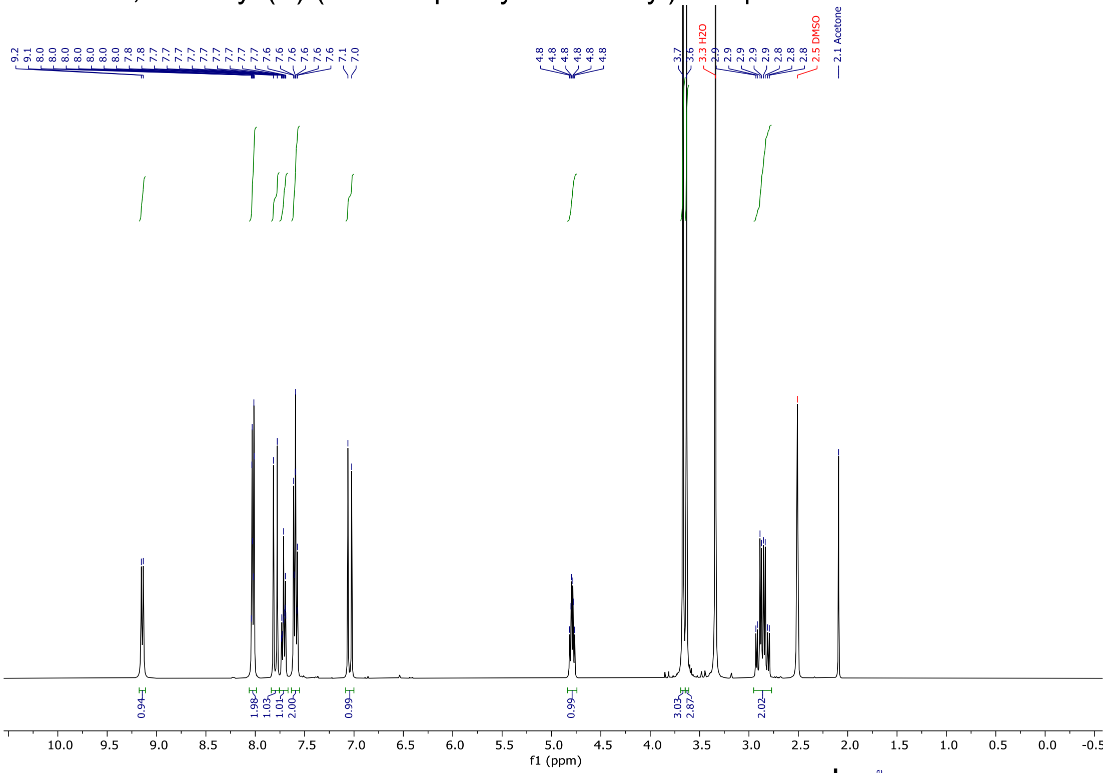

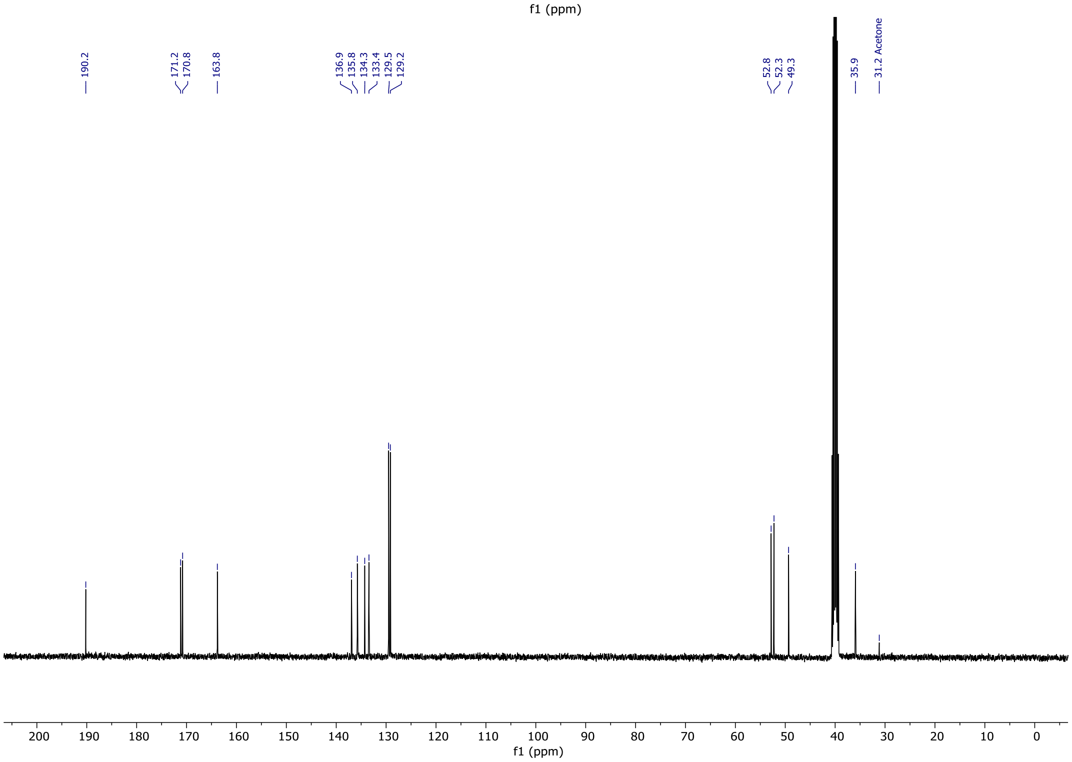

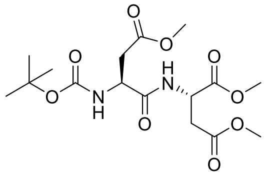

dimethyl ((S)-2-((tert-butoxycarbonyl)amino)-4-methoxy-4-oxobutanoyl)-L-aspartate

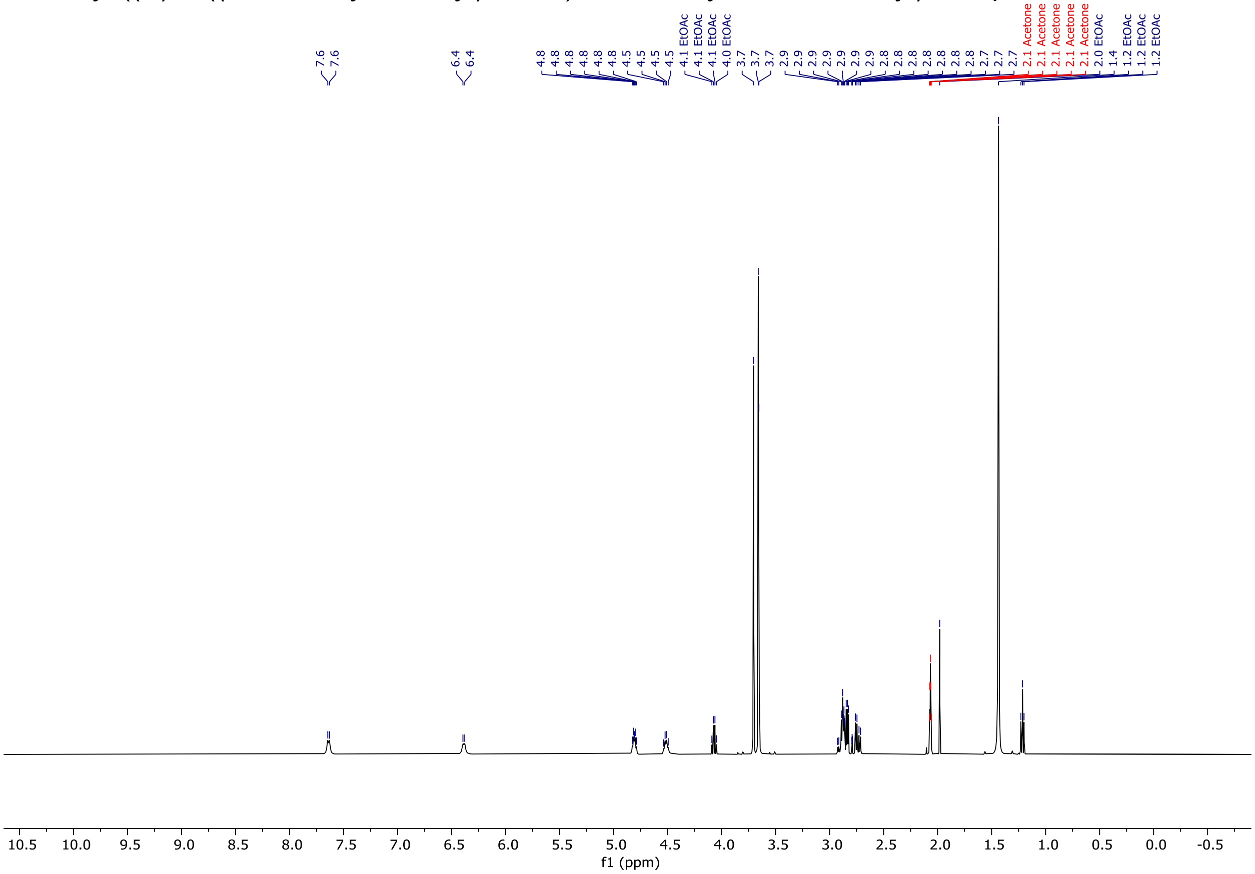

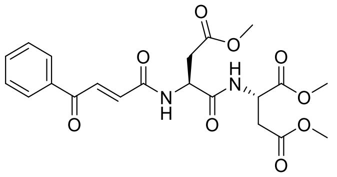

“CAA-Asp$_2$”, dimethyl ((S)-4-methoxy-4-oxo-2-((E)-4-oxo-4-phenylbut-2-enamido)butanoyl)-L-aspartate

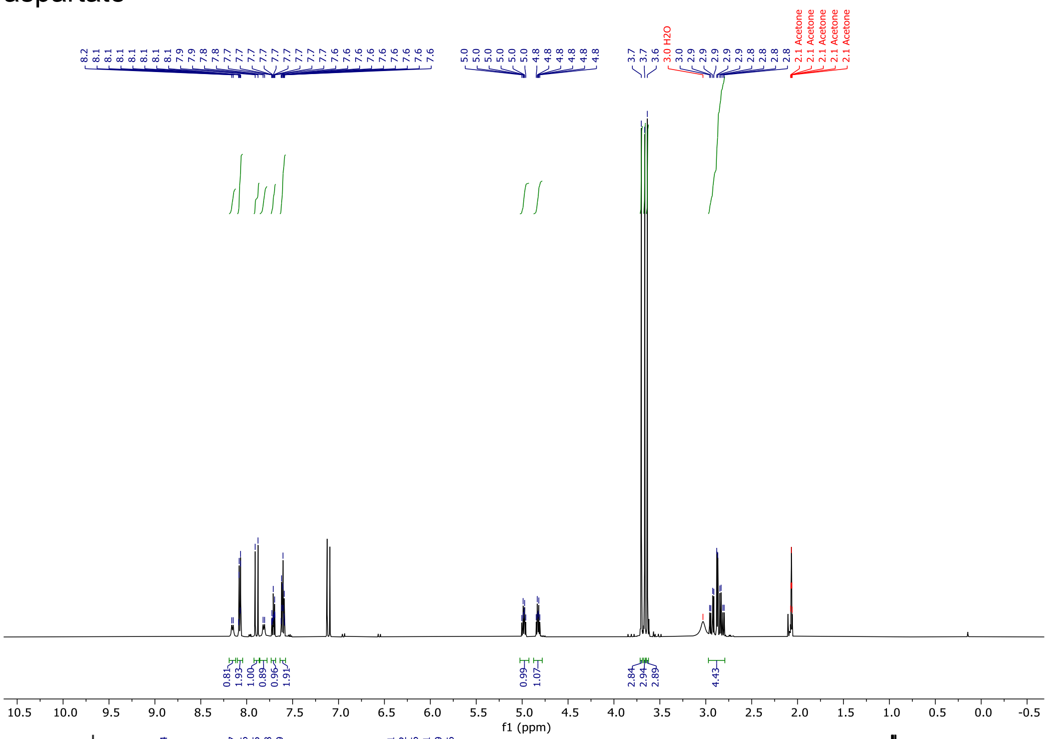

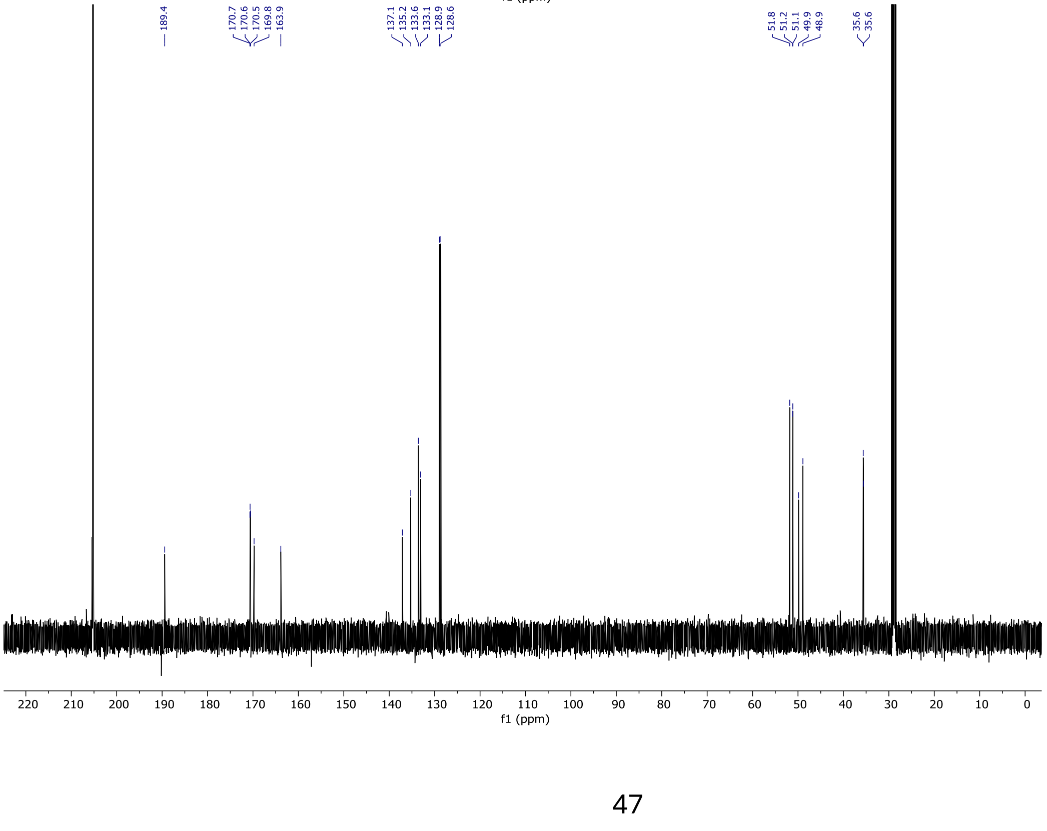

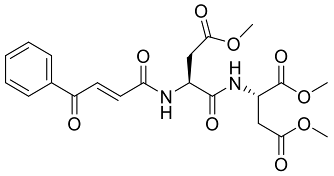

COSY

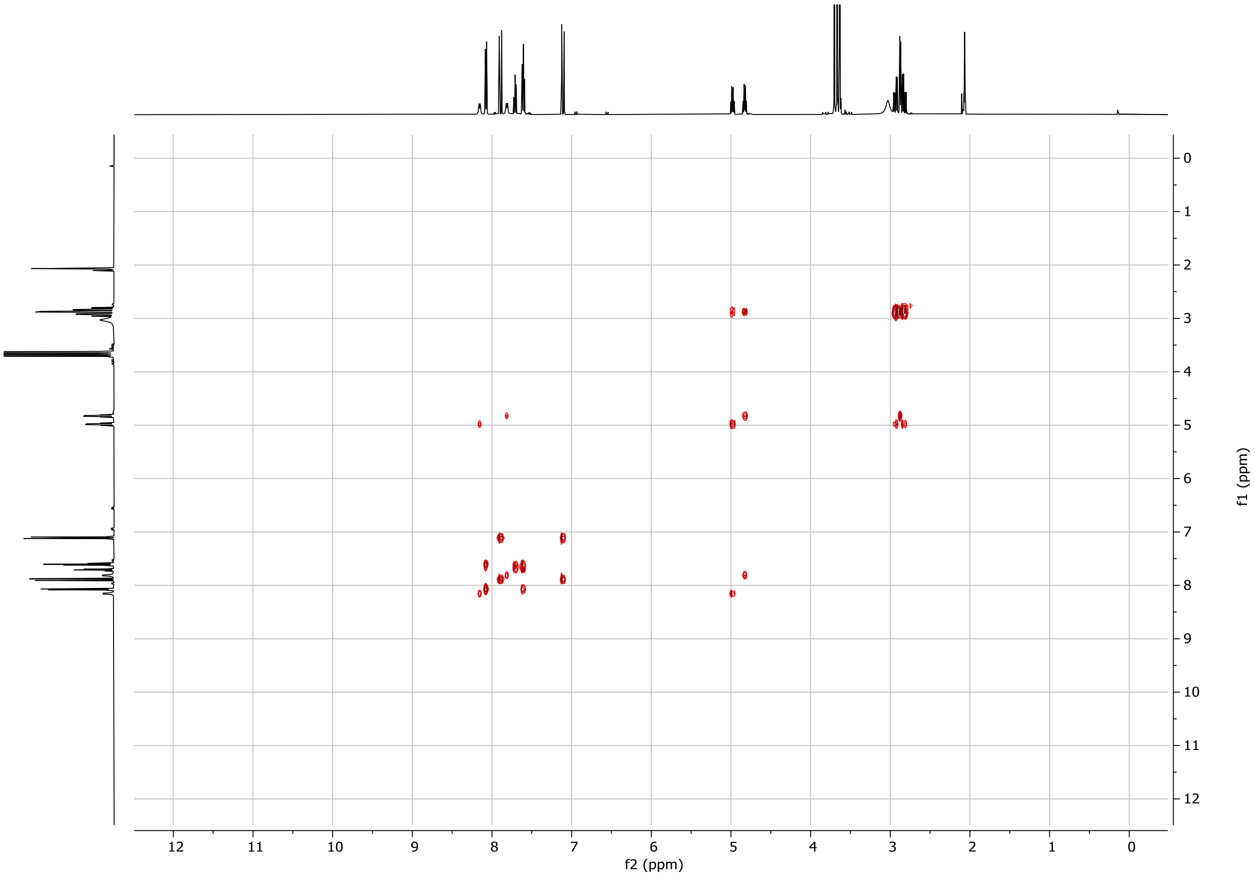

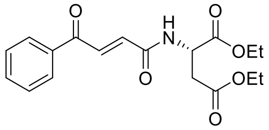

"CAA-AAR", diethyl (E)-(4-oxo-4-phenylbut-2-enoyl)-L-aspartate

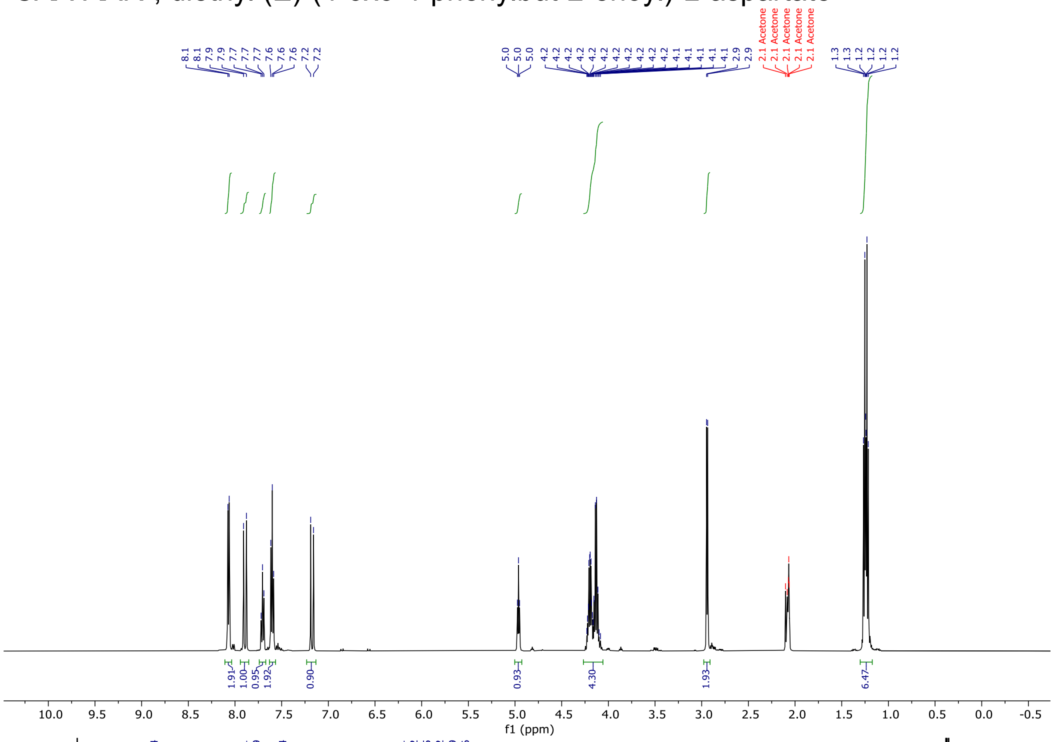

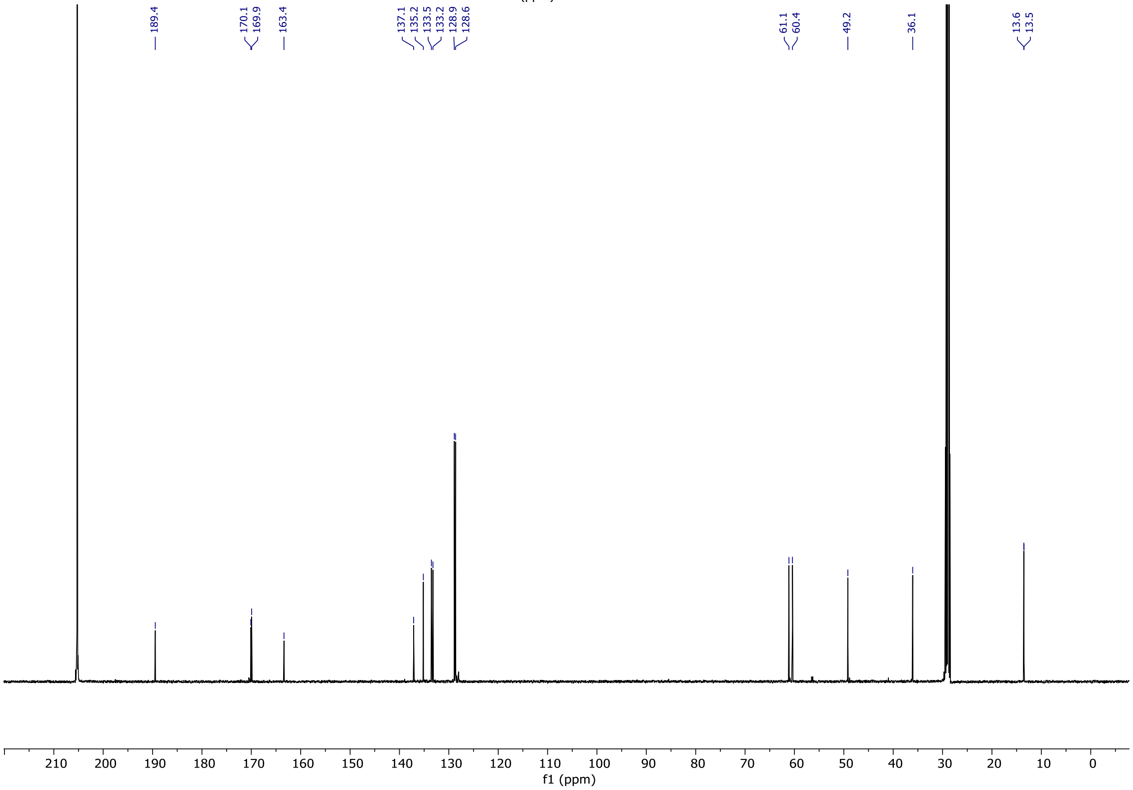

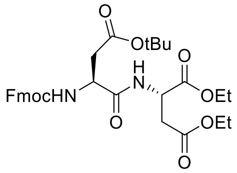

diethyl ((S)-2-((((9H-fluoren-9-yl)methoxy)carbonyl)amino)-4-(tert-butoxy)-4-oxobutanoyl)-L-aspartate

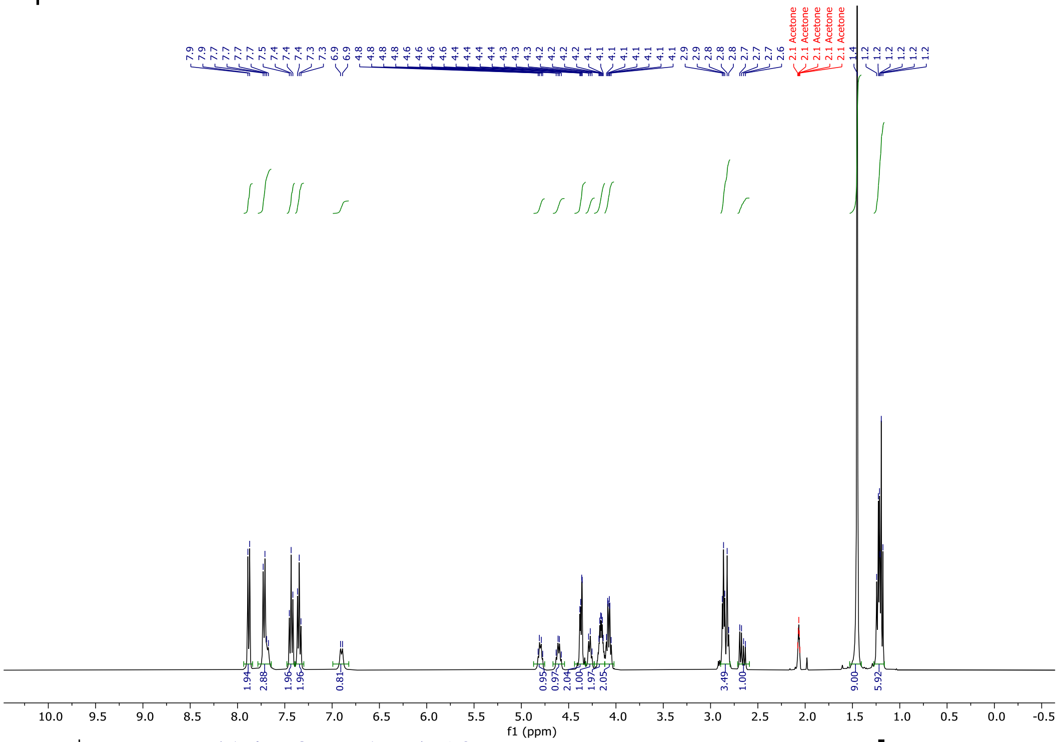

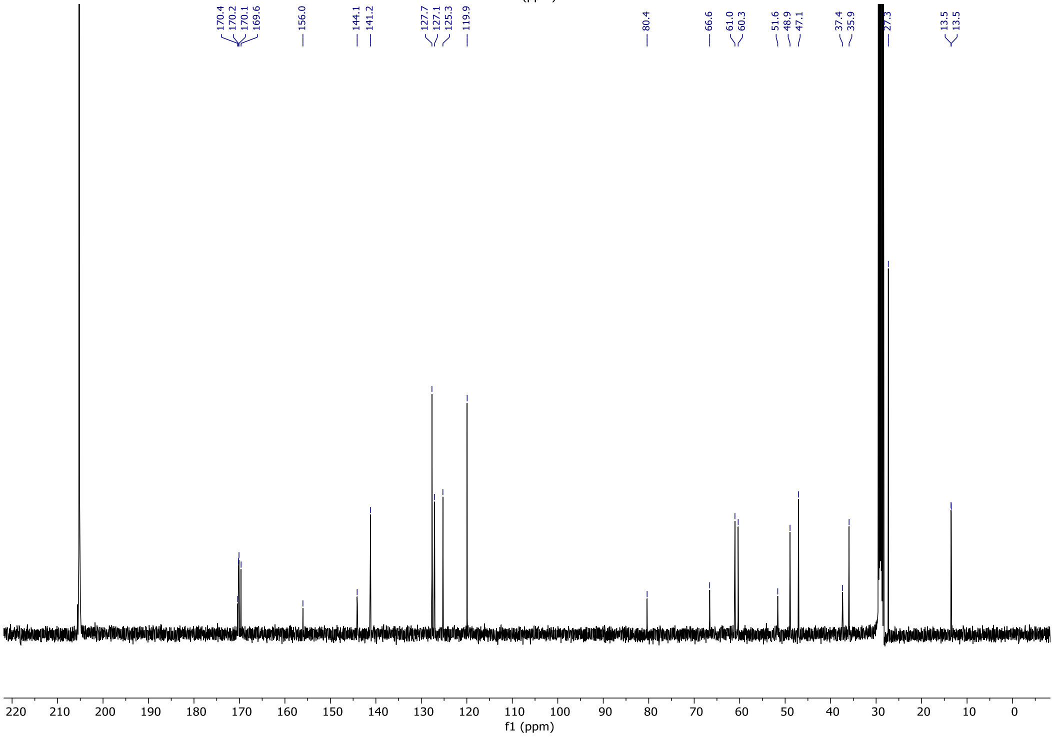

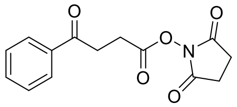

2,5-dioxopyrrolidin-1-yl 4-oxo-4-phenylbutanoate

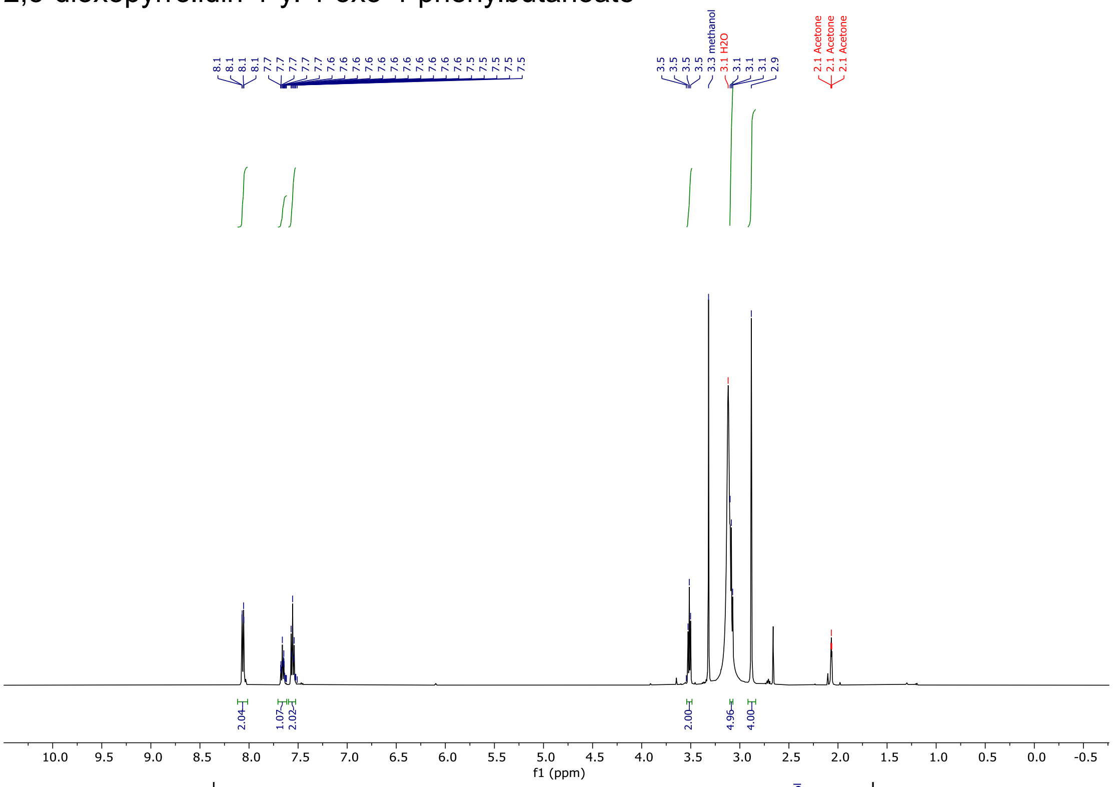

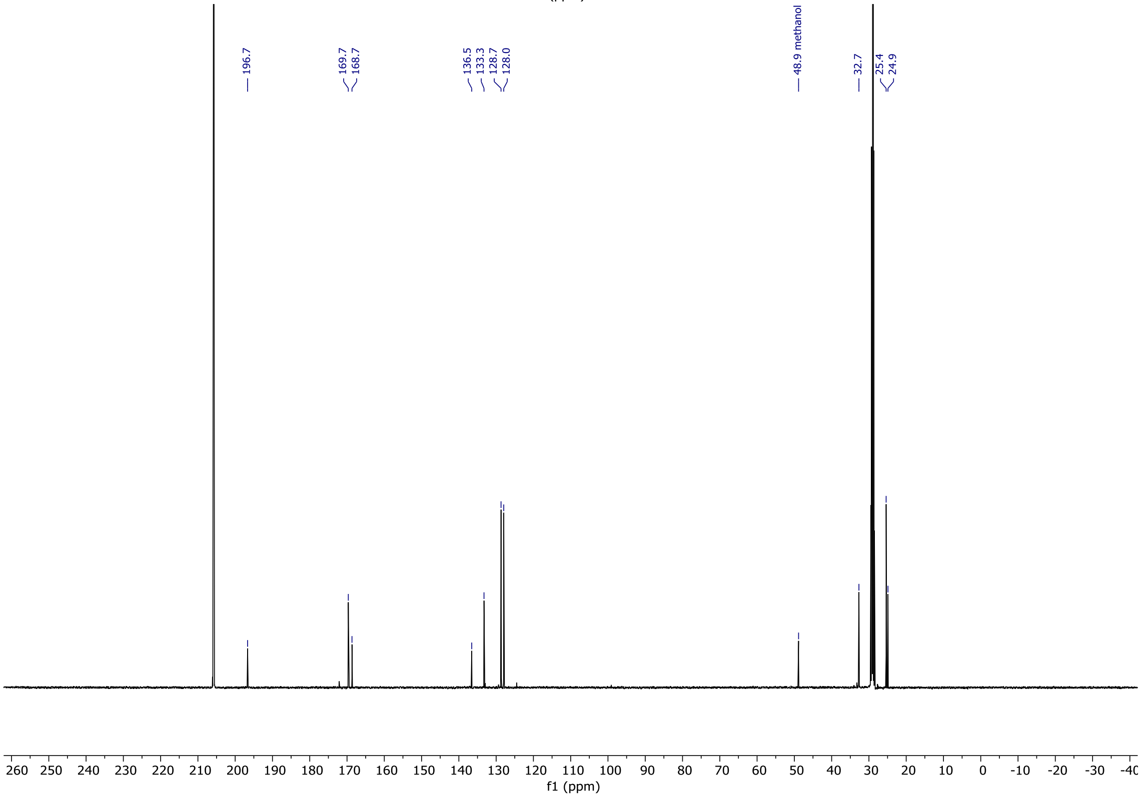

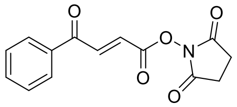

2,5-dioxopyrrolidin-1-yl (E)-4-oxo-4-phenylbut-2-enoate

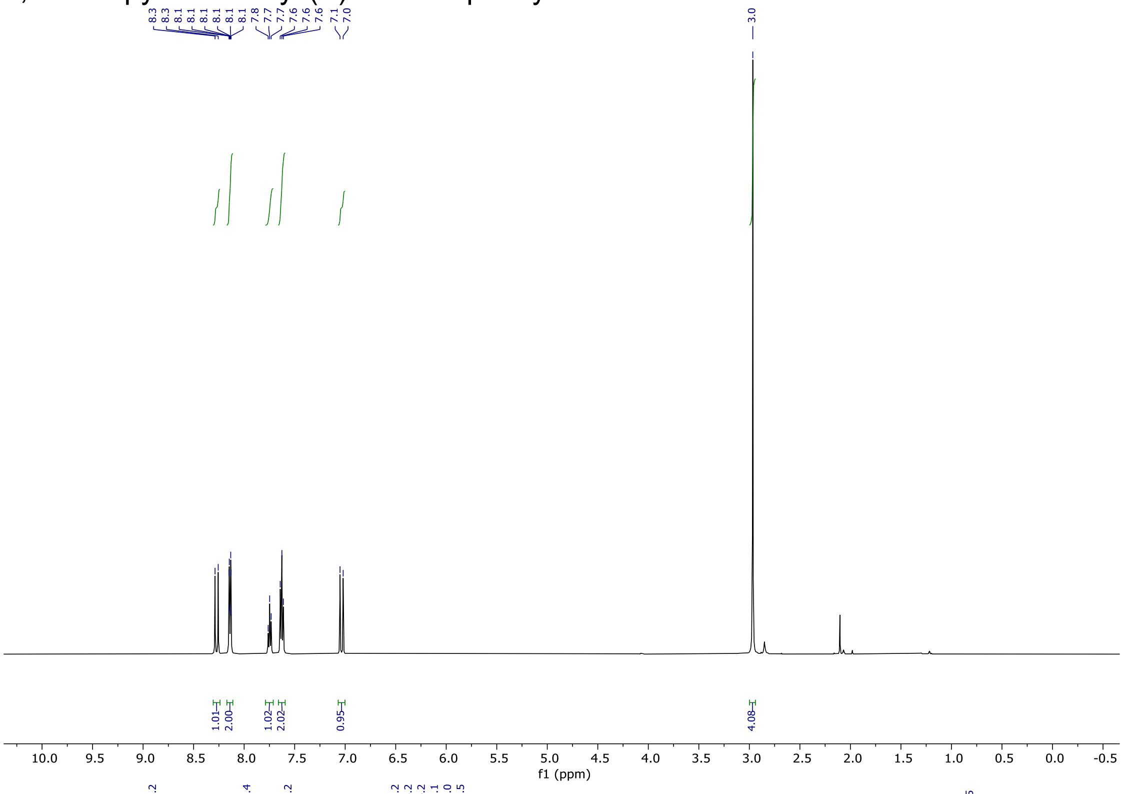

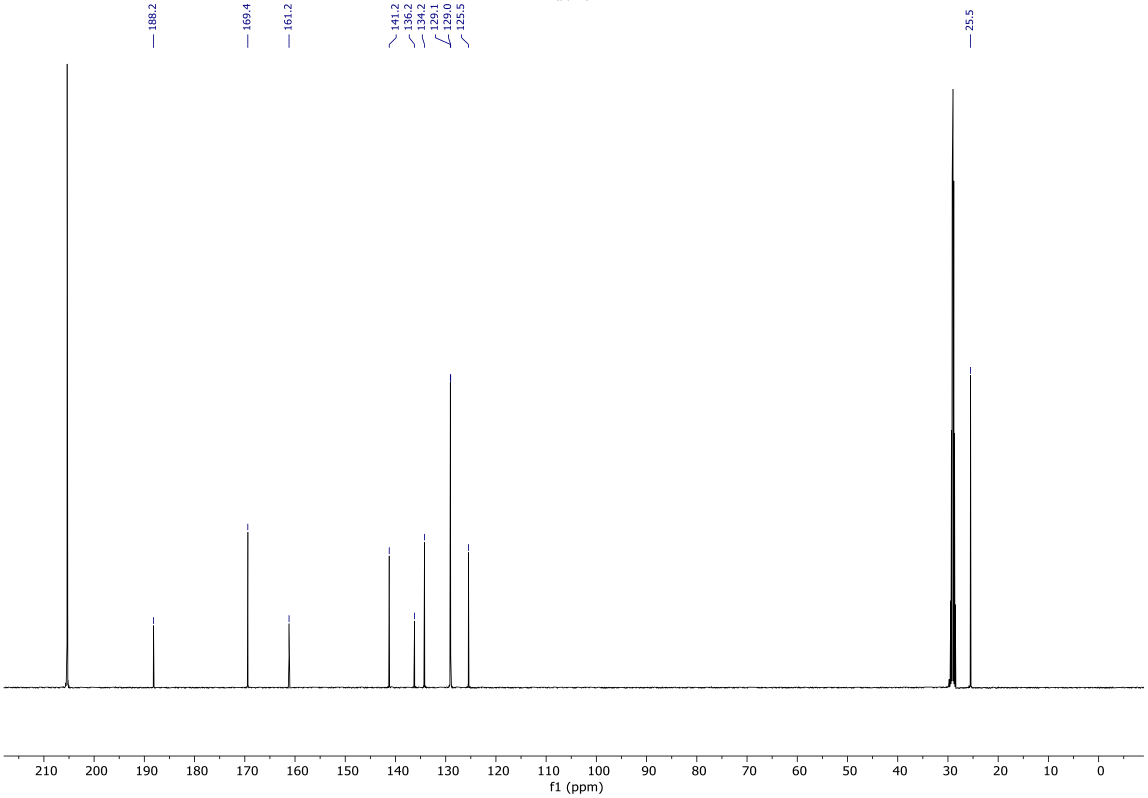

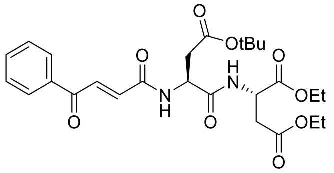

“CAA-Asp$_2$R”, diethyl ((S)-4-(tert-butoxy)-4-oxo-2-((E)-4-oxo-4-phenylbut-2-enamido)butanoyl)-L-aspartate

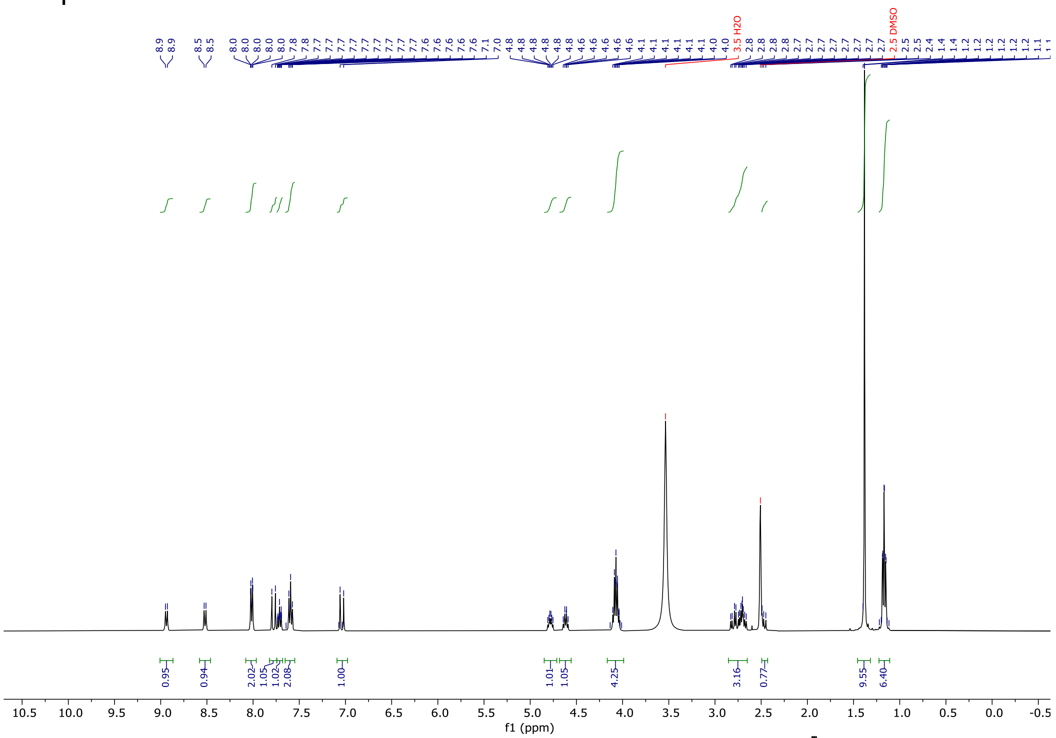

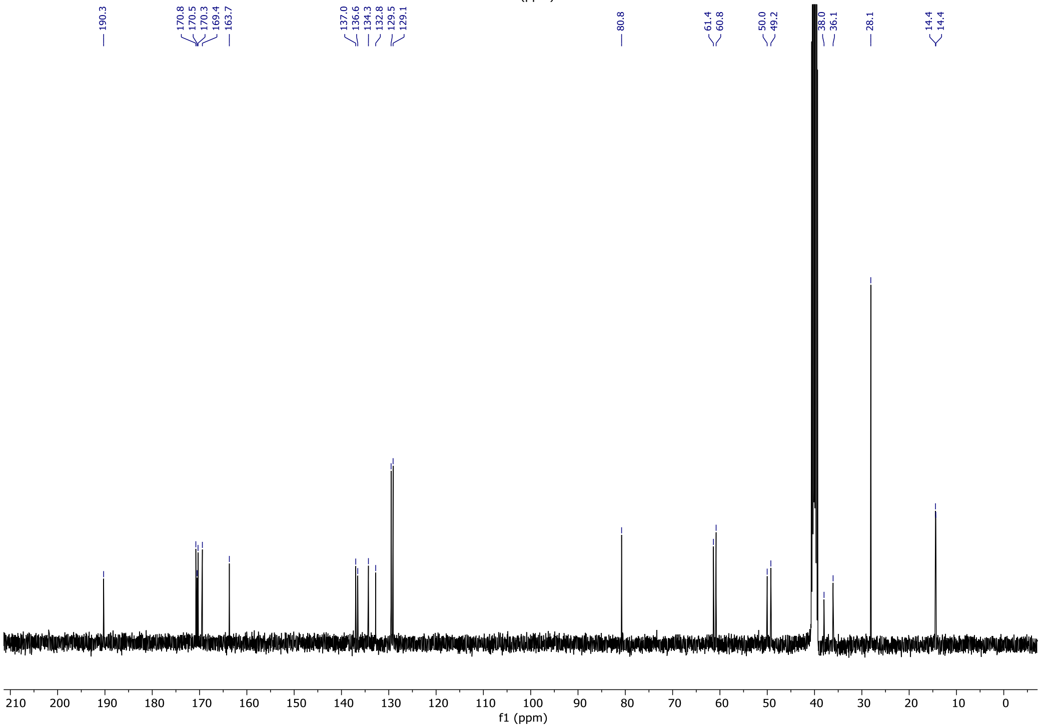

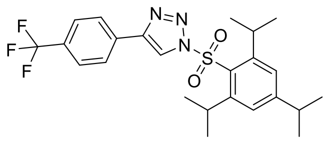

“AHL-003”, 4-(4-(trifluoromethyl)phenyl)-1-((2,4,6-triisopropylphenyl)sulfonyl)-1H-1,2,3-triazole

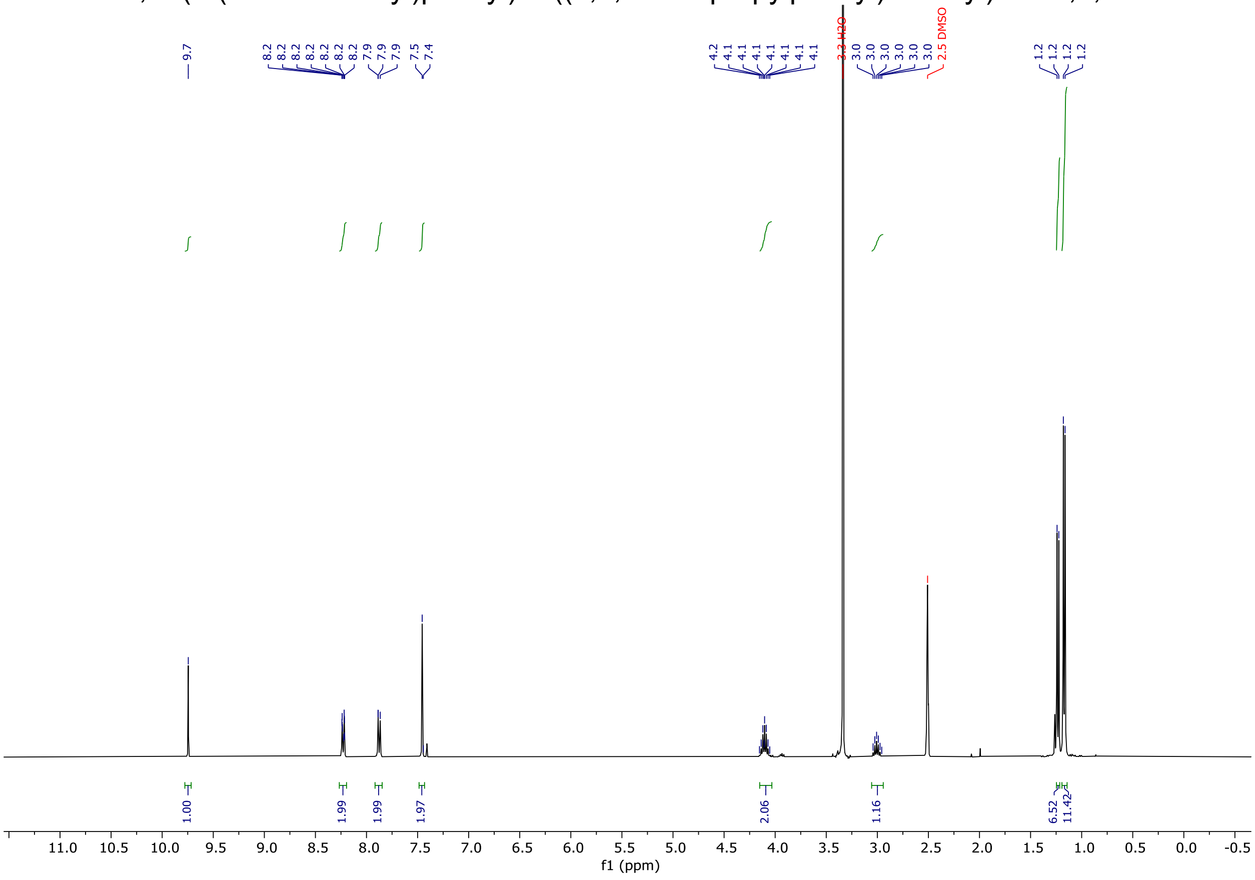

References

1 Ran, F. A. *et al.* Genome engineering using the CRISPR-Cas9 system. *Nat Protoc* **8**, 2281-2308, doi:10.1038/nprot.2013.143 (2013).

2 Kedersha, N. *et al.* G3BP-Caprin1-USP10 complexes mediate stress granule condensation and associate with 40S subunits. *J Cell Biol* **212**, 845-860, doi:10.1083/jcb.201508028 (2016).

3 Ding, S. *et al.* Efficient transposition of the piggyBac (PB) transposon in mammalian cells and mice. *Cell* **122**, 473-483, doi:10.1016/j.cell.2005.07.013 (2005).

4 Bhardwaj, G. *et al.* Accurate de novo design of hyperstable constrained peptides. *Nature* **538**, 329-335, doi:10.1038/nature19791 (2016).

5 Bell, E. W., Brown, B. P. & Meiler, J. FakeRotLib: expedient non-canonical amino acid parameterization in Rosetta. *bioRxiv*, doi:10.1101/2025.02.27.640629 (2025).

6 Jumper, J. *et al.* Highly accurate protein structure prediction with AlphaFold. *Nature* **596**, 583-589, doi:10.1038/s41586-021-03819-2 (2021).

7 Pereira, G. P. *et al.* Bartender: Martini 3 Bonded Terms via Quantum Mechanics-Based Molecular Dynamics. *J Chem Theory Comput* **20**, 5763-5773, doi:10.1021/acs.jctc.4c00275 (2024).

8 P C Kroon, F. G., J Barnoud1, M van Tilburg, P C T Souza, T A Wassenaar, S J Marrink. Martinize2 and Vermouth: Unified Framework for Topology Generation. *arXiv:2212.01191*, doi:https://doi.org/10.48550/arXiv.2212.01191 (2022).

9 Wassenaar, T. A., Ingolfsson, H. I., Bockmann, R. A., Tieleman, D. P. & Marrink, S. J. Computational Lipidomics with insane: A Versatile Tool for Generating Custom Membranes for Molecular Simulations. *J Chem Theory Comput* **11**, 2144-2155, doi:10.1021/acs.jctc.5b00209 (2015).

10 Martin, E. W. *et al.* Interplay of folded domains and the disordered low-complexity domain in mediating hnRNPA1 phase separation. *Nucleic Acids Res* **49**, 2931-2945, doi:10.1093/nar/gkab063 (2021).

11 Reddy, D. N. & Prabhakaran, E. N. Synthesis and isolation of 5,6-dihydro-4H-1,3-oxazine hydrobromides by autocyclization of N-(3-bromopropyl)amides. *J Org Chem* **76**, 680-683, doi:10.1021/jo101955q (2011).

12 Mario Bianchi, A. B., Yani Christidis, Jacques Perronet, Fernando Barzaghi, Raffaele Cesana, Alberto Nencioni. Gastric anti-secretory, anti-ulcer and cytoprotective properties of substituted LY) -4-phenyl- and heteroaryl-4-oxo-2-butenoic acids. *Eur. J. Med. Chem.* **23**, 45-52 (1988).

13 Kar, S. & Madhavan, N. An Amphiphilic Peptide Carrier for HCl Transport. *Chemistry* **29**, e202301020, doi:10.1002/chem.202301020 (2023).

14 Ciancone, A. M. *et al.* Global Discovery of Covalent Modulators of Ribonucleoprotein Granules. *J Am Chem Soc* **145**, 11056-11066, doi:10.1021/jacs.3c00165 (2023).
